# Translation‐Promoting Effects of RNA Template Overhangs in the Absence of Ribosomes

**DOI:** 10.1002/anie.202520993

**Published:** 2025-12-09

**Authors:** Nikolaos Giannakopoulos, Martin Rentschler, Clemens Richert

**Affiliations:** ^1^ Institute of Organic Chemistry University of Stuttgart 70569 Stuttgart Germany

**Keywords:** Peptides, Prebiotic chemistry, RNA, Translation, Triplexes

## Abstract

Translation requires a complex set of biomacromolecules. How it evolved is an unsolved problem. Ribosome‐free, single‐nucleotide translation via coupling of 3′‐aminoacylated RNA to 5′‐phosphoramidate‐linked RNAs was shown for very short peptides. It was unclear how this process could be induced at a specific locus, regulated, and expanded. Here we show that a triplex‐forming template overhang accelerates ribosome‐free translation and increases its yield. An NMR‐monitored model study suggested that mixed anhydrides between carboxylates of amino acid residues and phosphates can contribute to efficient peptide coupling, strengthening proximity effects. Folding into triplexes at pH 6 enhances the template effect, and unfolding at pH 8 suppresses it, suggesting a simple way of achieving “inducible translation” in a system devoid of biomacromolecules. Under mild acidic conditions, doubly RNA‐linked peptides up to octamers were formed in near‐quantitative yield. Taken together, our data suggests that the growth of template strands not only allows for encoding more genetic information, but may also enhance translational fitness.

## Graphical abstract

Ribosome‐free translation of an RNA sequence into a peptide sequence can be controlled by the folding of the template strand. Overhang sequences forming a triplex structure with the templating region and the peptidoyl RNA in pH‐dependent manner increase yields up to 40‐fold through entropic effects and mixed anhydride formation, allowing for controlled, locus‐specific translation.

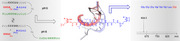

## Introduction

Locus‐specific expression of genes is a hallmark of biology. Unless the expression of genetic information starts at a specific point in the genome, and is regulated for each gene or operon, it is difficult to see how a cell can function properly. In extant biology, control over initiation, termination and expression level are among the most important processes in the cell. As a consequence, transcription factors, such as zinc fingers, make up a significant part of the genome,^[^ ^1^ ^]^ and protein complexes that induce initiation can be exceptionally complex.^[^ ^2^ ^]^ Once mRNA is produced, it should be translated in a controlled fashion. This requires the ribosome, one of the largest molecular machines of the cell. Here, initiation of translation poses a molecular recognition challenge.^[^ ^3^ ^]^ How the stages of gene expression may have been achieved in early stages of evolution has remained unclear. This is why the problem of the origin of translation has been called “one of the hardest in all evolutionary biology.”^[^ ^4^ ^]^

One approach to finding solutions to the origin of translation enigma is to deduce the earliest precursors of today's translational machinery^[^ ^5^ ^]^ (or the genetic code) through phylogenetic analyses.^[^ ^6^ ^]^ This approach does not reach back to the very earliest stages of evolution, i.e., to a time when no encoded machinery existed, for which gene sequences can be found and compared. For metabolism, cofactors can provide useful clues,^[^ ^7^ ^]^ but for transcription and translation, searches in today's genomes may not unearth sufficient information on a molecular world devoid of biomacromolecules. Instead, a “bottom‐up” approach, studying the reactions of the components of life on a purely chemical level, may be adopted.^[^ ^8^ ^]^ In the field of prebiotic chemistry, significant advances have been reported in recent years for systems devoid of any proteins or ribosomes. For example, new pathways for peptide formation have been found, based on nitrile chemistry.^[^ ^9^ ^]^ Further, aminoacylation of RNA sequences, a process that mimics the charging of tRNAs^[^ ^10^, ^11^ ^]^ has been demonstrated with stereospecificity,^[^ ^12^ ^]^ building on earlier work with RNA microhelices.^[^ ^13^ ^]^ Also, RNA‐directed peptide synthesis has been achieved via amino acid‐bearing nucleobases.^[^ ^14^ ^]^ Those findings all point to a rich chemistry that does not require fully developed ribozymes or proto‐ribosomes.^[^ ^15^ ^]^

For translation itself, i.e., a process where specific amino acids are incorporated in a growing chain according to the sequence of an RNA template, experimental systems had been lacking. Early work demonstrated peptide formation in a template‐dependent manner,^[^ ^16^ ^]^ but only homooligomers of a single amino acid were reported. Recently, however, 3′aminoacylated transfer species were shown to react with phosphoramidate‐linked 5′aminoacidyl RNAs, incorporating amino acids as determined by the base in an RNA template.^[^ ^17^ ^]^ This form of ribosome‐free translation requires no more than Watson‐Crick base pairing as read‐out principle. It involves peptide coupling between the amino group of the 3′‐aminoacylated transfer species and the carboxy group of the *N*‐linked residue. It has been demonstrated up to the pentapeptide level thus far.^[^ ^18^ ^]^ The molecular structures required can form in spontaneous reactions, as described in the literature.^[^ ^19^, ^20^, ^21^, ^22^ ^]^ Single‐nucleotide translation, as the system is called,^[^ ^17^ ^]^ was an important advance, but, in a system where an RNA sequence of modest length is the only macromolecule, achieving locus‐specific expression in the absence of proteins seemed like an impossible task.

We reasoned that a molecular recognition principle beyond simple Watson‐Crick base pairing was required to achieve the level of controlled “gene expression”. To evolve to what eventually became the ribosome, some RNA segments must have favored longer reads of primitive genes, by increasing the yield and rate of translation steps. We suspected that a segment covalently linked to the encoding sequence was a likely candidate for this next step in molecular evolution.^[^ ^23^ ^]^ Covalent attachment provides preorganization in a world without enzymes, improving specificity and avoiding diffusional loss.^[^ ^24^, ^25^ ^]^ Chain growth is a necessary process when the first RNA genomes are to be formed, making this scenario plausible.

**Figure 1:**
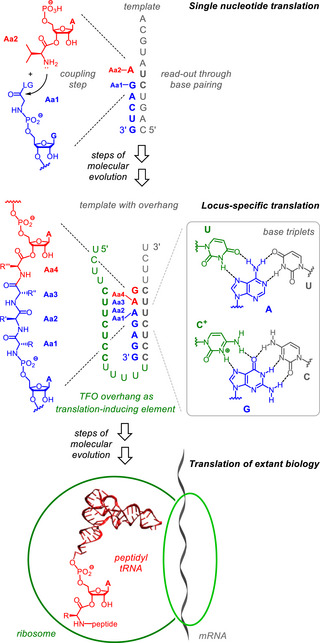
Proposed stages in the evolution of translation, starting with single nucleotide translation, and ending with translation of extant biology. Elements favoring translation are shown in green, the aminoacylated transfer species is in red, and the primer anchoring the growing peptide on the template is shown in blue. LG = leaving group, TFO = triplex‐forming oligonucleotide. The drawing of tRNA is based on pdb entry 4TRA.^[^ ^26^ ^]^

If a template overhang produced by chain growth stimulated translation by interacting with the components of single‐nucleotide translation, it could have paved the way to inducible gene expression. The simplest principle that came to mind for this interaction was triplex formation. Triple helices form when a strand binds to the major groove of RNA^[^ ^27^, ^28^ ^]^ or DNA duplexes, via Hoogsteen or reverse‐Hoogsteen base pairing.^[^ ^29^ ^]^ Triplex regions are known in genomic DNA, in the form of structural motifs called ‘H‐DNA’ for which regulatory functions are being discussed.^[^ ^30^, ^31^ ^]^ Alternatively, RNA strands may bind to DNA duplexes.^[^ ^32^ ^]^ We reasoned that once a translation‐inducing RNA segment was found, subsequent steps of evolution may have led to the ribosome (Figure 1).

Here we report that triplex‐forming RNA templates with a 5′‐overhang significantly favor single‐nucleotide translation over reactions in the absence of the overhang, allowing for translation up to at least the octapeptide level. Since translation‐promoting effects were also found for non‐triplex‐forming overhangs, a systems chemistry study involving NMR‐monitored model reactions was performed. Its results suggest that mixed anhydrides between carboxylic acids and phosphates can form that significantly reduce hydrolytic loss, making translation more efficient. Since both triplex formation and activation are pH dependent, shifts in acidity change translation levels. To the best of our knowledge, ours is the first report of a ribosome‐like effect achievable with oligoribonucleotides. The effects may have led from uncontrolled single‐nucleotide translation to a locus‐specific, controlled expression of genetic information.

## Results and Discussion

**Figure 2:**
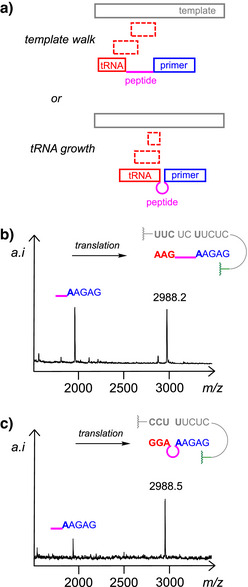
Two modes of interrogating RNA sequences of increasing length with short aminoacylated transfer strands and comparison of experimental results from the last translation step to tetrapeptide (Val)_3_Gly as a representative model reaction. a) Cartoons of the two modes of moving along the template. b) Conversion to the doubly RNA‐linked tetrapeptide product when the template walk regime is followed, as monitored by MALDI‐TOF mass spectrometry. c) Result from the corresponding assays according to the “tRNA growth” regime. Either assay result is after 24 h. Conditions: 20 µM Val_2_Gly‐AAGAG, 20 µM template, 0.12 mM AAG‐Val or GGA‐Val, 0.8 mM GMP, 0.1 M MgCl_2_, 0.2 M EDC, 1 mM phosphate buffer, pH 6, 0 °C.

The first report on single‐nucleotide translation focused on a single coupling reaction, producing a doubly RNA‐linked dipeptide, dubbed “dipeptidoyl RNA”.^[^ ^17^ ^]^ One important challenge was to achieve longer reads. Different modes of interrogating template sequences of increasing length were discussed.^[^ ^18^ ^]^ One is translocation, as seen in ribosomal translation.^[^ ^33^ ^]^ This almost certainly requires a biochemical machinery well beyond the small and medium‐size molecules considered here. Another is a “template walk” with a series of transfer‐strands that hybridize to ever more distant loci (Figure 2a). Yet another mode achieves the read‐out of subsequent bases of the template by having additional nucleotides, so that a dinucleotide “tRNA” is followed by a trinucleotide tRNA etc. (The term “tRNA” is used loosely in our studies, encompassing all aminoacylated species that undergo template‐directed coupling, ranging from aminoacylated mononucleotides to tetranucleotides.) In the former case, a stretch of unpaired template bases results, whereas in the latter case, a continuous stack of bases in the double helix is maintained, and the peptide bulges out. We tested examples of either mode experimentally. In one case, an aminoacylated trimer tRNA pairing with a distant triplet was allowed to react (Figure 2b). In the other case, the tRNA trimer that pairs next to the primer was employed (Figure 2c). Either gave successful translation as shown by MALDI‐TOF mass spectrometry, but the conversion was higher in the latter case, in line with another system showing the superiority of continuously base‐stacked systems.^[^ ^34^ ^]^ Based on these findings, we chose the “tRNA growth” system for all subsequent assays.

**Figure 3:**
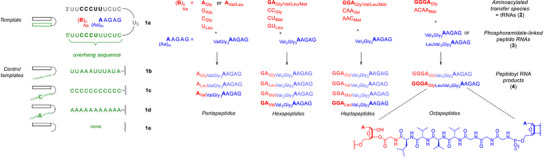
Compounds and reactions employed in the current study. Triplex‐forming and control overhang sequences are shown in green, the aminoacylated transfer species are in red, and the phosphoramidate‐linked peptido RNAs are given in blue. Throughout, RNA sequences are given in single‐letter code, and amino acid residues in three‐letter code. Each column to the right of the templates shows the compounds employed on one stage (penta‐, hexa‐, hepta‐, or octapeptides). The covalent chemistry is as shown in Figure 1. The main target sequence of our study and its precursors are in boldface at the bottom of columns. Conditions for translation assays: 20 µM peptido RNA and template, 0.8 mM aminoacylated monomers, 0.4 mM dimers, 0.12 mM trimers, or 0.06 mM tetramers, 0.8 mM CMP, 0.1 M MgCl_2_, 0.2 M EDC, 1 mM phosphate buffer pH 6, 0 °C. See Chapter 5 of the Supporting Information for additional results.

We then asked how the single‐nucleotide translation system that had been successful up to the pentapeptide level^[^ ^18^ ^]^ could be evolved further to achieve high‐yielding translation of longer sequences. The key element to be added to the reaction system was a template overhang. Further, we reasoned that additional base pairing would help to better retain the coupling partners at the template. Triple helices with gaps in the central oligopurine segment are good binding sites for nucleotides,^[^ ^35^ ^]^ engaging them via both Watson‐Crick and Hoogsteen base pairing. Folding should produce binding motifs similar to H‐DNA in cells.^[^ ^36^ ^]^ These considerations led to the compounds of Figure 3, with **1a** as the parent template with a triplex‐forming oligonucleotide (TFO) as overhang and **1b**‐**e** as control sequences. Compared to the duplex sequences studied at pH 7.5,^[^ ^18^ ^]^ the triplex allowed us to shorten the primer from seven to five nucleotides. In the figure, overhang bases are given in green, and cartoons represent template motifs.

We wished to study reactions adding the fifth, sixth, seventh, and eighth amino acid residue of the octapeptide GlyLeuVal_3_Gly_3_ via single‐nucleotide translation.^[^ ^17^, ^18^ ^]^ Each chain length was produced with more than one combination of aminoacylated tRNA and 5′‐peptido RNAs to demonstrate scope. In Figure 3, the aminoacylated transfer species (**2**) are in red, the phosphoramidate‐linked peptido primers (**3**) are in blue, and peptides in peptidoyl RNAs (**4**) are given as subscripts. The products of each translation reaction are shown below the vertical arrows. Peptidoyl RNAs found as side products of assays with mixtures are not depicted in the figure, but can be found in Chapter 6 of the Supporting Information.

**Figure 4:**
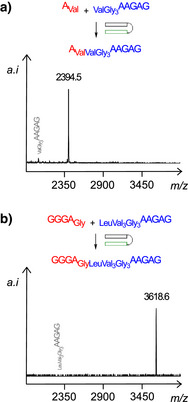
High‐yielding translation up to the octapeptide, as detected via MALDI‐TOF MS. Assays translating a) the fifth base of template **1a** after 2 d, and b) the eighth base after 3 d reaction time, when the peptido RNA primer is fully consumed. Conditions: 20 µM peptido RNA and template, 0.8 mM A‐Val or 0.06 mM GGGA‐Gly, 0.8 mM CMP, 0.1 M MgCl_2_, 0.2 M EDC, 1 mM phosphate buffer, pH 6, 0 °C.

Figure 4 shows representative MALDI‐TOF mass spectra from assays producing the peptidoyl RNA translation products on the pentapeptide and the octapeptide stage, as templated by triplex‐forming sequence **1a**. Assays were performed with the known reaction medium for single nucleotide translation,^[^ ^18^ ^]^ except that phosphate salts were included to buffer to pH 6. Those moderately acidic conditions allow for the protonation of cytosines in the TFO segment, favoring Hoogsteen base pairing in parallel triplexes.^[^ ^37^ ^]^ For each of the coupling reactions, conversion was monitored by MALDI‐TOF MS. This confirmed that longer reads of the template are feasible.

Table S10 lists the rate constants obtained by monoexponential fits to the kinetic data and the resulting half‐conversion times for 13 different combinations of aminoacylated transfer species and peptido primer reacting on template **1a**. Spectra and kinetics for all translation steps are presented in Chapter 5 of the Supporting Information. Three translation assays were performed on the pentapeptide stage, four each on the hexa‐ and heptapeptide level, and two on the octapeptide stage. For longer transfer species, concentrations were reduced due to increased hybridization strength, expected for this length range.^[^ ^38^ ^]^

All couplings occurred with near‐quantitative conversion and minimal side products, as determined by MALDI MS under conditions that allow for quantitative detection of oligonucleotides (see Chapter 5 of Supporting Information).^[^ ^39^ ^]^ In this robust translation system, the rate of each reaction depended on the amino acid residues undergoing coupling. Glycine residues at the 3′‐position of the tRNAs reacted significantly faster than sterically more hindered amino acids, such as valine or leucine. Rate constants spanned little more than two orders of magnitude in absolute value, though, with the slower reactions occurring between two amino acid residues with significant steric hindrance, but modest differences between stages of translation or peptide chain lengths were found. Importantly, all reactions required for translating the parent sequence of our study, the octapeptide GlyLeuVal_3_Gly_3_, were near‐quantitative.

**Figure 5:**
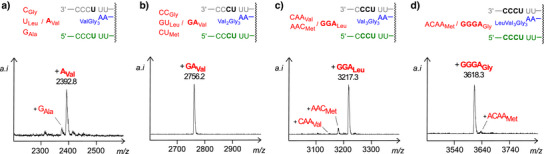
Sequence fidelity of translation. MALDI‐TOF mass spectra from assays with mixtures of aminoacylated tRNAs, a) on the pentapeptide level, b) on the hexapeptide level, c) on the heptapeptide level, and d) on the level producing the full‐length octapeptidoyl RNA. Assays were performed with 20 µM peptido RNA and template **1a**, 0.8 mM aminoacylated monomers, 0.4 mM dimers, 0.12 mM aminoacylated trimers or 0.06 mM aminoacylated tetramers, 0.8 mM CMP, 0.1 M MgCl_2_, 0.2 M EDC, 1 mM phosphate buffer pH 6 and 0 °C. Spectra were recorded after 2 d for a)/b) and 5 d for c)/d). See Chapter 6 of the Supporting Information for further details.

We then performed assays with mixtures of aminoacylated transfer species to evaluate the fidelity of translation of each of the bases translated. Mixtures of four competing aminoacyl tRNAs were employed on the monomer and dimer level, but mixtures contained three and two such species, respectively, for subsequent steps producing the heptapeptidoyl and octapeptidoyl RNA due to limiting availability of aminoacylated species. Inspection of Figure 5 shows high levels of fidelity for each stage, with the lowest base accuracy on the pentapeptide level (6% misincorporation) and heptapeptide level (13% misincorporation). These representative results give a first impression of how sequence selective the coupling reactions are, when performed with our transfer species. The conversion of the peptidoyl RNA, where *N*‐ and *C*‐terminus of the peptide are covalently attached, to the free peptide was demonstrated next (Figure S70). A shift to pH 9 cleaves only the ester linkage to the transfer strand. Exposure to acetic acid releases the free peptide.

Having demonstrated the scope of our method, we then turned to studying the molecular basis of high yielding translation with templates featuring overhangs. From the point of view of organic synthesis, the reactions are unusually successful, as “large rings” are being formed when the terminal amino acids of tRNA and peptido primer couple. Even if one counts just the peptide chain, leaving out the RNA portions of the reaction system, an octapeptide has 24 atoms in its backbone. If a bulge‐forming reaction (pink loop in Figure 2c) of this type is near quantitative, the template effect must be very significant. How strong it is can be seen from the results of control assays without template (entries 2 and 3 of Table 1), which gave little detectable conversion, compared to full conversion with **1a**. To shed light on this, assays with control templates were performed. Rate and yields of the formation of hexapeptidoyl RNA GA‐ValVal_2_Gly_3_‐AAGAG in the presence of those templates are compiled in the lower part of Table 1. We note that the true extent of yield‐improving effects is difficult to assess when the control reaction is quantitative, preventing the system from advancing to quantitative conversion.

**Table 1:** Results of translation to hexapeptidoyl RNA GA‐ValVal_2_Gly_3_‐AAGAG via coupling of GA‐Val and Val_2_Gly_3_‐AAGAG in the presence of different templates, or no template at all, together with those of control assays without an organophosphate as additive.^a)^

Entry No.	Template		Additive	Rate constant *k *× 10^2^ (h^−1^)	t_1/2_ of reaction (h)	Yield (%)^b)^
1	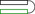	**1a**	CMP	3.8	18	quant.
2		–	–	n.d.	n.d.	<1
3		–	CMP	n.d.	n.d.	2
4^c)^	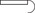	**1e**	CMP	1.1 ± 0.2	63 ± 8	21 ± 3
5	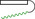	**1b**	CMP	0.8	84	48
6	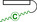	**1c**	CMP	0.4	168	53
7	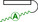	**1d**	CMP	0.5	136	55
8	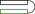	**1a**	–	1.8	38	52
9	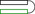	**1a**	Et‐p	3.3	21	quant.
10	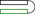	**1a**	CpC	2.6	27	93
11	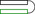	**1a**	CpC	3.0	23	97
12	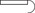	**1e**	–	0.1	473	8

^a)^ See legend to Figure 3 for conditions.

^b)^ After 96 h reaction time.

^c)^ Mean of three independent experiments ± one SD.

When we used template **1e**, which contains the U_5_ linker but no TFO segment, we detected a much more modest yield than for **1a** and a more than three‐fold slower rate (entry 4 of Table 1), confirming that the triplex‐forming segment has a strong effect. But, overhangs incapable of triplex formation with the encoding segment also had an effect. While not close to full conversion, an assay with template **1b** (overhang containing As) gave 48% conversion, while **1c** with a (C)_11_ overhang and **1d** with (A)_11_ overhang gave 53% and 55% conversion after 4 d, respectively. These reactions were slower than those templated by **1e**, despite the improved yield. An assay without CMP, which had been introduced as additive for duplex systems, assuming that it can “fill gaps” as the tRNAs move down the template,^[^ ^18^ ^]^ lowered the yield with **1a** to just 52% (entry 8 of Table 1). To pinpoint what part of the nucleotide is responsible, we then performed an assay with ethyl phosphate as additive.

With this alkyl monoester, the yield recovered, and the rate was not far from that with CMP (entry 9 of Table 1), indicating a role of phosphates in the overall reaction scheme. Similar yield‐enhancing effects were achieved with dinucleoside phosphates CpC and CpU (entries 10 and 11), indicating that both mono‐ and diesters improve reactivity. In the absence of both a template overhang and an organocatalytic mononucleotide, there is less than 10% conversion to the translation product (entry 12 of Table 1 and Figure S67). The triplex overhang effect is even larger at 10‐fold dilution. Here, without an overhang less than 2 % conversion was observed, whereas with overhang, 83% product was detected after 6 d, a 40‐fold enhancement (Figure S68).

Motivated by those findings, we decided to perform a systems chemistry analysis of coupling reactions in the presence and absence of ethyl phosphate with monitoring by NMR. We used Gly‐A to mimic the growing peptido RNA chain, glycine methyl ester (H‐Gly‐OMe) representing aminoacyl tRNA, and ethyl phosphate (Et‐p) as the organophosphate.

**Figure 6:**
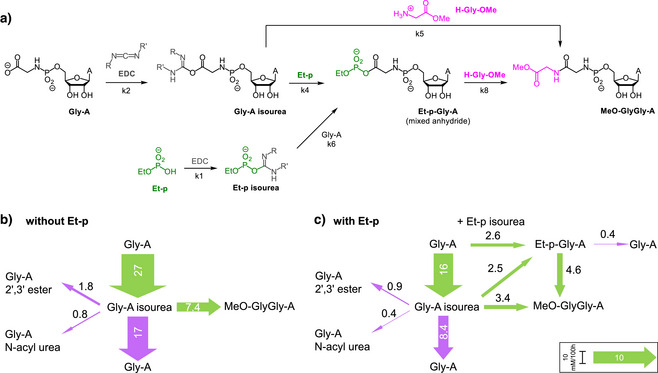
Studying the effect of an organophosphate on the peptide coupling of single‐nucleotide translation in a model system. a) Abbreviated version of the reaction system studied, R = ethyl and R' = dimethylaminopropyl chain. A full version of the scheme is shown in Figure S72. Abbreviations for compounds are given below the structures, and numbers of each rate constant are shown below the corresponding reaction arrow. b) Flux map, as obtained by integrating the coupled differential equations describing the reaction network under assay conditions for 100 h. Green arrows are for steps toward product, purple arrows are for hydrolytic loss or other side reactions (non‐productive pathways). c) Same as b), but in the presence of ethyl phosphate (Et‐p). Fluxes calculated are given as arrow width, as well as numerically on or above each arrow in mM/100 h. The inset in the lower right‐hand corner shows the width of an arrow representing a flux of 10 mM per 100 h solely for visual calibration. See Chapter 8 and Table S16 for details and the numerical values of rate constants employed.

A series of reactions with an increasing number of reaction partners, starting with just EDC in buffer, was tracked by ^1^H, ^13^C and ^31^P NMR. The kinetic data gave rate constants (*k*) for each of the reactions of the network, following the methodology of Tremmel et al.^[^ ^40^ ^]^ Figure 6a shows a simplified version of the system. The full reaction network is provided in Figure S72. After obtaining the 13 rate constants (Table S16), the coupled differential equations describing the flow of molecules through the reaction channels was integrated for a reaction time of 100 h under assay conditions, producing the flux diagrams of Figure 6b,c.

In the absence of ethyl phosphate, the main reaction channel for activated Gly‐A (the isourea) is hydrolysis (Figure 6b). In the presence of the organophosphate, an additional reaction channel opens up that leads to mixed anhydride Et‐p‐Gly‐A. Now, less than half of the activations with carbodiimide (16 mM / 100 h) end in hydrolysis (8.4 + 0.4 mM/ 100 h). So, coupling becomes more efficient as a loss channel is being minimized. Thus, mixed anhydride formation of the carboxylate with phosphate moieties can improve the yield of coupling. Interestingly, the increase in yield is not accompanied by a rate acceleration (compare values for *k*_5_ and *k*_8_ in Table S16). So, phosphates as reaction partners in the vicinity of amino acid residues lead to efficient but somewhat slower reactions. This explains the effect of control overhangs that enhance yields, but lower rates (Table 1).

A reference sample of mixed anhydride Et‐p‐Gly‐A was obtained from Gly‐A and Et‐p at pH 6 in 84% yield after chromatography. This, and the results with dinucleoside phosphates CpC and CpU as effective organophosphate additives confirm the significant effect of the organophosphates. Backbone diesters of non‐triplex‐forming RNA overhangs can form mixed anhydrides with the carboxylic acid of the *C‐*terminus of the peptide to which they are held in close proximity by the oligoribonucleotide chain. The array of phosphodiesters of the overhang is held in closer proximity when a triplex forms than when the overhang is not part of a folded complex. Mixed anhydrides probably benefit from electrostatic shielding by the negative charge of the phosphate, repelling (negatively charged) hydroxide anions, while remaining sufficiently reactive towards amines. Anhydride formation can thus reduce hydrolytic loss, making single‐nucleotide translation more efficient.

Extant translation is an exceedingly complex process, and the origin of the ribosome, its most important machine, has remained unclear.^[^ ^41^ ^]^ Our study was performed from a chemical, bottom‐up perspective. We asked how molecular recognition and reactivity can lead to the emergence of a simple, but functional form of inducible translation. Inducible gene expression is best known from transcription,^[^ ^42^ ^]^ but in the absence of DNA, gene expression may have been regulated on the level of translation. A scenario emerges from our results how molecular evolution may have produced a machinery capable of expressing information encoded in an RNA sequence in a controlled fashion.

**Figure 7:**
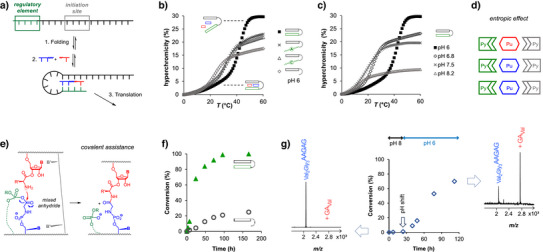
A triplex‐forming overhang as regulatory element of inducible single‐nucleotide translation. a) Proposed steps of the initiation of translation at a triplex locus.  b) UV‐melting curves of templates **1a‐e** in the presence of primer pAAGAG and transfer strand GGGA at pH 6.0. Conditions: 1 µM each of template, pAAGAG and GGGA, phosphate buffer pH 6.0, 1 M NaCl.  c) UV‐melting curves of template **1a **in the presence of pAAGAG and GGGA at different pH values. See also Tables S8 and S9.  d) Cartoon visualizing that two‐fold base pairing strengthens the template effect. e) Proposed role of mixed anhydrides in peptide formation, with backbone phosphodiester anions (green) as source of organocatalytic moieties, as demonstrated for dinucleoside phosphates CpC or CpU (entries 10 and 11 of Table 1). f) Kinetics of translation with and without triplex structure, as determined with templates **1a** and **1e** (compare Table 1). g) Inducing translation by pH shift: Kinetics and MALDI‐TOF spectra after 24 h (left) and 112 h (right) of translation to the hexapeptide at pH 8, with a shift to pH 6 after 24 h.

We wish to speculate on this scenario in the context of how the very earliest form of translation may have evolved, long before enzymes and ribosomes were available, hoping to close the wide gap between purely chemical systems and what can be deduced from the analysis of biomacromolecules of extant biology.^[^ ^5^, ^6^, ^43^ ^]^ On the chemical level, our study has identified at least two effects (Figure 7): i) Template overhangs containing sequences capable of forming triple helices with primer/transfer species and TFO overhangs give more efficient translation, most probably due to an entropic effect.^[^ ^44^ ^]^

ii) Organophosphates, including those in overhangs of scrambled or homopolymer sequences, have a yield‐enhancing effect that is probably due to mixed‐anhydride formation with the *C*‐terminus of the peptide chain. So, when the template strand grows, the likelihood of getting higher‐yielding translation increases. This increase is a possible driving force in molecular evolution. It does not require a specific function of the translation product.

Figure 7b shows UV‐melting curves of triplexes between templates **1a** or **1c‐e** and oligopurine sequences employed as primer and transfer species, at the assay pH of 6.0. Only **1a** gave the melting point shift and increase in hyperchromicity expected for fully folded structures. Figure 7c confirms that this structure fails to form when the pH leaves the acidic range. The observation that no separate point of inflection is detectable in the UV‐melting curve suggests that a concerted binding/folding event, involving all components (template/template overhang, primer and transfer strand), occurs. The all‐or‐nothing transition means that both primer and transfer strand are bound with the same strength. Because the strands are very short and no hysteresis is observed at heating and cooling rates of 1 °C min^−1^, whereas the chemical steps of translation occur on the timescale of hours or days, we have reason to believe that complex formation occurs before amino acid coupling sets in to any significant extent. How important the folded structure is for efficient translation can be seen in Figure 7f, where the kinetics of translation with and without template overhang are compared.

Overall, several principles emerge from our findings.

One such principle is the covalent attachment. For single‐nucleotide translation to occur, both reaction partners forming the new peptide bond must be covalently linked to an RNA component, and the initial product is a peptidoyl RNA, in which both *N*‐ and *C*‐terminus of the peptide are linked by a covalent bond.^[^ ^17^, ^18^ ^]^ Likewise, the triplex‐forming overhangs in the templates studied here are part of one RNA chain. This helps to avoid diffusional loss and provides preorientation of reaction partners that would otherwise be difficult to bring to react with the necessary specificity, in the absence of the directing effect of active sites.

Another principle is that the processes studied here occur under conditions favoring condensation reactions. Without condensation reactions, it is difficult to see how the first genetic material was formed from ribonucleotides, and how the first peptides were formed from amino acids. Condensation also leads to mixed anhydrides. As shown here, organophosphates provide an additional activation pathway, and they then act as leaving groups, once the kinetically stabilized anhydride encounters a suitable reaction partner. This synergy helps to explain better why phosphates are so universal in biology.^[^ ^45^ ^]^

When an RNA template grows, it becomes increasingly likely that (statistically rare) combinations of homopyrimidine encoding regions and homopyrimidine TFO segments are formed. Only this combination is capable of the triple helix formation with matching homopurine primer and transfer species studied here. That a rare combination of sequence favors translation is desirable for controlled, rather than uncontrolled expression of genetic information. If these overhangs help to promote a locus‐specific and more efficient form of translation, they can provide an advantage in fitness over more primitive systems, where translation occurs at any given primer binding site. The locus‐specific form of translation can also respond to changes in the environment, such as changes in pH, as shown in Figure 7g. An acidic pH favors triplex formation, converting cytosines to their *N3*‐protonated form required for triplets (Figure 1),^[^ ^37^ ^]^ and thus locus‐specific translation benefitting from the two yield‐enhancing factors of Figure 7d,e.

One way to obtain large complexes from smaller components as available building blocks is to make use of symmetry. In the present case, both oligopyrimidine segments of the triplexes are made up of the same two bases only (C and U), they are much shorter than what would be required, if the assembly was intermolecular, and their combined sequences are palindromic, forming a symmetrical motif. We note that the proposed earliest forms of the protoribosome are believed to be symmetrically assembled dimers,^[^ ^46^ ^]^ a form of molecular efficiency or “frugality” in a primitive genetic system. The two phenomena are not analogous, but symmetry helps in either case.

If these speculations are correct, TFO regions may have acted like a primitive transcription factor in an RNA world that did not know transcription yet, but used the read‐out of RNA sequences as the equivalent of what is now a sequence of events, including transcription and translation.^[^ ^47^ ^]^ With inducible, locus‐specific translation, controlled expression of genetic information becomes feasible, moving the system a significant step closer to the stage were protoribosomes^[^ ^48^ ^]^ exerted their catalytic effect.

## Conclusions

Our data demonstrates that single‐nucleotide translation can proceed from a simple, dipeptide producing level^[^ ^17^ ^]^ to a system capable of inducible translation up to octapeptides. With a triplex‐forming overhang, the strength of the template effect can be increased by acid‐induced folding at the encoding locus. In triplexes, near‐quantitative, sequence‐selective translation reactions occur. Entropic and organocatalytic effects appear to be underlying the increases in efficiency over translation in duplexes. Importantly, the efficiency of translation can increase upon growth of the template RNA sequence, independent of a function of the peptide product. Our findings help to bridge the wide gap between chemistry and biology in our understanding of the origin of translation.

## Supporting Information

Materials and methods, syntheses, protocols, MS and NMR spectra, UV‐melting curves, data from translation assays, and details of the systems chemistry study on mixed anhydride formation, together with additional references can be found in the Supporting Information.

## Conflict of Interests

The authors declare no conflict of interest.

## Supporting information



Supporting Information

## Data Availability

The data that support the findings of this study are available in the supplementary material of this article.

## References

[1] S. MacPherson , M. Larochelle , B. Turcotte , Microbiol. Mol. Biol. Rev. 2006, 70, 583–604, 10.1128/MMBR.00015-06. 16959962 PMC1594591

[2] P. Cramer , Nature 2019, 573, 45–54, 10.1038/s41586-019-1517-4. 31462772

[3] K. Leppek , R. Das , M. Barna , Nat. Rev. Mol. Cell Biol. 2018, 19, 158–174, 10.1038/nrm.2017.103. 29165424 PMC5820134

[4] Y. I. Wolf , E. V. Koonin , Biol. Direct 2007, 2, 14, 10.1186/1745-6150-2-14. 17540026 PMC1894784

[5] E. Westhof , N. Leontis , Angew. Chem. Int. Ed. 2000, 39, 1587–1591, 10.1002/(SICI)1521-3773(20000502)39:9<1587::AID-ANIE1587>3.0.CO;2-K. 10820444

[6] H. Grosjean , E. Westhof , Nucleic Acids Res. 2016, 44, 8020–8040, 10.1093/nar/gkw608. 27448410 PMC5041475

[7] S. A. Benner , A. D. Ellington , A. Tauer Proc. Natl. Acad. Sci. U.S.A. 1989, 86, 7054–7058, 10.1073/pnas.86.18.7054. 2476811 PMC297992

[8] K. Ruiz‐Mirazo , C. Briones , A. de la Escosura , Chem. Rev. 2014, 114, 285–366, 10.1021/cr2004844. 24171674

[9] P. Canavelli , S. Islam , M. W. Powner , Nature 2019, 571, 546–549, 10.1038/s41586-019-1371-4. 31292542

[10] N. V. Chumachenko , Y. Novikov , M. Yarus , J. Am Chem. Soc. 2009, 131, 5257–5263, 10.1021/ja809419f. 19351205 PMC2750092

[11] M. Su , C. Schmitt , Z. Liu , S. J. Roberts , K. C. Liu , K. Röder , A. Jäschke , D. J. Wales , J. D. Sutherland , J. Am. Chem. Soc. 2023, 145, 15971–15980, 10.1021/jacs.3c03931. 37435826 PMC10375532

[12] M. Su , S. J. Roberts , J. D. Sutherland , Nucleic Acids Res. 2024, 52, 11415–11422, 10.1093/nar/gkae702. 39164017 PMC11514466

[13] K. Tamura , P. R. Schimmel , Proc. Natl. Acad. Sci. U.S.A. 2006, 103, 13750–13752, 10.1073/pnas.0606070103. 16950872 PMC1564265

[14] F. Müller , L. Escobar , F. Xu , E. Węgrzyn , M. Nainytė , T. Amatov , C. Y. Chan , A. Pichler , T. Carell , Nature 2022, 605, 279–284, 10.1038/s41586-022-04676-3. 35546190 PMC9095488

[15] C. Davidovich , M. Belousoff , I. Wekselman , T. Shapira , M. Krupkin , E. Zimmerman , A. Bashan , A. Yonath , Isr. J. Chem. 2010, 50, 29–35, 10.1002/ijch.201000012. 26207070 PMC4508870

[16] R. M. Turk , M. Illangasekare , M. Yarus , J. Am. Chem. Soc. 2011, 133, 6044–6050, 10.1021/ja200275h. 21438575

[17] B. Jash , P. Tremmel , D. Jovanovic , C. Richert , Nat. Chem. 2021, 13, 751–757, 10.1038/s41557-021-00749-4. 34312504

[18] S. G. Reußwig , C. Richert , Angew. Chem. Int. Ed. 2024, 63, e202410317, 10.1002/anie.202410317. 38967604

[19] B. P. Gottikh , A. A. Krayevsky , N. B. Tarussova , P. P. Purygin , T. L. Tsilevich , Tetrahedron 1970, 26, 4419–4433, 10.1016/S0040-4020(01)93090-X. 5469480

[20] J. P. Biron , A. L. Parkes , R. Pascal , J. D. Sutherland , Angew. Chem. Int. Ed. 2005, 44, 6731–6734, 10.1002/anie.200501591. 16187390

[21] M. Jauker , H. Griesser , C. Richert , Angew. Chem. Int. Ed. 2015, 54, 14564–14569, 10.1002/anie.201506593. PMC4678511 26435376

[22] F. Welsch , E. Kervio , P. Tremmel , C. Richert , Angew. Chem. Int. Ed. 2023, 62, e202307591, 10.1002/anie.202307591. 37382466

[23] C. De Duve , Nature 2005, 433, 581–582, 10.1038/433581a. 15703726

[24] R. Pascal , L. Boiteau , A. Commeyras , Top. Curr. Chem. 2005, 259, 69–122, 10.1007/b105128.

[25] Z. Liu , G. Ajram , J. C. Rossi , R. Pascal , J. Mol. Evol. 2019, 87, 83–92, 10.1007/s00239-019-9887-7. 30788531 PMC6443614

[26] E. Westhof , P. Dumas , D. Moras , Acta Crystallogr 1988, 44, 112–123, 10.2210/pdb4tra/pdb. 3272146

[27] G. Felsenfeld , A. Rich , Biochim. Biophys. Acta. 1957, 26, 457–468, 10.1016/0006-3002(57)90091-4. 13499402

[28] M. Szabat , E. Kierzek , R. Kierzek , Sci. Rep. 2018, 8, 13023, 10.1038/s41598-018-31387-5. 30158667 PMC6115336

[29] N. T. Thuong , C. Hélène , Angew. Chem. Int. Ed. 1993, 32, 666–690, 10.1002/anie.199306661.

[30] D. Michel , G. Chatelain , Y. Herault , F. Harper , G. Brun , Cell. Mol. Biol. Res. 1993, 39, 131–140, 10.1016/j.jbc.2023.105202. 8220583

[31] A. Jain , G. Wang , K. M. Vasquez , Biochim 2008, 90, 1117–1130, 10.1016/j.biochi.2008.02.011. PMC2586808 18331847

[32] J. D. Toscano‐Garibay , G. Aquino‐Jarquin , Biochim. Biophys. Acta 2014, 1839, 1079–1083, 10.1016/j.bbagrm.2014.07.016. 25086339

[33] R. Green , H. F. Noller , Ann. Rev. Biochem. 1997, 66, 679–716, 10.1146/annurev.biochem.66.1.679. 9242921

[34] B. Jash , C. Richert , Chem. Sci. 2020, 11, 3487–3494, 10.1039/C9SC05958J. 34109020 PMC8152794

[35] C. Kröner , M. Röthlingshöfer , C. Richert , J. Org. Chem. 2011, 76, 2933–2936, 10.1021/jo2003067. 21413744

[36] C. Kröner , M. Thunemann , S. Vollmer , M. Kinzer , R. Feil , C. Richert , Angew. Chem. Int. Ed. 2014, 53, 9198–9202, 10.1002/anie.201403579. 25045108

[37] D. Leitner , W. Schröder , K. Weisz , Biochem 2000, 39, 5886–5892, 10.1021/bi992630n. 10801340

[38] M. Sosson , D. Pfeffer , C. Richert , Nucleic Acids Res. 2019, 47, 3836–3845, 10.1093/nar/gkz160. 30869145 PMC6486630

[39] D. Sarracino , C. Richert , Bioorg. Med. Chem. Lett. 1996, 6, 2543–2548, 10.1016/0960-894X(96)00465-9. 11425548

[40] P. Tremmel , H. Griesser , U. E. Steiner , C. Richert , Angew. Chem. Int. Ed. 2019, 58, 13087–13092, 10.1002/anie.201905427. PMC6852251 31276284

[41] M. R. Tirumalai , M. Rivas , Q. Tran , G. E. Fox , Microbiol. Mol. Biol. Rev. 2021, 85, e00104–21, 10.1128/MMBR.00104-21. 34756086 PMC8579967

[42] P. H. Maxwell , M. S. Wiesener , G. W. Chang , S. C. Clifford , E. C. Vaux , M. E. Cockman , C. C. Wykoff , C. W. Pugh , E. R. Maher , P. J. Ratcliffe , Nature 1999, 399, 271–275, 10.1038/20459. 10353251

[43] G. E. Fox , Cold Spring Harb. Perspect. Biol. 2010, 2, a003483, 10.1101/cshperspect.a003483. 20534711 PMC2926754

[44] A. Sievers , M. Beringer , M. V. Rodnina , R. Wolfenden , Proc. Natl. Acad. Sci. USA 2004, 101, 7897–7901, 10.1073/pnas.0402488101. 15141076 PMC419528

[45] F. H. Westheimer , Science 1987, 235, 1173–1178, 10.1126/science.2434996. 2434996

[46] I. Agmon , A. Bashan , R. Zarivach , A. Yonath , Biol. Chem. 2005, 386, 833–844, 10.1515/BC.2005.098. 16164408

[47] Z. Zhou , K. E. Gilsea , G. Felsenfeld , Proc. Natl. Acad. Sci. U.S.A. 2019, 116, 6130–6139, 10.1073/pnas.1900107116. 30867287 PMC6442552

[48] T. Bose , G. Fridkin , C. Davidovich , M. Krupkin , N. Dinger , A. H. Falkovich , Y. Peleg , I. Agmon , A. Bashan , A. Yonath , Nucleic Acids Res. 2022, 50, 1815–1828, 10.1093/nar/gkac052. 35137169 PMC8886871

