# Supplementary material for: Translation‐Promoting Effects of RNA Template Overhangs in the Absence of Ribosomes
Source: Angew Chem Int Ed Engl. 2025 Dec 9;65(4):e20993. doi: 10.1002/anie.202520993 (PMC12828457; doi:10.1002/anie.202520993)
Supplement: Supplementary file 1 — Supporting Information [file ANIE-65-e20993-s001.pdf]

Supporting Information

for manuscript entitled

Translation-Promoting Effects of RNA Template Overhangs in the Absence of Ribosomes

by

Nikolaos Giannakopoulos, Martin Rentschler and Clemens Richert

Contents

1. Materials and Methods
2. Syntheses and Protocols
3. Mass and NMR Spectra
4. UV-Melting Curve Experiments
5. Translation Assays
6. Assays With More Than One Transfer Species
7. Additional Data from Translation Study
8. Systems Chemistry Study on Mixed Anhydride Formation
9. References for Supporting Information

1. Materials and Methods

General. Organic solvents and chemicals were purchased from *Sigma-Aldrich* (Taufkirchen, Germany), *TCI* (Zwijndrecht, Belgium) or *Roth GmbH* (Karlsruhe, Germany) and were used as received from the manufacturers without further purification. The C18 reversed-phase (RP SEP-PAK VAC C18 3CC, 500 mg, 1 g or 2 g) and anion exchange cartridges (SEP-PAK VAC QMA 3CC, 500 mg or 2 g) for purification of oligoribonucleotides were purchased from *Waters* (Milford, Massachusetts, USA). Oligoribonucleotides were synthesized on an H-2 DNA/RNA synthesizer from *K&A* (Schaafheim, Germany), following the protocol recommended by the manufacturer. The required TBDMS 2'-protected ribonucleoside phosphoramidites were purchased from *ChemGenes* (Wilmington, MA, USA) or *Future Synthesis* (Poznań, Poland) and immobilized nucleosides on controlled-pore-glass (pore size 1000 Å, loading 20-30 μmol/g) were from *Sigma-Aldrich* (Taufkirchen, Germany). The phosphorylation reagent 2-[2-(4,4'-dimethoxytrityloxy)ethylsulfonyl]ethyl-(2-cyanoethyl)-(*N*,*N*diisopropyl)phosphoramidite was synthesized in-house according to a literature procedure.[S1] The RNA templates were purchased from *Biomers* (Ulm, Germany) in HPLC-purified form and desalted by C18 RP chromatography to remove traces of acetate salts before usage.

Sequences of RNA Templates

5'-UUCCCUUUCUCUUUUUCUCUUUCCCUU-3' (**1a**)
5'-UUAAAUUUAUAUUUUUCUCUUUCCCUU-3' (**1b**)
5'-CCCCCCCCCCCUUUUUCUCUUUCCCUU-3' (**1c**)
5'-AAAAAAAAAAAUUUUUCUCUUUCCCUU-3' (**1d**)
5'-UUCCCUUUCUCUUUUU-3' (**1e**)
5'-UUCCUCUUCUCUUUUUCUCUUCUCCUU-3' (**1f**)
5'-CCCUCUCUUCUCUUUUUCUCUUCUCUCCC-3' (**1g**)

MALDI-TOF Mass Spectrometry. All MALDI-TOF mass spectra were recorded on a MICROFLEX spectrometer from *Bruker Daltonics* (Bremen, Germany) with an N_2 laser (337 nm). Measurements in the linear negative mode were performed, using a mixture of 0.3 M trihydroxyacetophenone (THAP) in ethanol and 0.1 M aqueous ammonium citrate (CT) (2:1, *v/v*) as matrix/co-matrix. A mixture of oligonucleotides with known masses served as an

external calibration standard. For the characterization of the samples, an aliquot of 0.5 μL was mixed with 0.5 μL of the matrix solution on a stainless-steel target (MSP 96) from *Bruker Daltonics* (Bremen, Germany) and allowed to crystallize. For the analysis and monitoring of assays, aliquots of the reaction's solutions (0.25 μL) were diluted in water (2.5 μL) and placed on a few beads of DOWEX cation-exchange resin (NH_4^+ form) for 30 min before being inserted in the source. Measurements were carried out with flexControl software, and the analysis of spectra used the program flexAnalysis, version 3.4. The $[M-H]^-$ ions detected are given as m/z.

ESI-TOF Mass Spectrometry. ESI-MS (Electrospray Ionization Mass Spectrometry) spectra were recorded on a micrOTOF-Q mass spectrometer from *Bruker Daltonics* (Bremen, Germany) in positive or negative mode, using the following parameters: capillary voltage 4500 V; nebulizer gas pressure 0.3 bar; end plate offset voltage: -500 V; drying gas velocity 4.0 L/min, and heater temperature: 200 °C.

NMR Spectroscopy. The NMR spectra were recorded on a Bruker Avance III HD 400 or 500 NanoBay NMR spectrometer (400 MHz or 500 MHz resonance frequence for ^1H) at 298 K or a Bruker Avance III HD 700 NMR spectrometer (700 MHz for ^1H) at 298 K or 277 K (Bruker, Rheinstetten, Germany). All samples were prepared using deuterium oxide (D_2O; 99.9% deuterated) from *Euriso-Top* (Saint-Aubin, France) or dimethyl sulfoxide-d_6 (DMSO-d_6; 99.9% deuterated) from *Sigma Aldrich* (Schnelldorf, Germany) as solvents. Chemical shifts (δ) are given in parts per million (ppm) and coupling constants (*J*) in Hertz (Hz). The multiplicities of the signals are abbreviated as follows: s for singlet, d for dublet, t for triplet, q for quartet and m for multiplet. For the analysis of the NMR spectra the software Topspin version 4.1.3 was used.

UV-Vis Spectroscopy. Yields and concentrations of the oligonucleotides synthesized were determined by UV/Vis spectroscopy using a NanoDrop ND-1000 UV/Vis spectrometer from *Thermo Fisher Scientific* (Wilmington, USA). For calibration, a control measurement was carried out with water. The diluted samples (1 μL) were pipetted onto the sample platform and the absorptions were measured at a wavelength of λ = 260 nm. The concentrations of the samples were determined using Lambert-Beer's law (1),

$$E = \varepsilon \ c \ d \qquad (1)$$

where E corresponds to the extinction, c to the concentration, ε to the extinction coefficient, and d to the depth of the sample, through which the light travels.

Automatic Chromatography. Chromatographic purification of nucleotide derivatives for kinetic studies monitored by NMR was performed on a preparative Biotage column of (type 30 g C18 D Duo, 100 Å, 30 µm), eluting with HPLC grade water/acetonitrile (up to 20% CH_3CN), while detecting at λ = 260/280 nm.

HPLC. Purification of 5'-peptido RNAs was carried out by RP-HPLC, with a Waters HPLC 1525 binary pump system from *Waters* (Milford, USA). The analysis of the HPLC traces was done using the manufacturer's software. An analytical C18 column, EC 250 mm/4.6 mm Nucleosil 120–5, from *Macherey-Nagel* (Düren, Germany) was used. The column was operated at a temperature of 55 °C, and the flow rate was set to 1 mL/min. The crude products were eluted with a gradient of acetonitrile in a 0.1 M triethylammonium acetate solution (TEAA, pH 7.0) as solvent A. Detection was at a wavelength of λ = 260 nm, and the fractions eluted were analyzed using MALDI-TOF mass spectrometry.

UV-Melting. Temperature-dependent UV-Vis absorption measurements were carried out using a Lambda 10 or Lambda 25 spectrometer from *Perkin Elmer* (Waltham, USA). The data were analyzed using the software programs Templab (version 2.0, *Perkin Elmer*) and UV Winlab (versions 2.85.04 and 6.0.4, *Perkin Elmer*). The UV melting curves were recorded at 260 nm in quartz cuvettes at a heating and cooling rate of 1 °C/min. The concentration of oligonucleotides was 1 µM. A phosphate buffer (10 mM), with NaCl (1 M) at pH 6.0, 6.8, 7.5 or 8.2 was used as buffer system. For each melting curve experiment, two heating and cooling runs were measured. The melting points were determined using the UV Winlab 2.0. They are the average of the extrema of the first derivative. The data sets were smoothed (90-point) using the UV Winlab software. Hyperchromicities were determined by calculating the differences in absorption between high- and low-temperature baseline values and dividing by the low-temperature absorption.

2. Syntheses and Protocols

Solid-Phase synthesis of oligoribonucleotides (General Protocol A).

The synthesis of oligoribonucleotides was carried out on a 1 µmol or 10 µmol scale on an H-2 synthesizer (*K&A*, Schaafheim, Germany), following the synthesis protocol of the manufacturer via the phosphoramidite method. The 10 µmol scale synthesis of dimer 5'-GA-3' is described below and is representative. The cpg loaded with the fully assembled oligoribonucleotide strand was dried under reduced pressure in a desiccator for 30 min. The cleavage of the oligoribonucleotide from the solid support, as well as the deprotection of acyl protecting groups, was induced by treating with ammonium hydroxide/methyl amine solution (AMA, 5 mL, 1:1, *v*/*v*) at 60 °C for 20 min. The supernatant was collected and the cpg washed with water (3×5 mL). The collected supernatants were combined and freed from excess ammonia and methylamine by a stream of nitrogen (30 min), followed by lyophilization. Triethylamine trihydrofluoride (4 mL) was added to the lyophilized solid to cleave 2'-TBDMS protecting groups. After 6 h, methoxytrimethylsilane (15 mL) was added, the mixture was vortexed for 5 min, and the resulting suspension centrifuged for 5 min at 4000 rpm. The precipitated crude was purified on a SEP-PAK VAC QMA 3CC cartridge (2 g) pre-equilibrated with water (20 mL). For this, the crude was dissolved in water (1 mL), applied to the cartridge and eluted using a gradient of ammonium carbonate $(NH_4)_2CO_3$ in water. Then, RP-chromatography was carried out using a RP SEP-PAK VAC C18 3CC cartridge (2 g) pre-equilibrated with acetonitrile (5 mL) and water (20 mL). The product was diluted in NaCl solution (5 M, 0.5 mL), applied onto the cartridge, washed with NaCl solution (1 M, 5 mL) and water (15 mL), and eluted with a gradient of acetonitrile in water. Product containing fractions were identified by MALDI-TOF mass spectrometry and quantified by UV absorption using known extinction coefficients of nucleosides and nucleotides.[S2] Dimer 5'-GA-3' was isolated as colorless solid. Yield: 58% (5.8 µmol).

Table S1. Data for RNA strands prepared via solid-phase synthesis.

RNA sequence	yield (%)	$[M-H]^-$ m/z calc.	$[M-H]^-$ m/z found
5'-GA-3'	58	610.4	609.9
5'-CU-3'	49	548.3	548.7
5'-GU-3'	43	588.4	588.1
5'-CC-3'	54	547.3	546.6
5'-GGA-3'	45	954.8	954.4
5'-AGG-3'	36	954.8	954.6
5'-AAC-3'	39	900.2	899.1
5'-CAA-3'	36	900.2	899.6
5'-ACAA-3'	25	1229.2	1228.6
5'-GGGA-3'	35	1299.9	1300.4
5'-pAAGAG-3'	27	1693.8	1694.3

Solid-phase peptide synthesis (General Protocol B).

Peptides were prepared via solid-phase synthesis. The synthesis of H-GlyValVal-OH is described below and is representative. In order to perform multiple coupling cycles on solid support, a Vac-Man laboratory vacuum manifold (*Promega*, Walldorf, Germany) and a *L*-valine 2-chlorotrityl resin (H-Val-2ClTrt, 200 mg, 0.79 mmol/g) were used. The resin was placed in a reactor for peptide synthesis with a capacity of 20 mL and treated with DMF (2 mL) for 30 min (swelling). *N*-Fmoc-protected valine (141 mg, 474 µmol, 3 eq.), HBTU (161 mg, 474 µmol, 3 eq.), and HOBt (64 mg, 474 µmol, 3 eq.) were dissolved in DMF (1 mL). Then, DIPEA (107 µL, 632 µmol, 4 eq.) was added to the solution, and the mixture was added to the resin and allowed to react for 1 h. The resin was then washed with DMF, DCM and DMF (3×3 mL each). The Fmoc protecting group was removed using a solution of 20% piperidine in DMF (2×2 mL, 10 min each). The resin was then washed with DMF, DCM and DMF again (3×3 mL each). These steps were repeated with an activation solution containing *N*-Fmoc-protected glycine (180 mg, 474 µmol, 3 eq.), HBTU (180 mg, 474 µmol, 3 eq.), HOBt (64 mg, 474 µmol, 3 eq.) and DIPEA (107 µL, 632 µmol, 4 eq.) dissolved in DMF (1 mL) until the desired peptide coupling had occurred. After completion of the chain assembly, the resin was dried for 30 min at reduced pressure. The cleavage from the solid support was carried out by adding a solution of TFA/TIS/H_2O (95:2.5:2.5, 2 mL) to the resin for 2 h. The supernatant was transferred to a

round-bottom flask, and the remaining resin was washed with TFA/TIS/H_2O (95:2.5:2.5, 1 mL), followed by DCM (2×2 mL). The solutions were combined and excess of TFA was removed with a stream of nitrogen (1 h). The remaining solvent was concentrated under reduced pressure, the crude mixture added to ice-cold methyl tert-butyl ether (MTBE, 15 mL) and the mixture kept at 0 °C for 15 min. The solution was centrifuged for 5 min at 4000 rpm and the precipitate washed with ice-cold diethyl ether (2×1 mL). The colorless solid was then dissolved in water and lyophilized to dryness. Yield: 94% (40 mg, 149 µmol).

HRMS (ESI): m/z, calculated for $C_{12}H_{23}N_3O_4$, $[M+H]^+$: 274.1789, found: 274.1783.

^{1}H NMR (700 MHz, $DMSO_{d6}$, 298 K): δ = 4.33 (d, J = 7.2, 1H, αH-Val), 4.05 (d, J = 7.2, 1H, αH-Val2), 3.58 (s, 2H, αH-Gly3), 2.52-2.38 (m, 1H, β-Val1), 1.98-1.88 (m, 1H, β-Val2), 0.86 (m, 12H, γH-Val).

H-GlyGlyGlyVal-OH. The tetrapeptide was synthesized according to General Protocol B, using *L*-valine 2-chlorotrityl resin (H-Val-2ClTrt, 200 mg, 0.79 mmol/g), activated glycine building block solutions containing *N*-Fmoc-protected glycine (141 mg, 474 µmol, 3 eq.), HBTU (180 mg, 474 µmol, 3 eq.), HOBt (64 mg, 474 µmol, 3 eq.) and DIPEA (107 µL, 632 µmol, 4 eq.), dissolved in DMF (1 mL). The pentapeptide was isolated as colorless solid. Yield: 95% (43 mg, 150 µmol).

HRMS (ESI): m/z, calculated for $C_{11}H_{21}N_4O_5$, $[M+H]^+$: 289.1435, found: 289.1434.

^{1}H NMR (700 MHz, D_2O, 298 K): δ = 4.19 (d, J = 5.92, 1H, αH-Val), 3.94 (overlapping s, 2H, αH-Gly1), 3.91 (s, 2H, αH-Gly3), 3.80 (overlapping, s, 2H, αH-Gly2), 2.10 (m, 1H, β-Val), 0.84 (m, 6H, γH-Val).

H-GlyGlyGlyValVal-OH. The pentapeptide was synthesized according to General Protocol B, using *L*-valine 2-chlorotrityl resin (H-Val-2ClTrt, 200 mg, 0.79 mmol/g), activated glycine building block solutions containing *N*-Fmoc-protected glycine (141 mg, 474 µmol, 3 eq.), HBTU (180 mg, 474 µmol, 3 eq.), HOBt (64 mg, 474 µmol, 3 eq.), DIPEA (107 µL, 632 µmol, 4 eq.), dissolved in DMF (1 mL), and activated valine building block solution containing *N*-Fmoc-protected valine (161 mg, 474 µmol, 3 eq.), HBTU (180 mg, 474 µmol, 3 eq.), HOBt (64 mg, 474 µmol, 3 eq.), DIPEA (107 µL, 632 µmol, 4 eq.), dissolved in DMF (1 mL). The pentapeptide was isolated as colorless solid. Yield: 97% (65 mg, 153 µmol).

HRMS (ESI): m/z, calculated for $C_{16}H_{29}N_5O_6$, $[M+H]^+$: 388.2188, found: 388.2186.

^{1}H NMR (700 MHz, D_2O, 298 K): δ = 4.08 (d, J = 8.30, 1H, αH-Val1), 4.05 (d, J = 7.76, 1H, αH-Val2), 3.91 (s, 2H, αH-Gly1), 3.88 (overlapping s, 2H, αH-Gly3), 3.83 (s, 2H, αH-Gly2), 3.80 (s, 2H, αH-Gly2), 2.05 (m, 2H, β-Val), 0.84 (m, 12H, γH-Val).

H-GlyGlyGlyValValVal-OH. The hexapeptide was synthesized according to General Protocol B, using *L*-valine 2-chlorotrityl resin (H-Val-2ClTrt, 200 mg, 0.79 mmol/g), activated glycine building block solutions containing *N*-Fmoc-protected glycine (141 mg, 474 µmol, 3 eq.), HBTU (180 mg, 474 µmol, 3 eq.), HOBt (64 mg, 474 µmol, 3 eq.), DIPEA (107 µL, 632 µmol, 4 eq.), dissolved in DMF (1 mL), and activated valine building block solutions containing *N*-Fmoc-protected valine (161 mg, 474 µmol, 3 eq.), HBTU (180 mg, 474 µmol, 3 eq.), HOBt (64 mg, 474 µmol, 3 eq.), DIPEA (107 µL, 632 µmol, 4 eq.), dissolved in DMF (1 mL). The hexapeptide was isolated as colorless solid. Yield: 89% (70 mg, 141 µmol).

HRMS (ESI): m/z, calculated for $C_{21}H_{38}N_6O_7$, $[M+H]^+$: 487.2802, found: 487.2809.

^{1}H NMR (700 MHz, D_2O, 298 K): δ = 4.15 (d, J = 8.30, 1H, αH-Val1), 4.13 (overlapping d, J = 7.45, 1H, αH-Val3), 4.13 (overlapping d, J = 7.45, 1H, αH-Val2), 3.95 (s, 2H, αH-Gly1), 3.90 (overlapping s, 2H, αH-Gly3), 3.82 (s, 2H, αH-Gly2), 2.04 (m, 3H, β-Val), 0.86 (m, 18H, γH-Val).

H-GlyGlyGlyValValValVal-OH. The heptapeptide was synthesized according to General Protocol B, using *L*-valine 2-chlorotrityl resin (H-Val-2ClTrt, 200 mg, 0.79 mmol/g), activated glycine building block solutions containing *N*-Fmoc-protected glycine (141 mg, 474 µmol, 3 eq.), HBTU (180 mg, 474 µmol, 3 eq.), HOBt (64 mg, 474 µmol, 3 eq.), DIPEA (107 µL, 632 µmol, 4 eq.), dissolved in DMF (1 mL), and activated valine building block solutions containing *N*-Fmoc-protected valine (161 mg, 474 µmol, 3 eq.), HBTU (180 mg, 474 µmol, 3 eq.), HOBt (64 mg, 474 µmol, 3 eq.), DIPEA (107 µL, 632 µmol, 4 eq.), dissolved in DMF (1 mL). The heptapeptide was isolated as colorless solid. Yield: 68% (63 mg, 108 µmol).

HRMS (ESI): m/z, calculated for $C_{26}H_{47}N_7O_8$, $[M+H]^+$: 586.3486, found: 586.3483.

^{1}H NMR (700 MHz, D_2O, 298 K): δ = 4.08 (d, J = 7.30, 1H, αH-Val1), 4.04 (overlapping d, J = 7.50, 1H, αH-Val4), 4.02 (overlapping d, J = 7.50, 1H, αH-Val3), 4.02 (overlapping d,

J = 8.0, 1H, αH-Val2) 3.94 (s, 2H, αH-Gly1), 3.87 (s, 2H, αH-Gly3), 3.79 (s, 2H, αH-Gly2), 1.98 (m, 4H, β-Val), 0.82 (m, 24H, γH-Val).

H-GlyGlyGlyValValValLeu-OH. The heptapeptide was synthesized according to General Protocol B, using *L*-leucine 2-chlorotrityl resin (H-Leu-2ClTrt, 200 mg, 0.65 mmol/g), activated glycine building block solutions containing *N*-Fmoc-protected glycine (132 mg, 390 μmol, 3 eq.), HBTU (148 mg, 390 μmol, 3 eq.), HOBt (53 mg, 390 μmol, 3 eq.), DIPEA (90 μL, 520 μmol, 4 eq.), dissolved in DMF (1 mL), and activated valine building block solutions containing *N*-Fmoc-protected valine (116 mg, 390 μmol, 3 eq.), HBTU (148 mg, 390 μmol, 3 eq.), HOBt (53 mg, 390 μmol, 3 eq.), DIPEA (90 μL, 520 μmol, 4 eq.), dissolved in DMF (1 mL). The heptapeptide was isolated as colorless solid. Yield: 76% (59 mg, 99 μmol).

HRMS (ESI): m/z, calculated for $C_{27}H_{49}N_7O_8$, $[M+H]^+$: 600.3653, found: 600.3658.

^{1}H NMR (700 MHz, D_2O, 298 K): δ = 4.25 (t, *J* = 7.50, 1H, αH-Leu), 4.03 (d, *J* = 7.56, 1H, αH-Val1), 4.00 (d, *J* = 6.95, 1H, αH-Val2), 3.98 (d, *J* = 7.18, 1H, αH-Val3), 3.94 (s, 2H, αH-Gly1), 3.88 (s, 2H, αH-Gly3), 3.79 (s, 2H, αH-Gly2), 1.92 (m, 3H, β-Val), 1.55 (overlapping m, 3H, β,γ-Leu), 0.83 (m, 24H, γH-Val, δH-Leu).

Synthesis of Peptido RNA (General Protocol C).

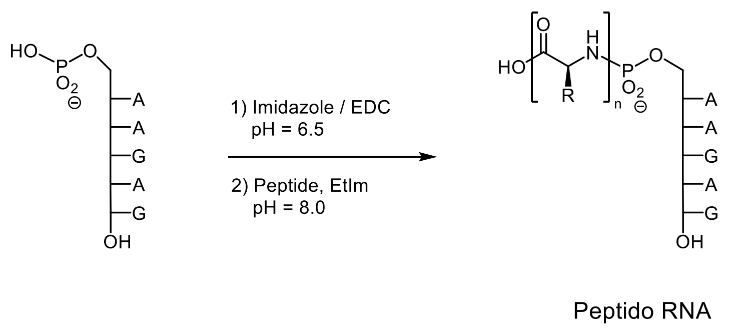

Scheme S1. Synthesis of peptido RNAs with 5'-pAAGAG-3' as RNA sequence.

The assembly of 5'-peptido RNAs followed the procedure of Räuchle et al.[S3] The protocol below is for the synthesis of aminoacidyl RNA Gly-pAAGAG-3' and is representative. Imidazole (20 mg, 0.3 mmol, 0.3 mM) and EDC hydrochloride (38 mg, 0.3 mmol, 0.3 mM) were dissolved in water (1 mL), and the pH of the solution was adjusted to 6.5. The RNA strand (5'-pAAGAG-3', 100 nmol) was dissolved in 30 μL of the imidazole/EDC solution and kept at

25 °C. The activation of the 5'-phosphate was monitored by MALDI-TOF MS. After 3 h, the reaction mixture was poured into a pre-chilled $NaClO_4$ solution (2 mL, 0.1 M) in acetone and diethyl ether (3:2, *v*/*v*), and kept at -20 °C for 10 min, followed by centrifugation (4000 rpm, 5 min). The colorless solid precipitate was dried under air for 30 min. Subsequently, a solution of glycine (6.8 mg, 0.09 mmol, 0.3 M) in water (0.1 mL) was prepared and its pH adjusted to 7.5. The peptide solution was then added to the activated oligoribonucleotide, treated with 1-ethylimidazole (EtIm, 0.95 µL, 100 mM), and the solution incubated at 25 °C for 16 h. The purification of the crude was carried out by RP-HPLC. The column was operated at 55 °C and a flow rate of 1 mL/min was set. The sample was diluted in water (200 µL) and the desired Gly-pAAGAG-3' purified using a gradient of acetonitrile in 0.1 M TEAA solution (pH 7.0). The aminoacidyl RNA eluted with 7% acetonitrile in TEAA solution at a retention time of t_R = 17 min. Pure fractions were combined, characterized by MALDI-TOF MS and lyophilized to dryness. The dry sample was then subjected to C18 cartridge chromatography. First, the product was dissolved in NaCl solution (5 M, 0.5 mL), loaded onto the C18 cartridge [500 mg, pre-equilibrated with acetonitrile (1 mL) and water (10 mL)], washed with NaCl solution (1 M, 2 mL), followed by water (10 mL), and eluted with 10% acetonitrile in water. Yield: 18% (18 nmol).

MALDI-TOF MS: m/z, calculated for $C_{52}H_{60}N_{26}O_{34}P_5$, $[M-H]^-$: 1748.1, found: 1748.6.

Dipeptido RNA ValGly-pAAGAG-3'. The target compound was synthesized according to General Protocol C. The activated RNA strand (5'-pAAGAG-3', 100 nmol) was treated with a solution of H-GlyVal-OH (0.1 mL, 0.3 M) and 1-EtIm (100 mM) at pH 8 and kept at 25 °C for 14 h. The dipeptido RNA was purified by RP-HPLC (55 °C, flow 1 mL/min, 0.1 M TEAA), eluted with 8% acetonitrile at a retention time of t_R = 18-19 min, and from the subsequent C18 cartridge with 10% acetonitrile in water. Yield: 19% (19 nmol).

MALDI-TOF MS: m/z, calculated for $C_{57}H_{69}N_{27}O_{35}P_5$, $[M-H]^-$: 1849.2, found: 1849.0.

Tripeptido RNA Val_2Gly-pAAGAG-3'. The target compound was synthesized according to General Protocol C. The activated RNA strand (5'-pAAGAG-3', 100 nmol) was treated with a solution of H-GlyValVal-OH (0.1 mL, 0.3 M) and 1-EtIm (100 mM) at pH 8.5 and kept at 25 °C for 14 h. The tripeptido RNA was purified by RP-HPLC (55 °C, flow 1 mL/min, 0.1 M TEAA),

eluted with 9% acetonitrile at a retention time of t_R = 20-21 min, and from the subsequent C18 cartridge with 10% acetonitrile in water. Yield: 10% (10 nmol).

MALDI-TOF MS: m/z, calculated for $C_{62}H_{78}N_{28}O_{36}P_5$, $[M-H]^-$: 1948.4, found: 1948.2.

Tetrapeptido RNA ValGly$_3$-pAAGAG-3'. The target compound was synthesized according to General Protocol C. The activated RNA strand (5'-pAAGAG-3', 100 nmol) was treated with a solution of H-GlyGlyGlyVal-OH (0.1 mL, 0.3 M) and 1-EtIm (100 mM) at pH 8.5 and kept at 25 °C for 14 h. The tetrapeptido RNA was purified by RP-HPLC (55 °C, flow 1 mL/min, 0.1 M TEAA), eluted with 10% acetonitrile at a retention time of t_R = 22−24 min, and from the subsequent C18 cartridge with 10% acetonitrile in water. Yield: 17% (17 nmol).

MALDI-TOF MS: m/z, calculated for $C_{61}H_{74}N_{29}O_{37}P_5$, $[M-H]^-$: 1963.6, found: 1962.7.

Pentapeptido RNA Val$_2$Gly$_3$-pAAGAG-3'. The target compound was synthesized according to General Protocol C. The activated RNA strand (5'-pAAGAG-3', 100 nmol) was treated with a solution of H-GlyGlyGlyValVal-OH (0.1 mL, 0.3 M) and 1-EtIm (100 mM) at pH 8.5 and kept at 25 °C for 14 h. The pentapeptido RNA was purified by RP-HPLC (55 °C, flow 1 mL/min, 0.1 M TEAA), eluted with 11−11.5% acetonitrile at a retention time of t_R = 25−28 min, and from the subsequent C18 cartridge with 10% acetonitrile in water. Yield: 22% (22 nmol).

MALDI-TOF MS: m/z, calculated for $C_{66}H_{84}N_{30}O_{38}P_5$, $[M-H]^-$: 2062.5, found: 2061.9.

Hexapeptido RNA Val$_3$Gly$_3$-pAAGAG-3'. The target compound was synthesized according to General Protocol C. The activated RNA strand (5'-pAAGAG-3', 100 nmol) was treated with a solution of H-GlyGlyGlyValValVal-OH (0.1 mL, 0.3 M) and 1-EtIm (100 mM) at pH 8.5 and kept at 25 °C for 16 h. The hexapeptido RNA was purified by RP-HPLC (55 °C, flow 1 mL/min, 0.1 M TEAA), eluted with 12−12.5% acetonitrile at a retention time of t_R = 30−33 min, and from the subsequent C18 cartridge with 10% acetonitrile in water. Yield: 12% (12 nmol).

MALDI-TOF MS: m/z, calculated for $C_{71}H_{93}N_{31}O_{39}P_5$, $[M-H]^-$: 2161.8, found: 2160.7.

Heptapeptido RNA Val$_4$Gly$_3$-pAAGAG-3'. The target compound was synthesized according to General Protocol C. The activated RNA strand (5'-pAAGAG-3', 100 nmol) was treated with a solution of H-GlyGlyGlyValValValVal-OH (0.1 mL, 0.3 M) and 1-EtIm (100 mM) at pH 8.3 and kept at 25 °C for 16 h. The heptapeptido RNA was purified by RP-HPLC (55 °C, flow 1 mL/min, 0.1 M TEAA), eluted with 13−13.5% acetonitrile at a retention time of t_R = 35−37 min, and from the subsequent C18 cartridge with 10% acetonitrile in water. Yield: 13% (13 nmol).

MALDI-TOF MS: m/z, calculated for $C_{76}H_{102}N_{32}O_{40}P_5$, $[M-H]^-$: 2259.7, found: 2260.6.

Heptapeptido RNA LeuVal$_3$Gly$_3$-pAAGAG-3'. The target compound was synthesized according to General Protocol C. The activated RNA strand (5'-pAAGAG-3', 100 nmol) was treated with a solution of H-GlyGlyGlyValValValLeu-OH (0.1 mL, 0.3 M) and 1-EtIm (100 mM) at pH 8.0 and kept at 25 °C for 16 h. The heptapeptido RNA was purified by RP-HPLC (55 °C, flow 1 mL/min, 0.1 M TEAA), eluted with 13−13.5% acetonitrile at a retention time of t_R = 35−37 min, and from the subsequent C18 cartridge with 10% acetonitrile in water. Yield: 19% (19 nmol).

MALDI-TOF MS: m/z, calculated for $C_{77}H_{104}N_{32}O_{40}P_5$, $[M-H]^-$: 2273.7, found: 2274.8.

Synthesis of 2'/3'-aminoacylated transfer species (General Protocol D).

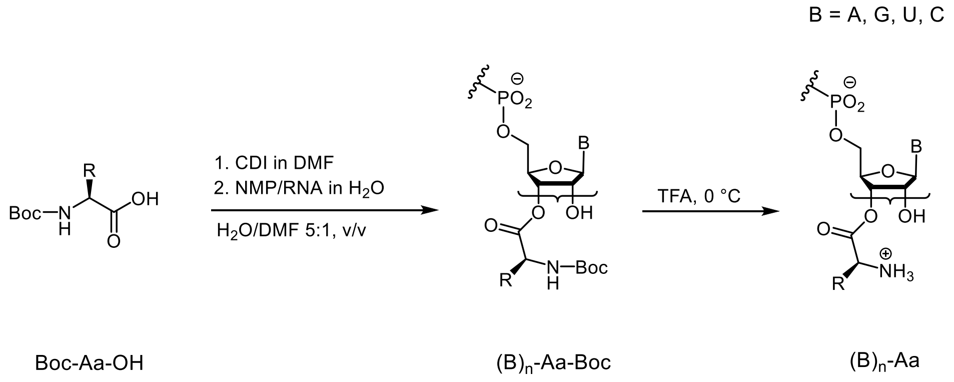

Scheme S2. Synthesis of 2'/3'-aminoacylated transfer species via two-step reaction sequence.

Synthesis of *N*-Boc-protected transfer species. The synthesis of *N*-Boc-protected 2'/3'-aminoacylated transfer strands is a modification of the procedures of Jash et al.[S4] and Reußwig et al.[S5] The synthesis of A-Gly-Boc is described below and is representative for the preparation

of 2'/3'-aminoacylated mononucleotides. Samples of *N*-Boc-protected glycine (Boc-Gly-OH, 19.3 mg, 110 µmol, 2 eq.) and 1,1-carbonyldiimidazole (CDI, 21.4 mg, 138 µmol, 2.5 eq.) were dissolved in dry DMF (0.1 mL) under nitrogen and allowed to react for 30 min. The solution of the activated amino acid was added to adenosine-5'-monophosphate (AMP, 19.1 mg, 55 µmol), which had previously been dissolved in water (0.5 mL), producing a solution in a mixture of DMF:H_2O (1:5, *v*/*v*). The reaction mixture was kept at 25 °C for 5 h, followed by the addition of water (10 mL, adjusted to pH 4.5 with 1 M HCl) and lyophilization.

The synthesis of GA-Gly-Boc is described below and representative for the preparation of 2'/3'-aminoacylated di-, tri- and tetranucleotides. *N*-Boc-protected glycine (Boc-Gly-OH, 4.9 mg, 28 µmol, 20 eq.) and CDI (5.7 mg, 35 µmol, 25 eq.) were dissolved in dry DMF (0.2 mL) under nitrogen and allowed to react for 30 min. Then, 20 µL of the activated glycine solution was added to 5'-GA-3' (0.85 mg, 1.4 µmol, 1 eq.) in water (0.1 mL) and kept at 25 °C. After 2.5 h, additional aliquots of water (0.1 mL) and activated glycine solution (20 µL) were added to the reaction mixture. The conversion to the desired 2'/3'-aminoacylated transfer strand was monitored by MALDI-TOF MS. After a total reaction time of 5 h, water (3 mL, adjusted to pH 4.5 with 1 M HCl) was added, and the solution subjected to lyophilization.

Crude products from either procedure were purified by C18 cartridge (1 g) chromatography. The crude was dissolved in NaCl solution (5 M, 0.5 mL) and applied onto the pre-equilibrated cartridge. The cartridge was then washed with NaCl solution (1 M, 5 mL) and water (15 mL, adjusted to pH 4.5 with 1 M HCl), and the desired compounds were eluted with a gradient of acetonitrile (0-20%) in water (adjusted to pH 4.5 with 1 M HCl). Pure fractions were identified via UV-vis and MALDI-TOF MS measurements and lyophilized to dryness.

Boc-deprotection of charged transfer species. The deprotection of A-Gly-Boc to A-Gly is described below and representative for deprotection procedures. A sample of A-Gly-Boc (250 nmol) was treated with trifluoracetic acid (TFA, 50 µL) and kept at 0 °C for 20 min. Excess of TFA was removed with a stream of nitrogen. The dried residue was subjected to C18 cartridge (0.5 g) chromatography. For this, the residue was dissolved in NaCl solution (5 M, 0.5 mL) and applied onto the pre-equilibrated cartridge. The cartridge was then washed with NaCl solution (1 M, 2 mL) and water (8 mL, adjusted to pH 4.5 with 1 M HCl), the target compound was eluted with 20% acetonitrile in water (pH 4.5), and pure fractions were pooled and lyophilized to dryness. The 2'/3'-aminoacylated mononucleotide was quantified by UV absorption. Yield: 77% (192 nmol).

Data for 2'/3'-aminoacylated mononucleotides.

Table S2. Yields of Boc-protected 2'/3'-aminoacylated mononucleotides.

$(B)_n$-Aa-Boc	yield (%)	$[M-H]^-$ m/z calc.	$[M-H]^-$ m/z found (ESI MS)
A-Gly-Boc	21	503.130	503.131
A-Val-Boc	24	545.473	545.473
A-Leu-Boc	16	559.188	559.189
C-Gly-Boc	10	479.120	479.120
G-Ala-Boc	13	533.146	533.145
U-Leu-Boc	17	536.170	536.170
G-Val-Boc	19	561.178	561.178

Table S3. Yields of 2'/3'-aminoacylated mononucleotides with free amino group after Boc-deprotection.

$(B)_n$-Aa	yield (%)	$[M-H]^-$ m/z calc.	$[M-H]^-$ m/z found (ESI MS)
A-Gly	77	403.082	403.082
A-Val	99	445.356	445.355
A-Leu	74	449.137	449.137
C-Gly	67	379.066	379.067
G-Ala	73	433.091	433.092
U-Leu	62	436.111	436.113
G-Val	73	461.353	461.354

Data for 2'/3'-aminoacylated dinucleotides.

Table S4. Yields of Boc-protected 2'/3'-aminoacylated dinucleotides.

$(B)_n$-Aa-Boc	yield (%)	$[M-H]^-$ m/z calc.	$[M-H]^-$ m/z found (MALDI-TOF MS)
GA-Gly-Boc	25	768.2	767.1
GA-Val-Boc	39	810.2	810.4
GA-Leu-Boc	14	824.3	823.2
GA-Met-Boc	23	842.2	841.7
CC-Gly-Boc	13	704.2	703.5
GU-Leu-Boc	20	801.3	800.8
CU-Met-Boc	18	797.2	797.6

Table S5. Yields of 2'/3'-aminoacylated dinucleotides with free amino group after Boc-deprotection.

$(B)_n$-Aa	yield (%)	$[M-H]^-$ m/z calc.	$[M-H]^-$ m/z found (MALDI-TOF MS)
GA-Gly	66	668.2	667.5
GA-Val	84	710.2	710.6
GA-Leu	44	724.2	723.4
GA-Met	50	742.1	741.9
CC-Gly	49	604.1	603.3
GU-Leu	85	701.2	700.7
CU-Met	87	697.1	697.9

Data for 2'/3'-aminoacylated tri- and tetranucleotides.

Table S6. Yields of Boc-protected 2'/3'-aminoacylated tri- and tetranucleotides.

$(B)_n$-Aa-Boc	yield (%)	$[M\text{-}H]^-$ m/z calc.	$[M\text{-}H]^-$ m/z found (MALDI-TOF MS)
GGA-Gly-Boc	20	1112.3	1111.9
GGA-Val-Boc	16	1154.3	1154.7
GGA-Leu-Boc	7	1168.3	1169.0
GGA-Met-Boc	22	1186.3	1187.2
AGG-Val-Boc	10	1154.3	1154.5
AAC-Met-Boc	10	1130.3	1130.7
CAA-Val-Boc	12	1098.3	1098.5
ACAA-Met-Boc	14	1425.0	1426.4
GGGA-Gly-Boc	12	1457.0	1457.6

Table S7. Yields of 2'/3'-aminoacylated tri- and tetranucleotides with free amino group after Boc-deprotection.

$(B)_n$-Aa	yield (%)	$[M\text{-}H]^-$ m/z calc.	$[M\text{-}H]^-$ m/z found (MALDI-TOF MS)
GGA-Gly	42	1012.2	1011.5
GGA-Val	45	1054.3	1054.3
GGA-Leu	84	1068.3	1068.5
GGA-Met	40	1086.2	1085.9
AGG-Val	40	1054.3	1053.9
AAC-Met	62	1030.2	1030.1
CAA-Val	74	998.2	997.4
ACAA-Met	58	1324.9	1326.0
GGGA-Gly	65	1356.9	1356.6

Ethyl phosphate

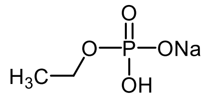

A sample of ethyl dichloro phosphate (3.26 g, 20 mmol) was suspended in water (10 mL) and hydrolyzed by stirring for 16 h. The resulting solution was concentrated *in vacuo* to remove all volatiles, including hydrogen chloride. The residue was then dissolved in water (10 mL), and the pH was adjusted to a value of 5.0 by addition of NaOH solution (2 M). Precipitation with ethanol (40 mL), collection by filtration and drying *in vacuo* then gave ethyl phosphate monosodium salt as colorless solid. Yield: 95 % (2.81 g, 19 mmol). ^{1}H NMR (400 MHz, D_2O, 298 K): $\delta = 3.81$ (dq, $J = 7.1, 7.1$, 2H), 1.20 (t, $J = 7.1$, 3H), ^{31}P NMR (162 MHz, D_2O, 298 K): $\delta = 2.00$.

Glycinyl Adenosine 5'-Monophosphate (Gly-A)

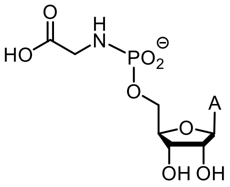

The synthesis of Gly-A is a modification of a literature-known procedure.[S6] Samples of AMP (347 mg, 1.0 mmol, 1 eq.), glycine methyl ester hydrochloride (251 mg, 2.0 mmol, 2 eq.), and EDC hydrochloride (394 mg, 4.0 mmol, 4 eq.) were dissolved in NaOH solution (1 M, 1 mL). The pH was adjusted to a value of 5.5-6.0 and re-adjusted during the course of the reaction by addition of additional NaOH solution. After 1 h, full conversion to the desired phosphoramidate was observed by ^{31}P-NMR spectroscopy. Then, NaCl solution (5 M, 1 mL) was added, and the reaction mixture subjected to automatic chromatography, eluting with a water/acetonitrile gradient. Product-containing fractions were lyophilized to dryness to obtain Gly-A methyl ester with protonated EDU as counterion. The methyl ester was dissolved in water (500 µL) and converted to its sodium form by precipitation, induced by addition to a solution of sodium perchlorate (0.1 M, 40 mL) in acetone/diethyl ether (1:1). Saponification of the methyl ester was achieved by hydrolysis in a sodium hydroxide solution. For this, the methyl ester was dissolved in H_2O/D_2O (1 mL, 9:1) and kept at pH 11-12 by repeated addition of NaOH solution (2 M). When the ^{31}P-spectrum of an analytical sample indicated full conversion (1-2 h), the product was precipitated by addition of ethanol (40 mL) and collected by filtration. Residual

solvents were evaporated *in vacuo*. The target molecule Gly-A was obtained as colorless solid. Yield: 66% (229 mg, 0.66 mmol). The analytical data were in accordance with the literature.[S6]

^{13}C-Labeled Glycine Methyl Ester

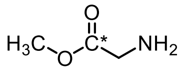

The target compound, ^{13}C-labeled glycine methyl ester, was prepared analogously to a literature-known procedure.[S7] Briefly, a sample of ^{13}C-labeled glycine (75.2 mg, 1.0 mmol, 1 eq.) was suspended in methanol and cooled to 0 °C. Trimethylchlorosilane (217 mg, 0.25 mL, 2.0 mmol, 2. eq.) was added and the mixture was allowed to warm to room temperature. The reaction was monitored via ^{13}C-NMR spectroscopy of aliquots. After 38 h full conversion to the ester was indicated and the product was obtained in the hydrochloride form in quantitative yield by removal of all volatiles under reduced pressure. The analytical data were in accordance with the literature.[S7] ^{13}C NMR (101 MHz, D_2O, 298 K): $\delta = 168.7$.

^{13}C-Labeled Gly-A

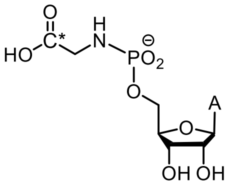

The ^{13}C-labeled Gly-A was prepared analogously to Gly-A using ^{13}C-labeled glycine methyl ester. A sample of AMP (104 mg, 0.3 mmol, 1 eq.), ^{13}C-labeled glycine methyl ester (45 mg, 0.36 mmol, 1.2 eq) and EDC (118 mg, 0.6 mmol, 2 eq.) were reacted at pH 5.5-6.0, followed by precipitation and saponification, to obtain ^{13}C-labeled Gly-A in 47 % (49 mg, 0.14 mmol) yield. The analytical data were in accordance with the literature.[S6] ^{13}C NMR (101 MHz, D_2O, 298 K): $\delta = 179.0$.

^{13}C-Labeled MeO-GlyGly-A

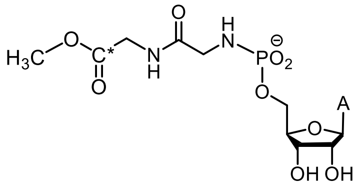

A sample of Gly-A (45 mg, 0.10 mmol, 1 eq.), ^{13}C-labeled glycine methyl ester (25 mg, 0.20 mmol, 2 eq.), ethyl phosphate (124 mg, 1.0 mmol, 10 eq.) and MES (43 mg, 0.2 mmol, 2 eq.) were dissolved in a mixture of water (800 µL) and D_2O (100 µL). The pH was adjusted to a value of 6.0 and the solution was cooled to 0 °C. EDC (394 mg, 2.00 mmol, 20 eq.) was dissolved in water (350 µL), cooled to 0 °C and added to the reaction mixture. After 38 h, ^{31}P-NMR spectroscopy indicated full conversion of Gly-A to the desired dipeptide. An aliquot of NaCl solution (5 M, 1 mL) was added and the reaction mixture was subjected to automatic chromatography, eluting with a water/acetonitrile gradient. Product-containing fractions were lyophilized to dryness to obtain MeO-GlyGly-A with protonated EDU as counterion. The compound was dissolved in water (500 µL) and converted to its sodium form by precipitation with a solution of sodium perchlorate (0.1 M, 40 mL) in acetone/diethyl ether (1:1). The product was collected by centrifugation and dried under reduced pressure. Yield: 84% (38 mg, 0.084 mmol). ^{1}H NMR (400 MHz, D_2O, 298 K): δ = 8.38 (s, 1H), 8.18 (s, 1H), 6.04 (d, J = 5.5, 1H), 4.42 (t, J = 4.6, 1H), 4.31-4.26 (m, 1H), 4.06-3.95 (m, 2H), 3.84 (d, J = 6.2, 2H), 3.61 (d, J = 3.9, 3H), 3.43 (d, J = 11.0, 2H), ^{13}C NMR (101 MHz, D_2O, 298 K): δ = 171.9, ^{31}P NMR (162 MHz, D_2O, 298 K): δ = 7.67.

Mixed Anhydride (Et-p-Gly-A)

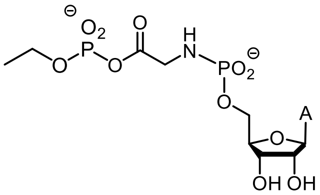

A sample of Gly-A (80 mg, 0.20 mmol, 1 eq.), MES (87 mg, 0.40 mmol, 2 eq.) and ethyl phosphate (124 mg, 1.00 mmol, 5 eq.) were dissolved in water (1 mL). The pH was adjusted to a value of 6.0 and the solution was cooled to 0 °C. A Sample of EDC (394 mg, 2.00 mmol, 10 eq.) was dissolved in water (350 µL), cooled to 0 °C and added to the reaction mixture. The mixture was vortexed and kept at 4 °C. After 16 h, when ^{31}P NMR indicated full conversion to

the mixed anhydride, NaCl solution (5 M, 1 mL) was added and the reaction mixture subjected to automatic chromatography, eluting with a water (pH = 6.0)/acetonitrile gradient. Product-containing fractions were lyophilized to dryness to obtain the mixed anhydride Et-p-Gly-A with EDU as its counterion. The compound was dissolved in water (500 µL) and converted to its sodium form by precipitation using a solution of sodium perchlorate (0.1 M, 40 mL) in acetone/diethyl ether (1:1). It was collected by centrifugation and dried under reduced pressure. Mixed anhydride Et-p-Gly-A was obtained in its disodium form. Yield: 84% (58 mg, 0.168 mmol). As a solid, the compound is very sensitive to moisture and should be prepared as freshly as possible prior to its use. ^{1}H NMR (400 MHz, D_2O, 298 K): δ = 8.46 (s, 1H), 8.20 (s, 1H), 6.06 (d, J = 5.9, 1H), 4.41 (t, J = 5.1, 3.6, 1H), 4.32-4.28 (m, 1H), 4.02-3.98 (m, 2H), 3.83 (dq, J = 8.1, 7.2, 2H), 3.63 (d, J = 11.1, 2H), 1.09 (t, J = 7.2, 3H), ^{13}C NMR (126 MHz, D_2O, 277 K): δ = 169.9, 155.0, 152.3, 148.6, 139.4, 118.1, 86.4, 83.9, 74.0, 71.7, 70.3, 63.5, 63.3, 62.1, 43.4, 29.9, 15.1, ^{31}P NMR (162 MHz, D_2O, 298 K): δ = 7.23, -7.27, HRMS (ESI): m/z, calculated for $C_{15}H_{23}N_5O_{11}P_2$, [M-H]$^-$: 511.0875, found: 511.0747.

3. Mass and NMR Spectra

3.1 Mass Spectra

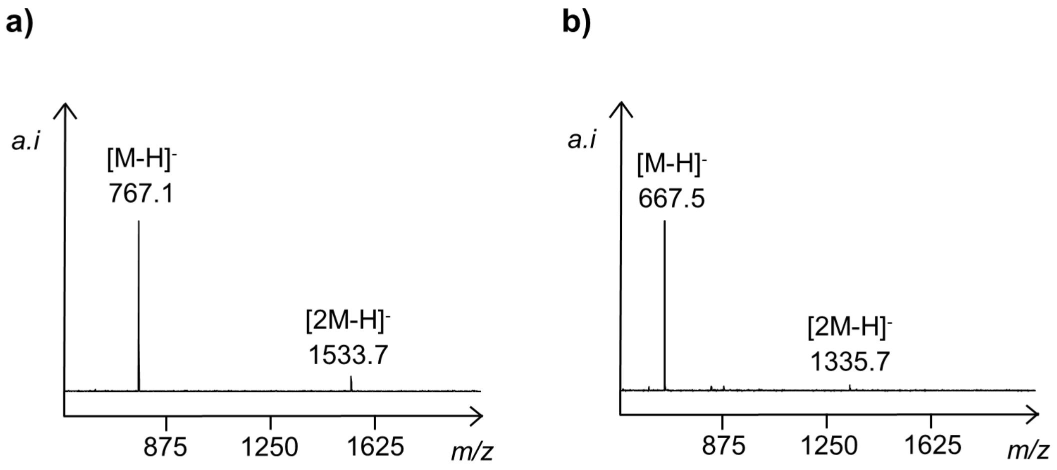

Figure S1. MALDI-TOF mass spectra of a) GA-Gly-Boc and b) GA-Gly.

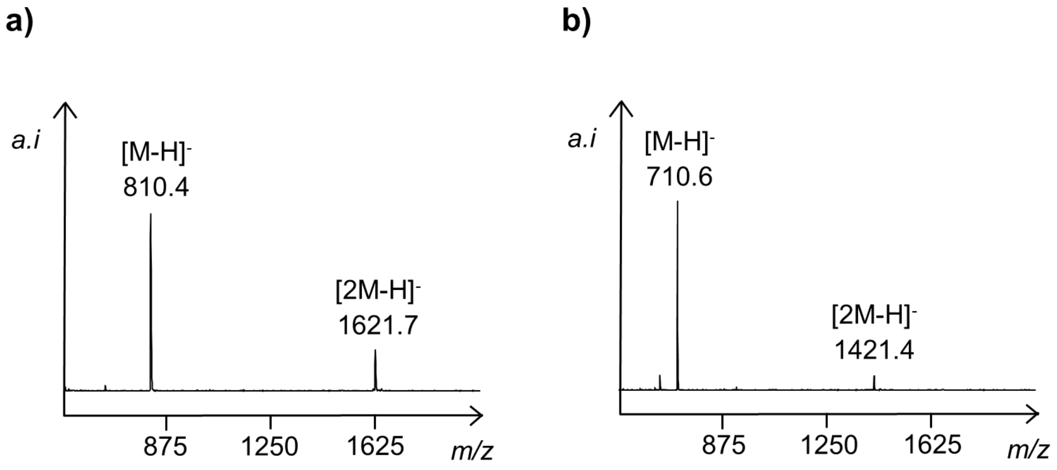

Figure S2. MALDI-TOF mass spectra of a) GA-Val-Boc and b) GA-Val.

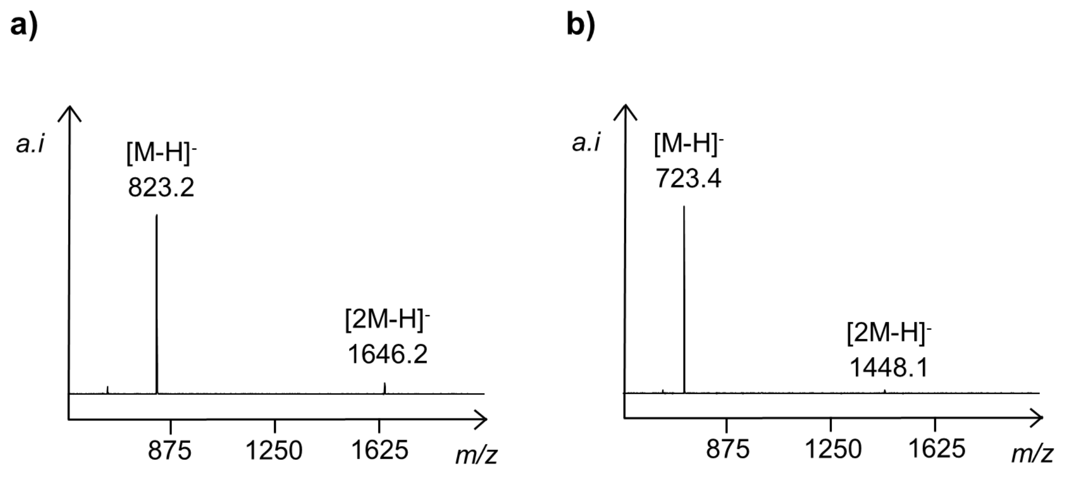

Figure S3. MALDI-TOF mass spectra of a) GA-Leu-Boc and b) GA-Leu.

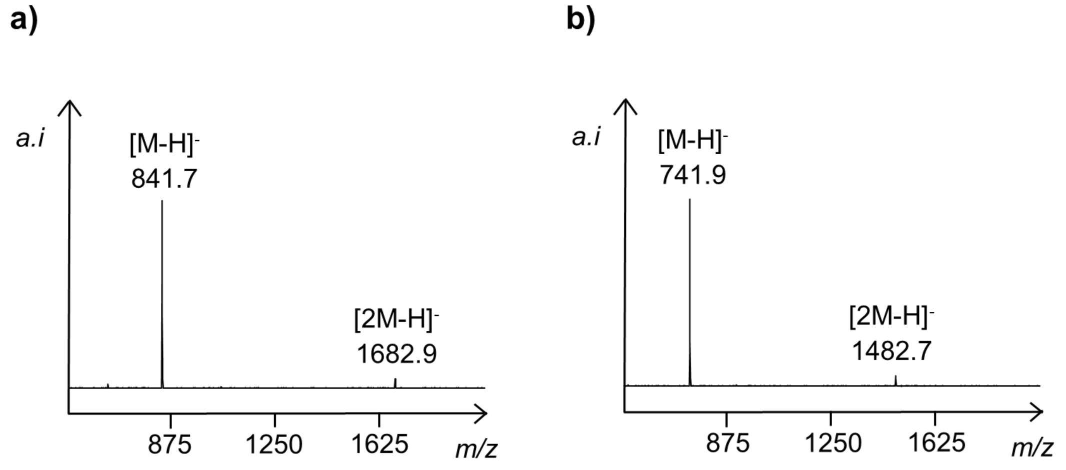

Figure S4. MALDI-TOF mass spectra of a) GA-Met-Boc and b) GA-Met.

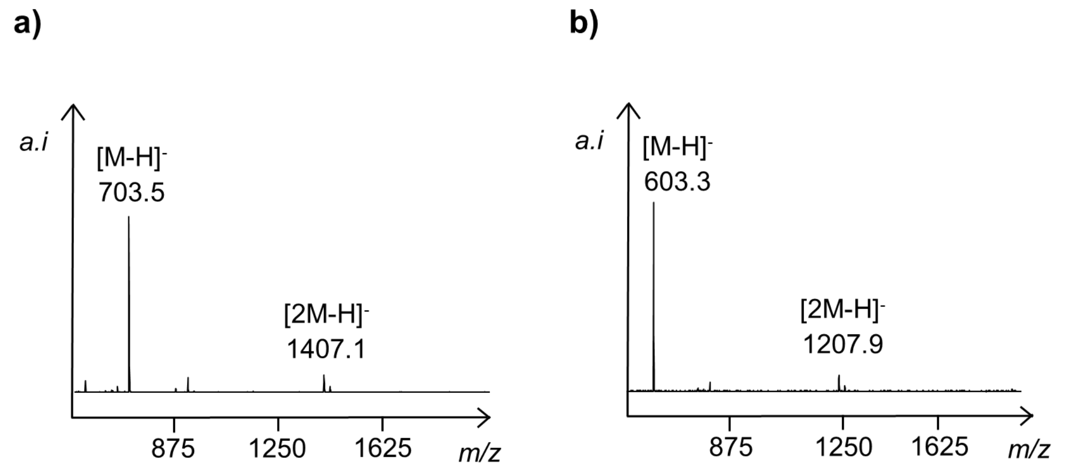

Figure S5. MALDI-TOF mass spectra of a) CC-Gly-Boc and b) CC-Gly.

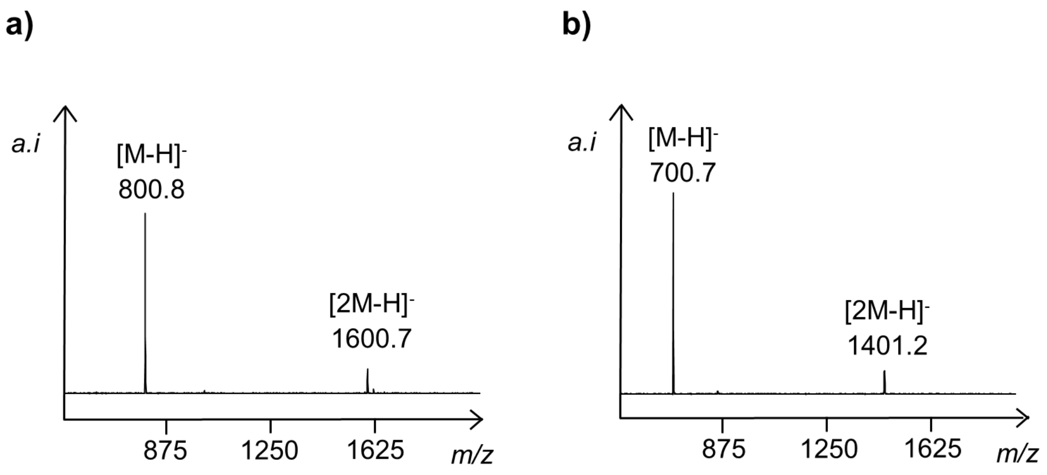

Figure S6. MALDI-TOF mass spectra of a) GU-Leu-Boc and b) GU-Leu.

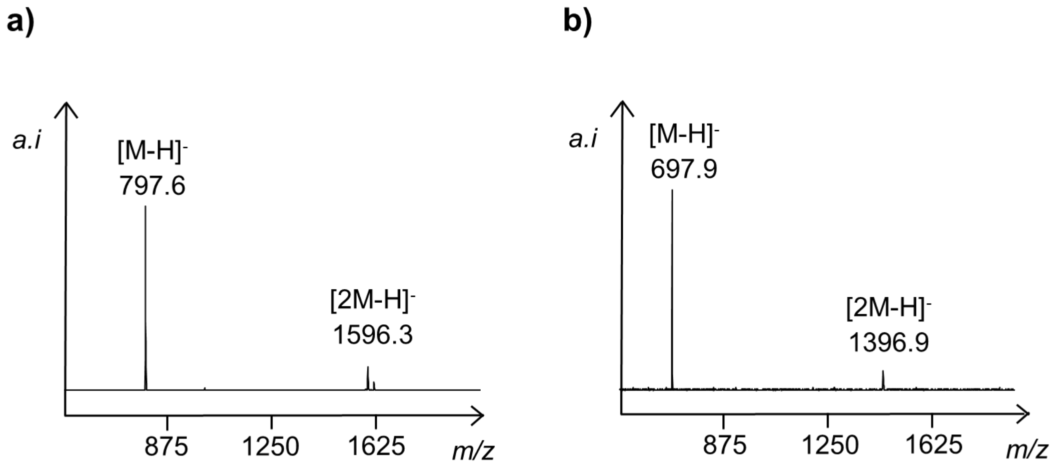

Figure S7. MALDI-TOF mass spectra of a) CU-Met-Boc and b) CU-Met.

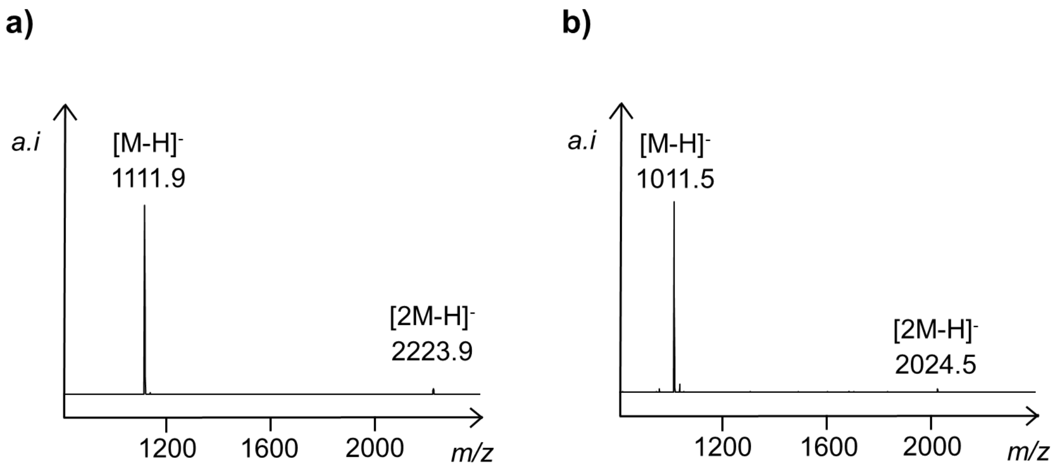

Figure S8. MALDI-TOF mass spectra of a) GGA-Gly-Boc and b) GGA-Gly.

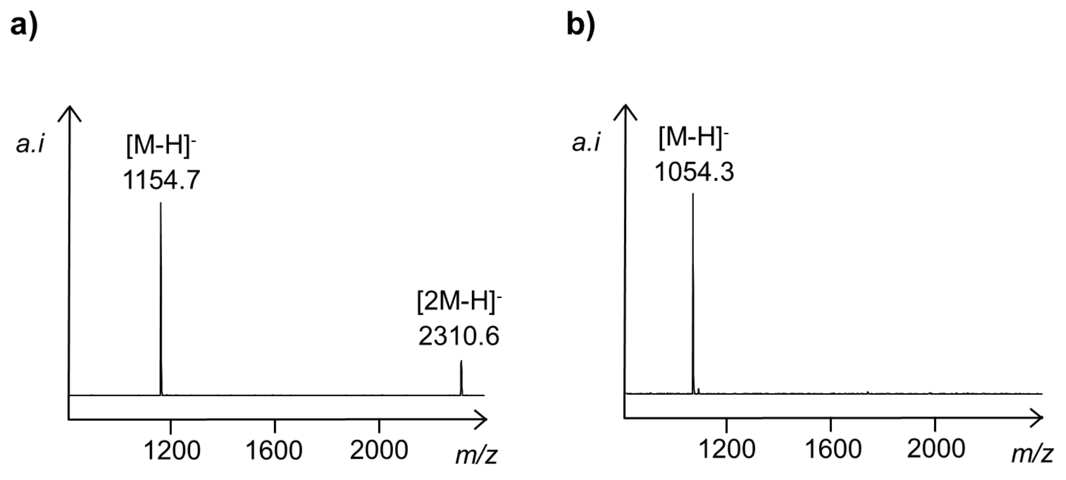

Figure S9. MALDI-TOF mass spectra of a) GGA-Val-Boc and b) GGA-Val.

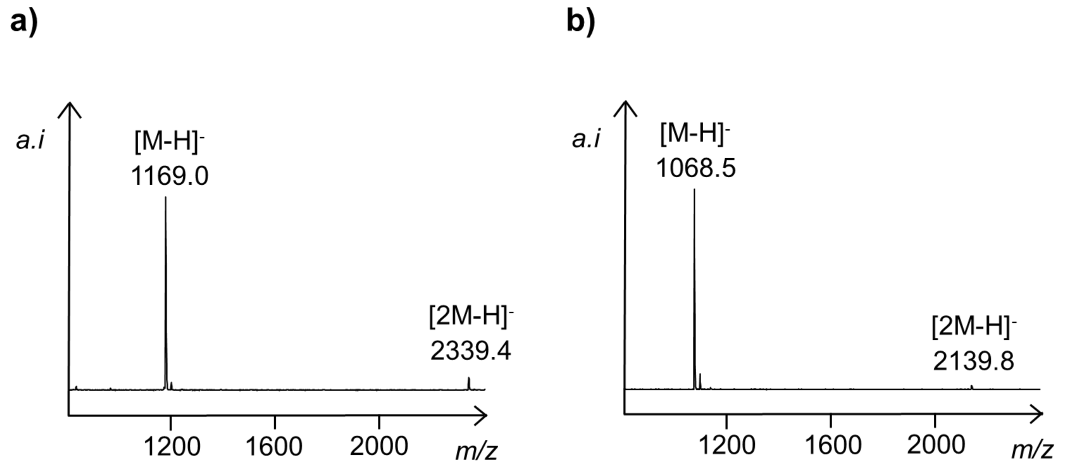

Figure S10. MALDI-TOF mass spectra of a) GGA-Leu-Boc and b) GGA-Leu.

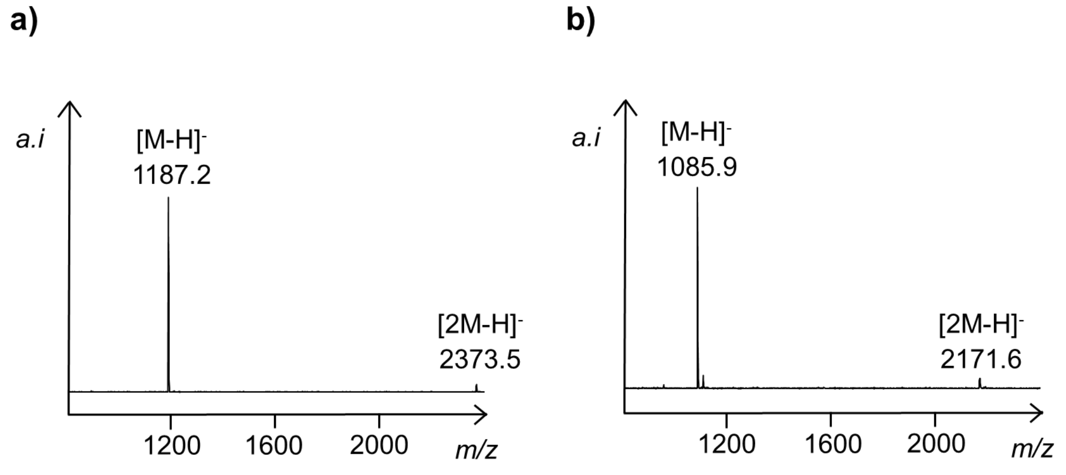

Figure S11. MALDI-TOF mass spectra of a) GGA-Met-Boc and b) GGA-Met.

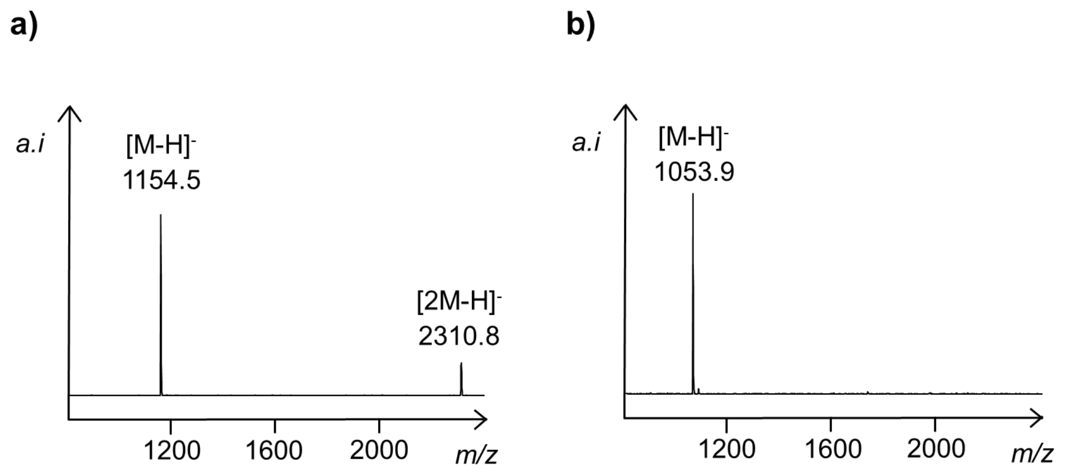

Figure S12. MALDI-TOF mass spectra of a) AGG-Val-Boc and b) AGG-Val.

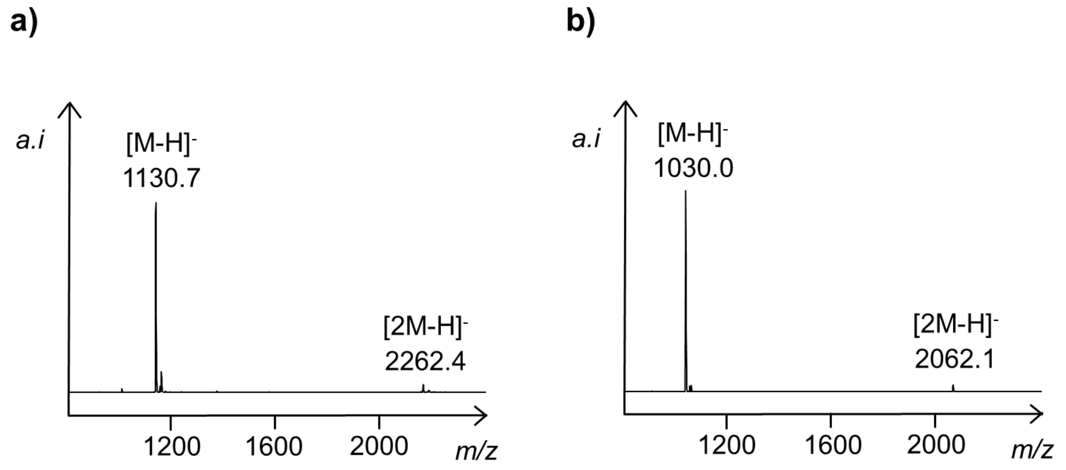

Figure S13. MALDI-TOF mass spectra of a) AAC-Met-Boc and b) AAC-Met.

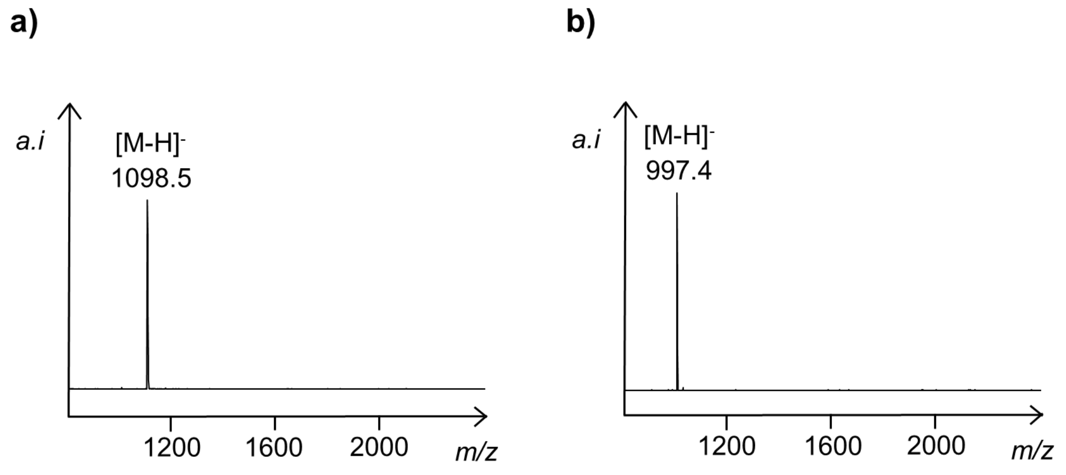

Figure S14. MALDI-TOF mass spectra of a) CAA-Val-Boc and b) CAA-Val.

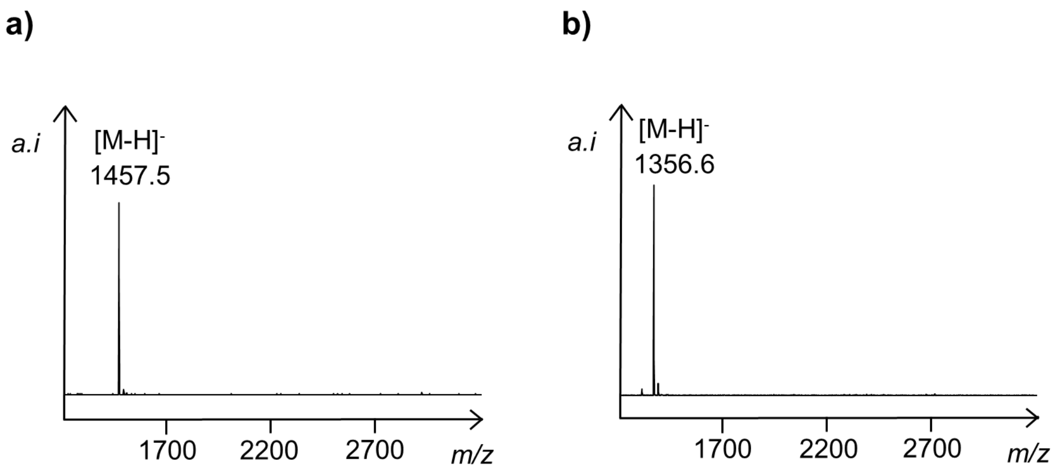

Figure S15. MALDI-TOF mass spectra of a) ACAA-Met-Boc and b) ACAA-Met.

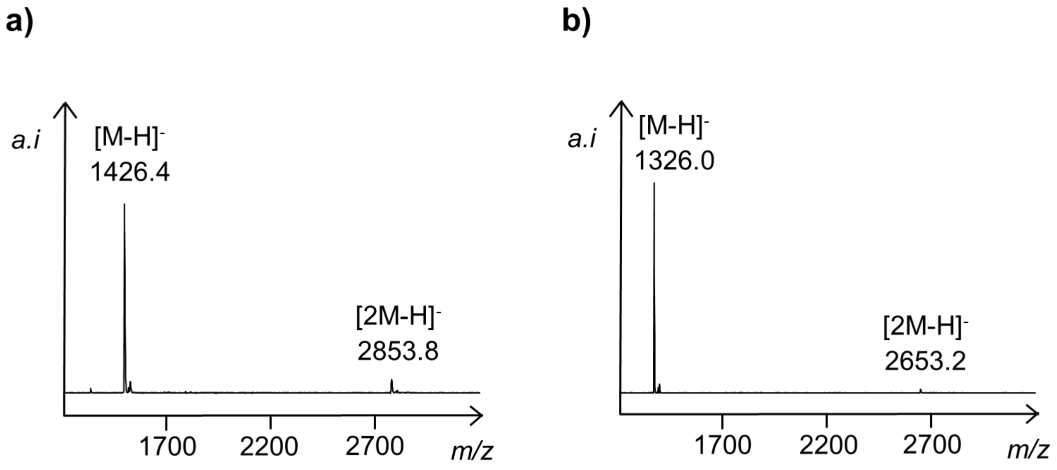

Figure S16. MALDI-TOF mass spectra of a) GGGA-Gly-Boc and b) GGGA-Gly.

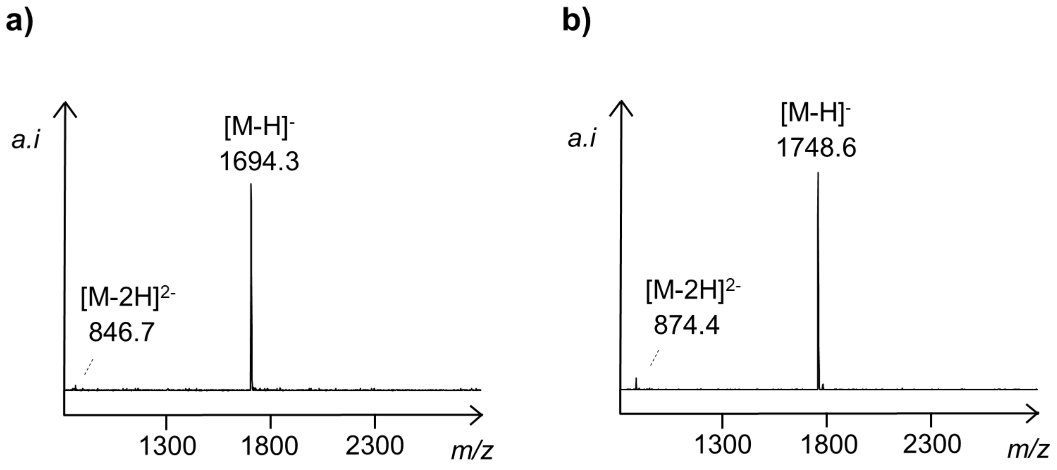

Figure S17. MALDI-TOF mass spectra of a) 5'-pAAGAG-3' and b) Gly-pAAGAG-3'.

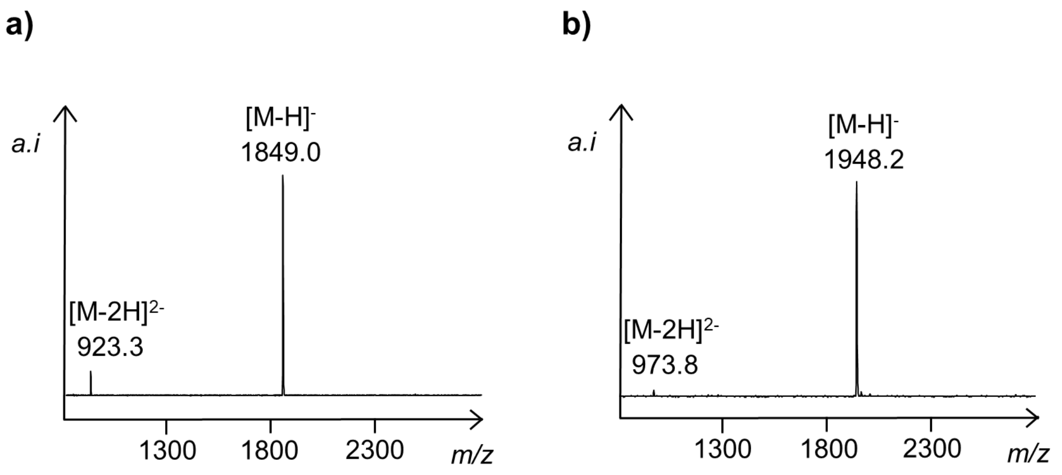

Figure S18. MALDI-TOF mass spectra of a) ValGly-pAAGAG-3' and b) Val_2Gly-pAAGAG-3'.

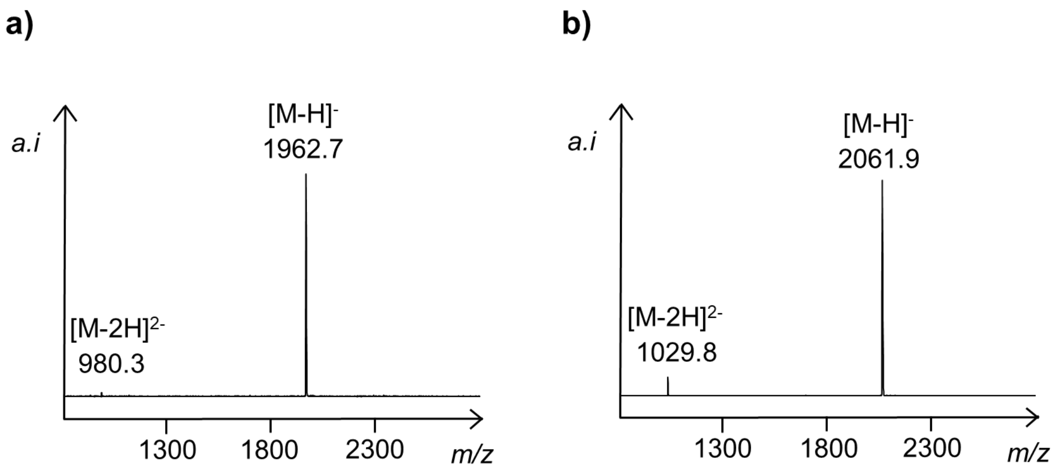

Figure S19. MALDI-TOF mass spectra of a) $ValGly_3$-pAAGAG-3' and b) Val_2Gly_3-pAAGAG-3'.

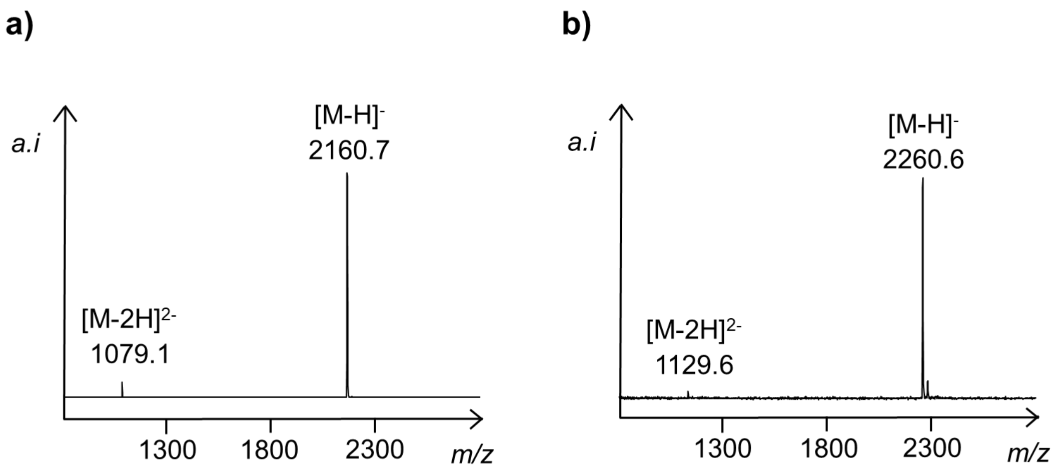

Figure S20. MALDI-TOF mass spectra of a) Val_3Gly_3-pAAGAG-3' and b) Val_4Gly_3-pAAGAG-3'.

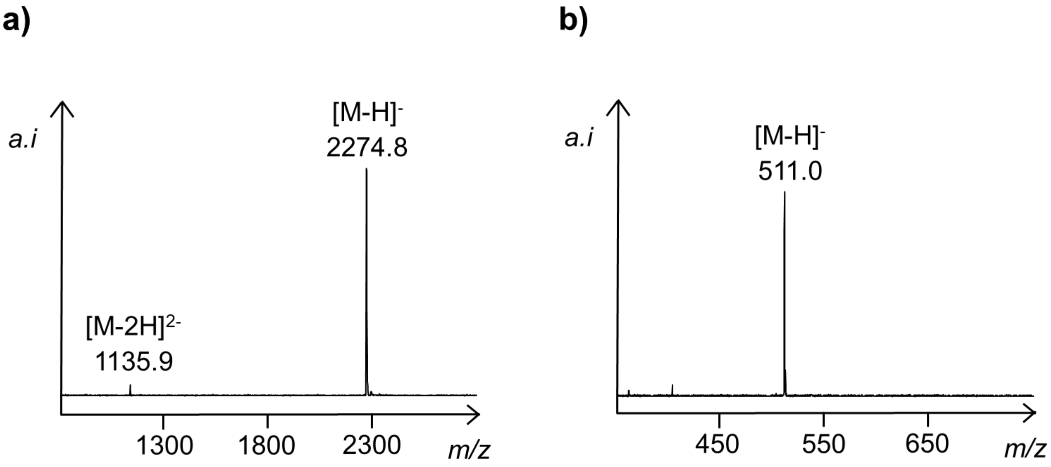

Figure S21. MALDI-TOF mass spectra of a) LeuVal$_3$Gly$_3$-pAAGAG-3' and b) Et-p-Gly-A.

3.2 NMR Spectra

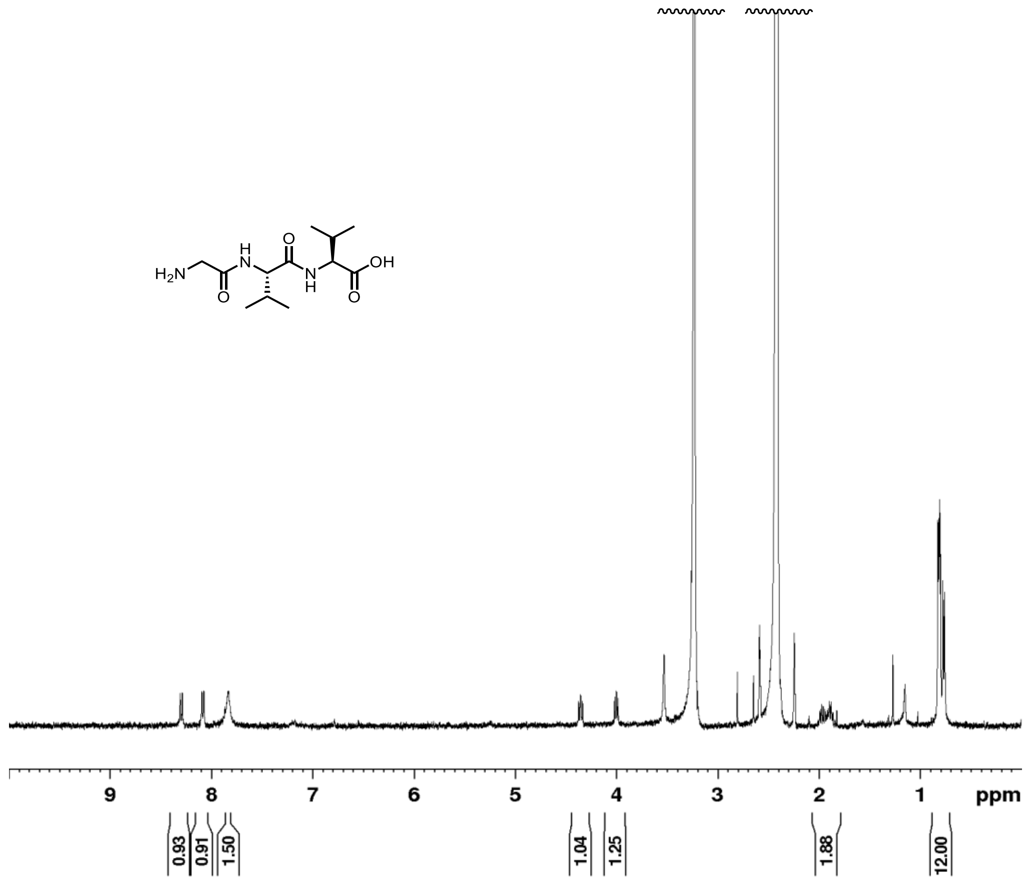

Figure S22. ¹H-NMR spectrum of H-GlyValVal-OH ($DMSO_{d6}$, 700 MHz, 298 K).

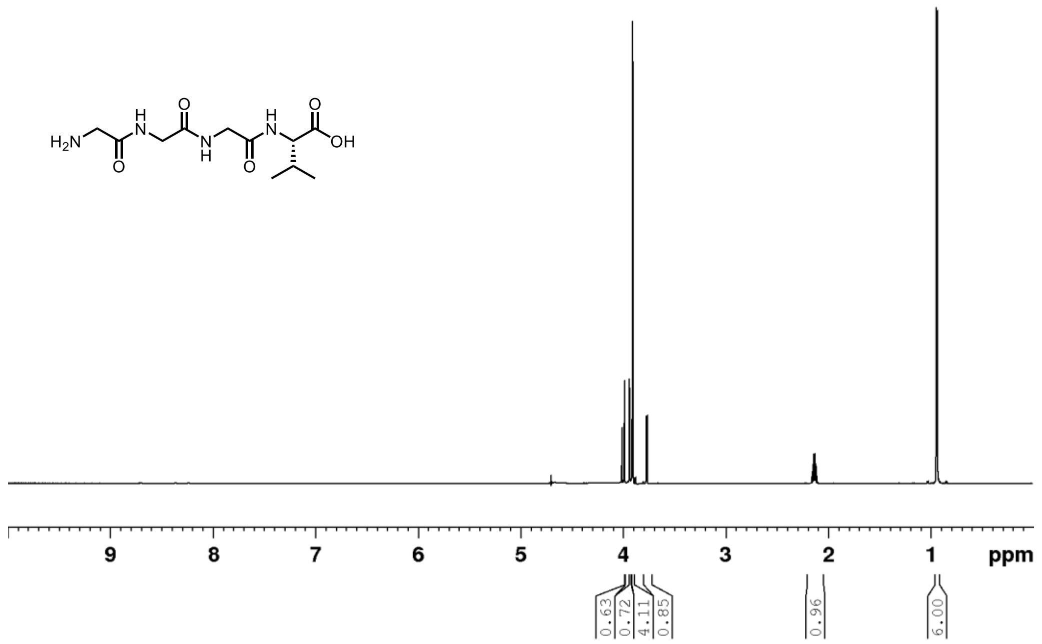

Figure S23. ¹H-NMR spectrum of H-GlyGlyGlyVal-OH (D_2O, 700 MHz, 298 K).

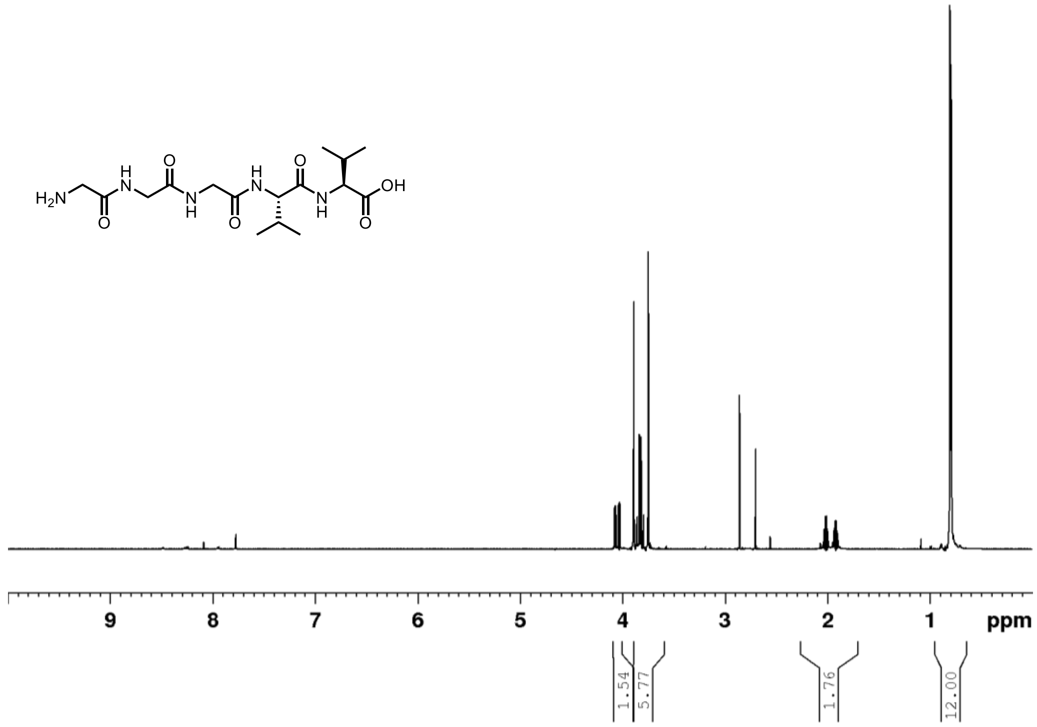

Figure S24. ^{1}H-NMR spectrum of H-GlyGlyGlyValVal-OH (D_2O, 700 MHz, 298 K).

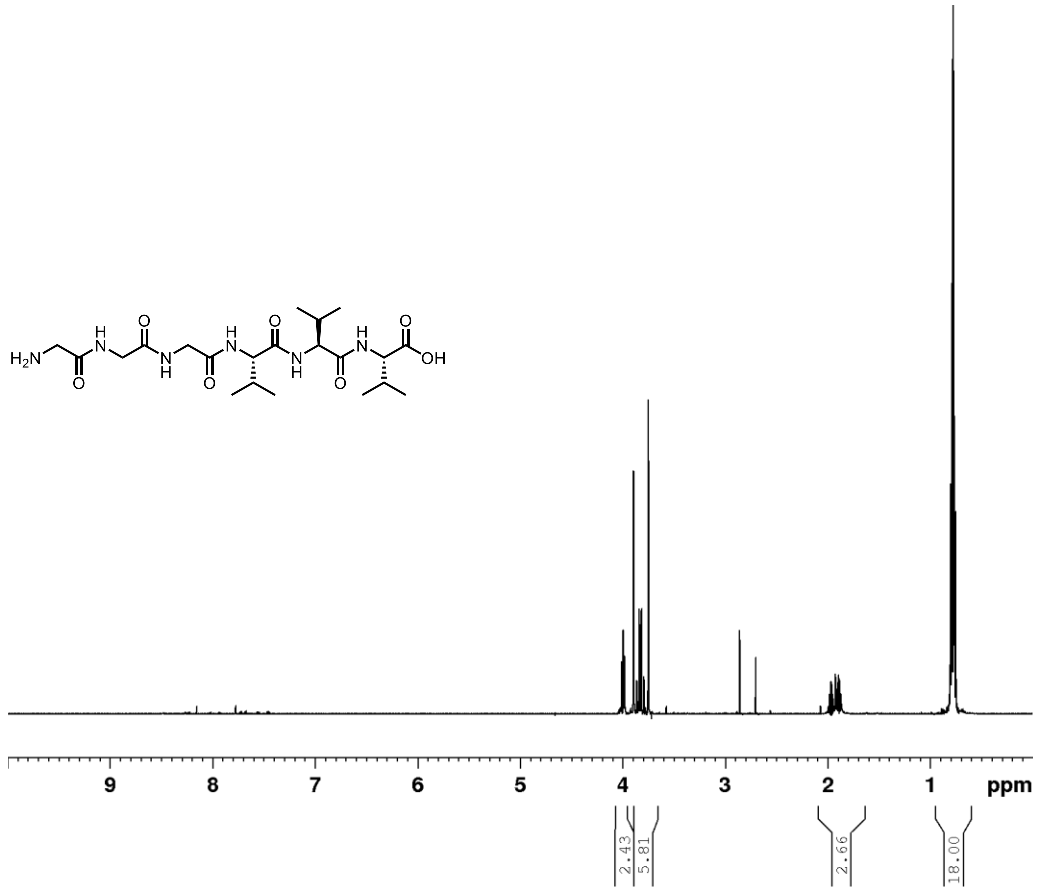

Figure S25. ^{1}H-NMR spectrum of H-GlyGlyGlyValValVal-OH (D_2O, 700 MHz, 298 K).

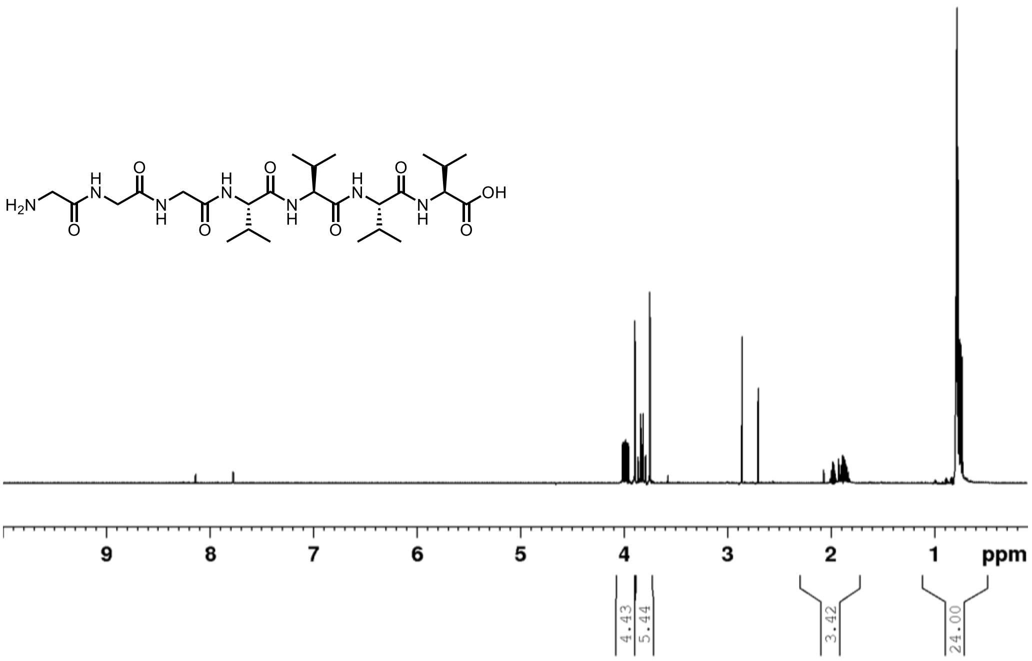

Figure S26. ^{1}H-NMR spectrum of H-GlyGlyGlyValValValVal-OH (D_2O, 700 MHz, 298 K).

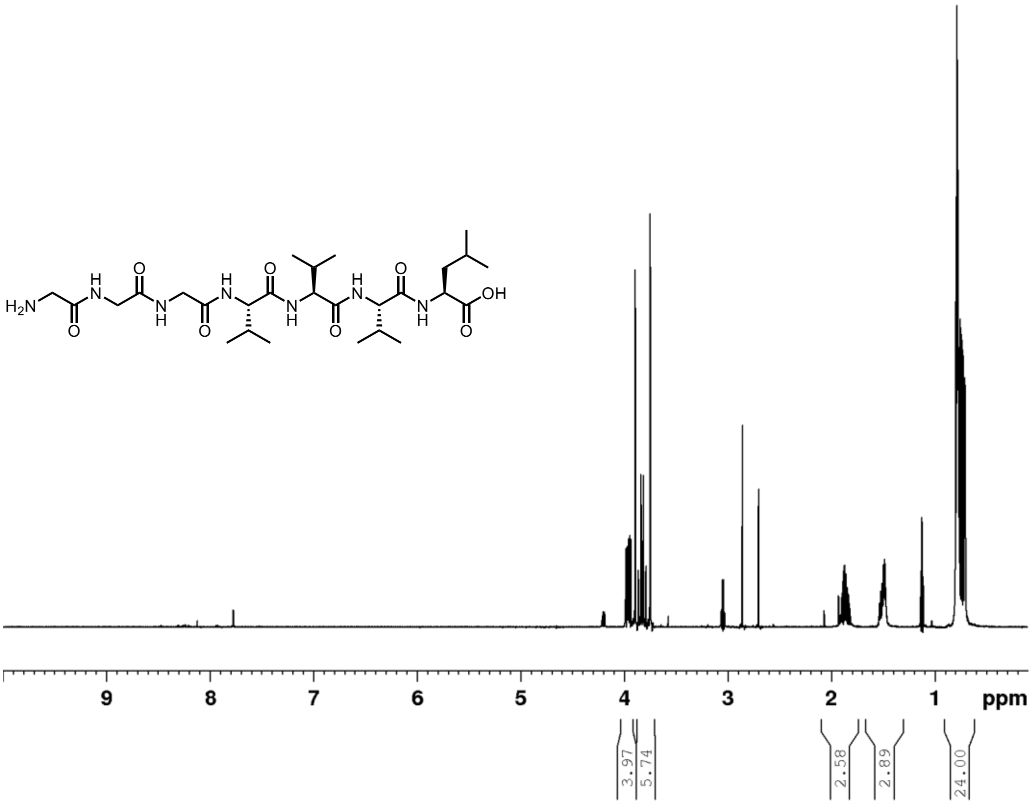

Figure S27. ^{1}H-NMR spectrum of H-GlyGlyGlyValValValLeu-OH (D_2O, 700 MHz, 298 K).

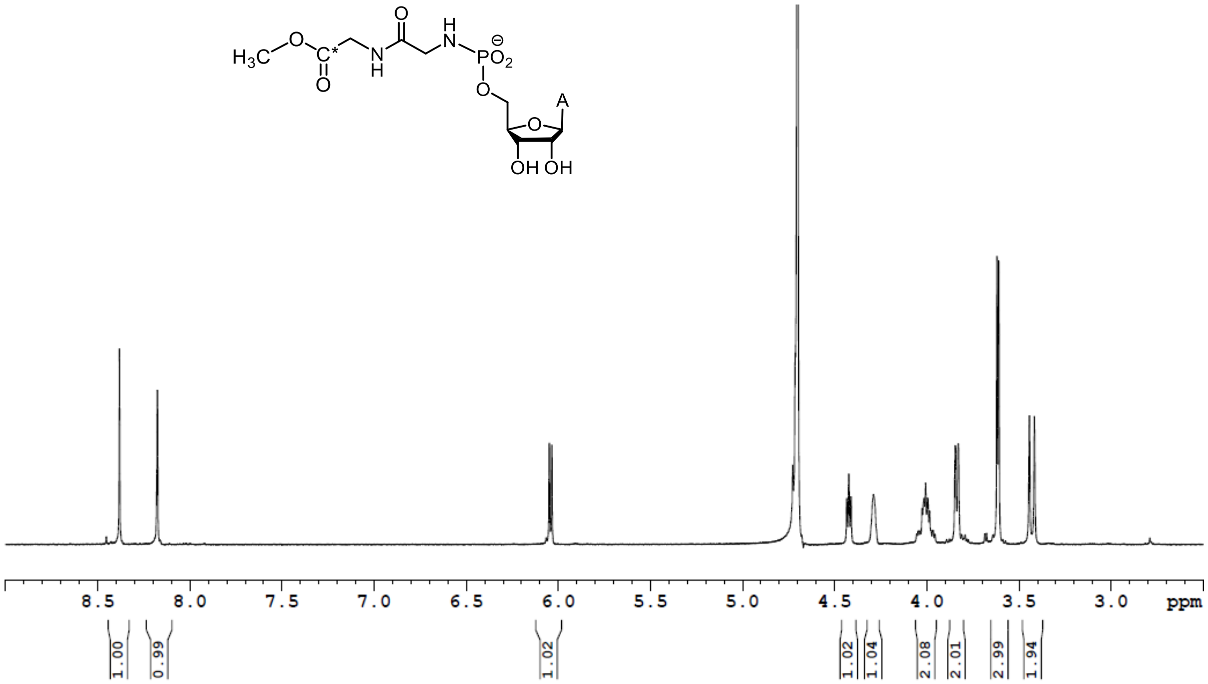

Figure S28. ^{1}H NMR spectrum of ^{13}C-labeled MeO-GlyGly-A (D_2O, 400 MHz, 298 K).

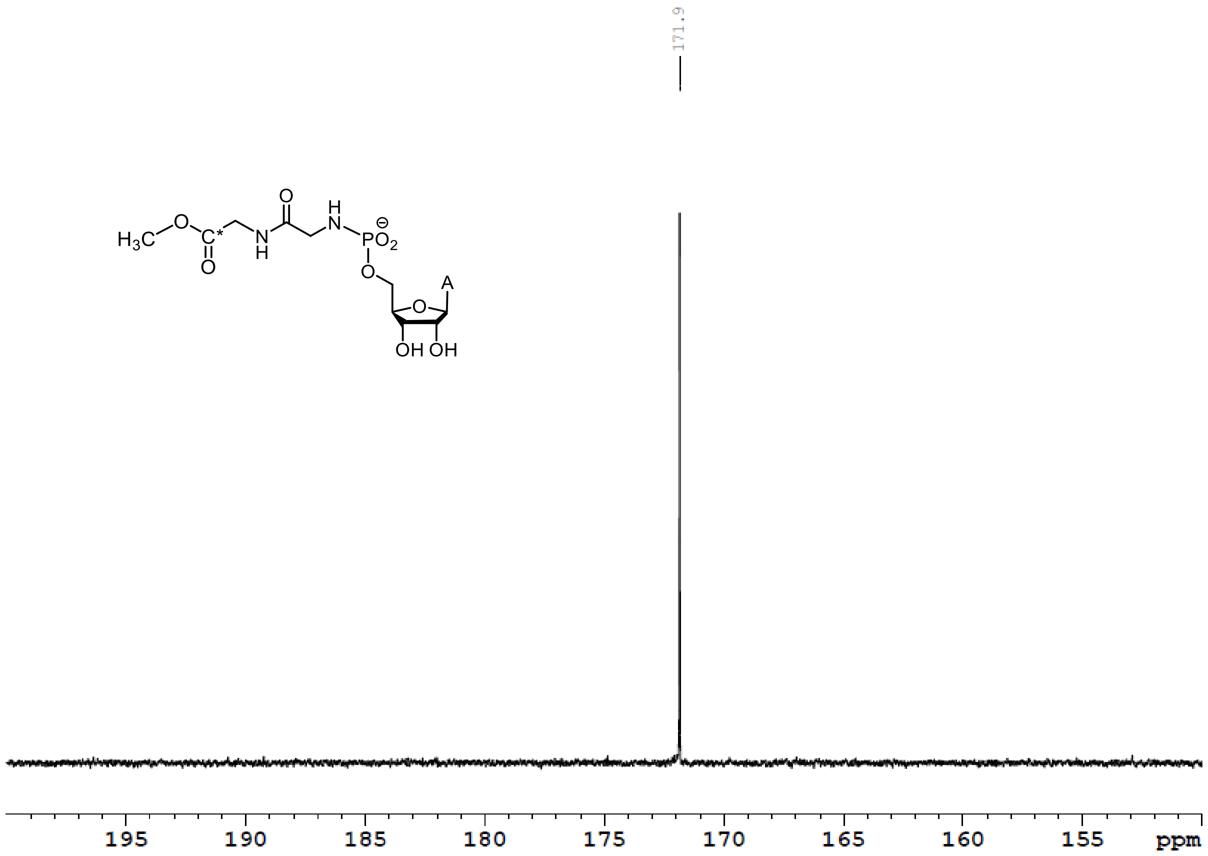

Figure S29. ^{13}C NMR spectrum of ^{13}C-labeled MeO-GlyGly-A (D_2O, 101 MHz, 298 K).

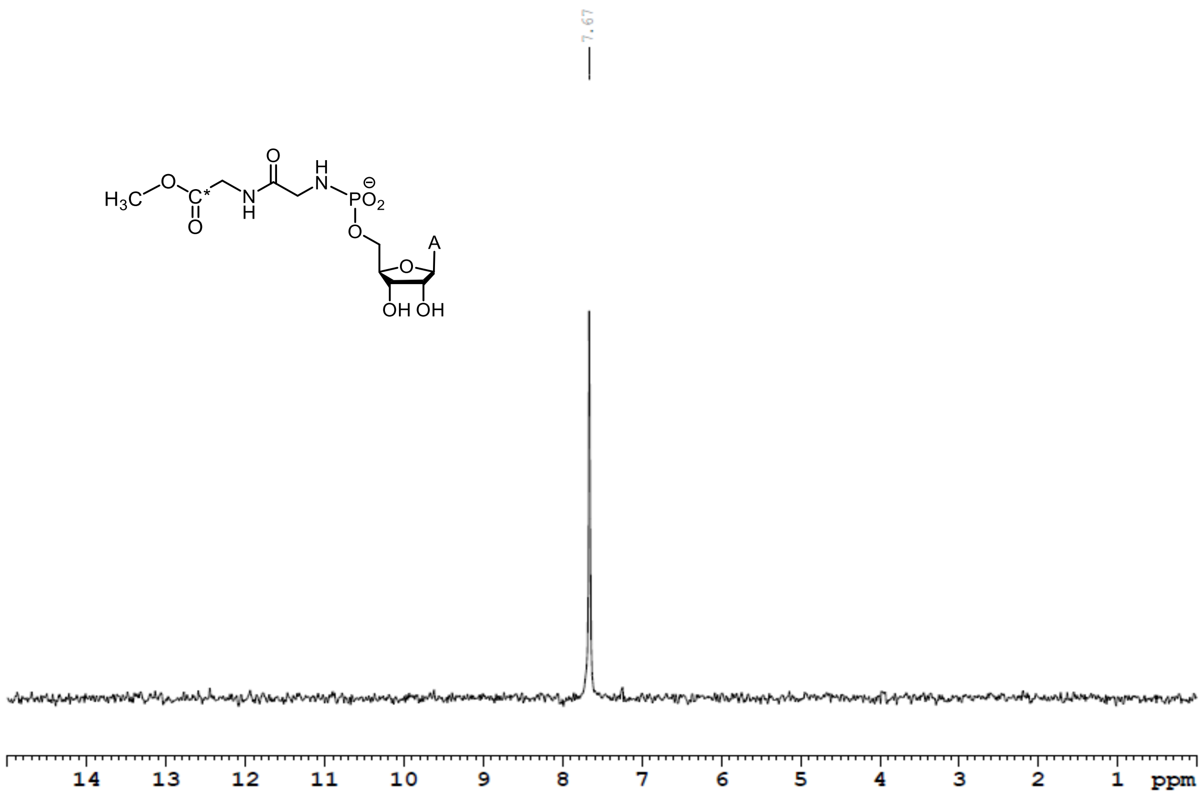

Figure S30. ^{31}P NMR spectrum of ^{13}C-labeled MeO-GlyGly-A (D_2O, 162 MHz, 298 K).

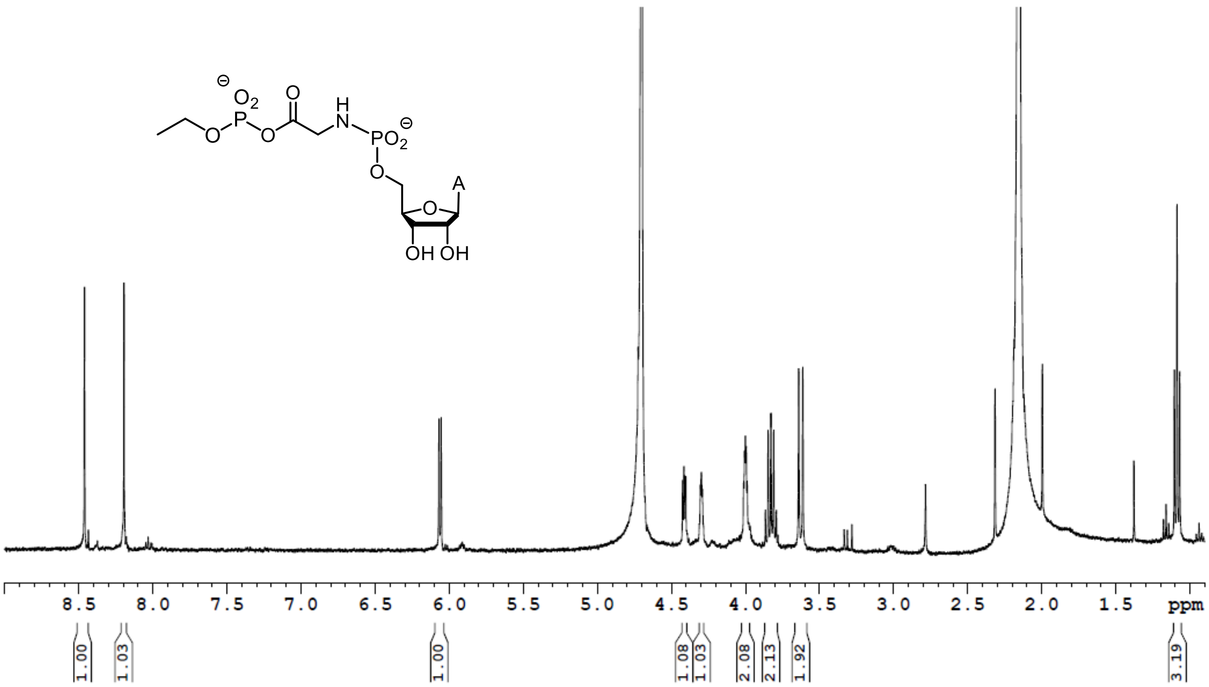

Figure S31. ^{1}H NMR spectrum of Et-p-Gly-A (D_2O, 400 MHz, 298 K).

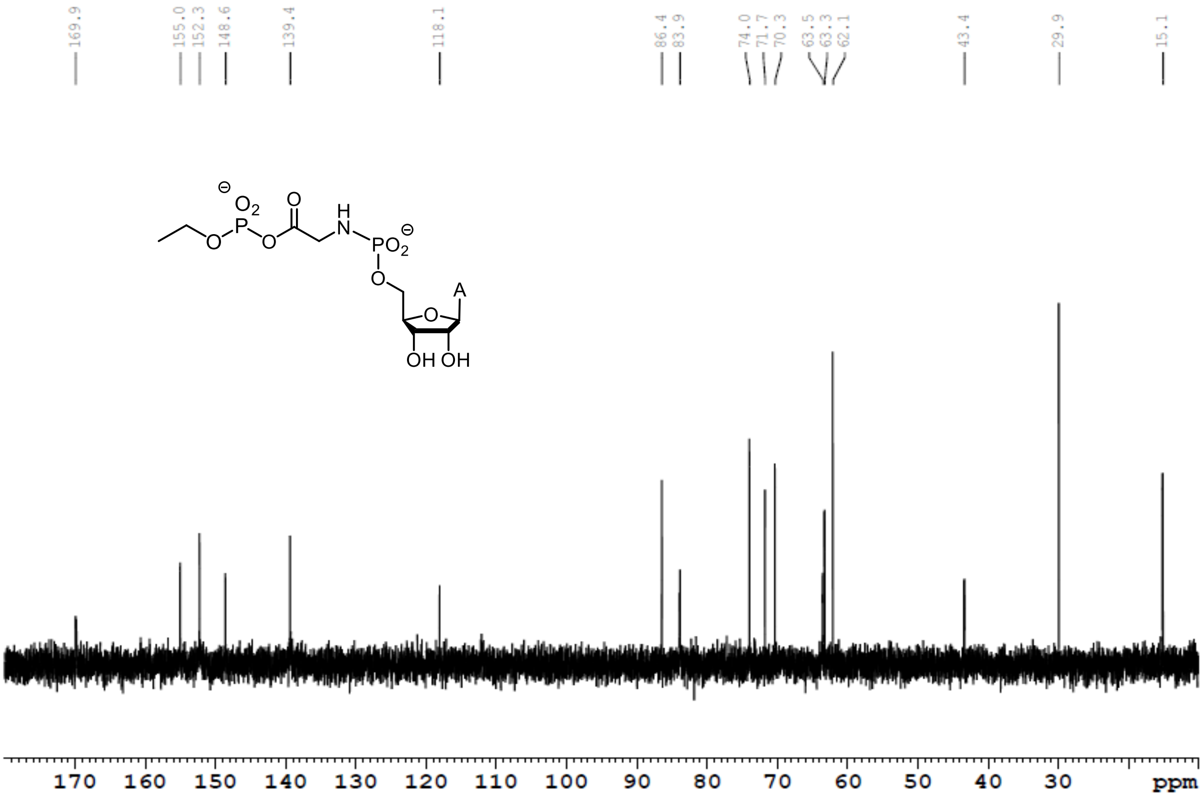

Figure S32. ^{13}C NMR spectrum of Et-p-Gly-A (D_2O, 126 MHz, 277 K).

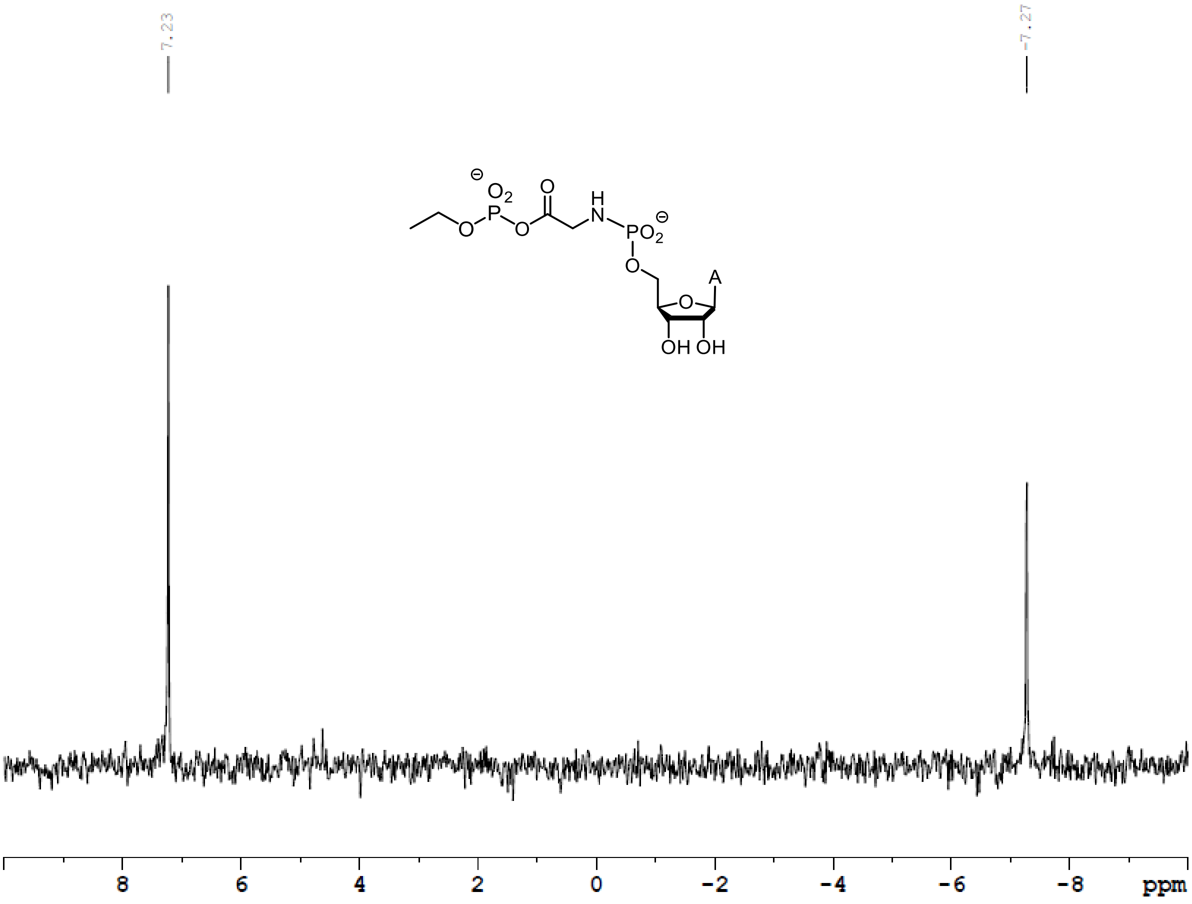

Figure S33. ^{31}P NMR spectrum of Et-p-Gly-A (D_2O, 162 MHz, 298 K).

4. UV-Melting Curve Experiments

UV-melting curve experiments were conducted as described in Chapter 1 to demonstrate triplex formation for the RNA strands involved in the translation assays. We varied the template strand to confirm that **1a** hybridizes with oligopurines pAAGAG and GGGA, resulting in a triplex that is stable at room temperature or below, while templates where the overhang is not capable of triplex formation (**1c** and **1d**), or is pruned (**1e**), do not (or to a much lesser extent), giving melting points (T_m's) and hyperchromicities well below that of the former complex. In a separate set of melting experiments, we varied the pH. Under the mildly acidic conditions of our assays (pH 6), triplex formation is favored, due to protonation of N3 of cytidine residues, while at neutral or basic pH values, triplexes should be weakened, again leading to significant drops in T_m and hyperchromicity.

Table S8. UV-melting points (T_m) of different templates, in the presence of primer (pAAGAG) and transfer (GGGA) strands in buffer at pH 6.0.[a,b]

Template		T_m / °C
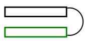	1a	43
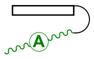	1c	32
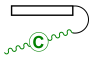	1d	31
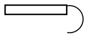	1e	23

[a] Melting points are the average of the extrema of the first derivative of two heating and two cooling curves at rates of 1 °C/min. Melting points thus obtained agreed to with ± 1 °C.

[b] Conditions: 1 µM template **1a-e**, 1 µM pAAGAG, 1 µM GGGA, 10 mM phosphate buffer pH 6.0, 1 M NaCl.

Table S9. UV-melting points (T_m) of triplex template **1a**, in the presence of primer sequence (pAAGAG) and transfer tetramer (GGGA) in phosphate buffer at different pH values.[a,b]

pH value	T_m / °C
6.0	43
6.8	26
7.5	20
8.2	12

[a] Melting points are the average of the maxima of the first derivative of two heating curves and two cooling curves at rates of 1 °C/min. Melting points thus obtained agreed to with ± 1 °C.

[b] Conditions: 1 µM template **1a**, 1 µM pAAGAG, 1 µM GGGA, 1 M NaCl, 10 mM phosphate, pH adjusted to 6.0, 6.8, 7.5 or 8.2.

5. Translation Assays

Overview of chemical steps studied

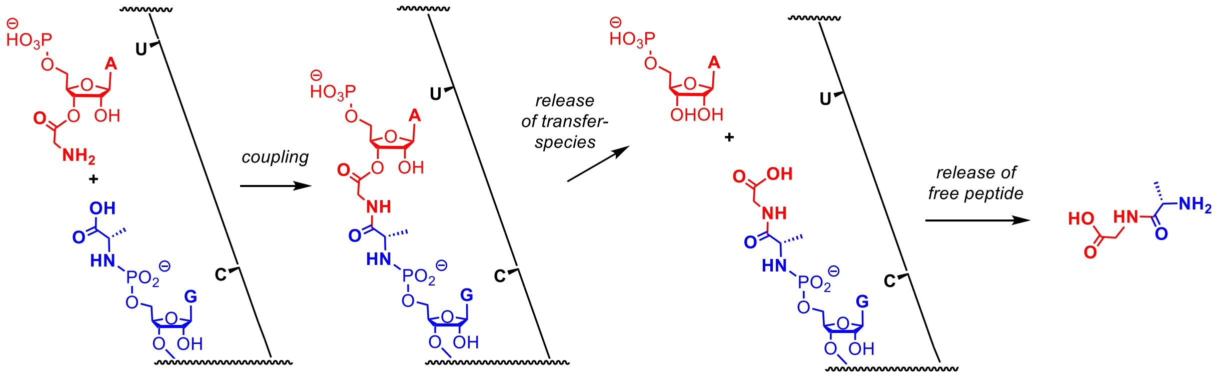

Figure S34. Representative reaction scheme showing chemical steps of ribosome-free translation, as studied in our manuscript. The first step is coupling between covalently bound amino acid residues to form the new peptide bond. This is the step that constitutes translation, as the amino acid added to the *C*-terminus of the growing peptide strand is determined by base pairing with the template. Subsequent steps are the release of the transfer species by hydrolysis and ultimately release of the free peptide by acid-catalyzed hydrolysis of the phosphoramidate linkage. This chemistry is what is shown schematically in the remainder of the manuscript.

Ribosome-free translation assays were performed in a total volume of 2.5 µL. Stock solutions of the respective template, peptido RNA, transfer species, nucleoside 5'-monophosphate (NMP), $MgCl_2$, EDC hydrochloride, and phosphate buffer (pH 6) were prepared in HPLC-grade water. The concentration of the nucleotide and oligoribonucleotide stock solutions were determined by UV-Vis spectroscopy. The final concentrations were: 20 µM peptido RNA, 20 µM template, 800 µM NMP, 0.1 M $MgCl_2$, 0.2 M EDC · HCl and 1 mM phosphate buffer pH 6, and 0.06-0.8 mM aminoacylated transfer species (800 µM transfer mononucleotide, 400 µM transfer dimer, 120 µM transfer trimer, or 60 µM transfer tetramer). Monitoring of the reaction was by MALDI-TOF MS under conditions that allow for quantitative detection.[S8]

Validation of methodology

To confirm that the detection of peptidoyl RNA products via MALDI-TOF mass spectrometry give reliable results, we isolated two representative peptidoyl RNA products of translation reactions performed on a 120 pmol scale and measured relative peak intensities for mixtures of peptido RNA educt and peptidoyl product at different ratios (Figure S35). The plots then produced 'correction factors' that can be used to correct for differences in desorption/ionization

in MALDI-TOF detection. Similar data can be found in the SI of Jash et al.[S9] The peptidoyl RNAs thus studied were GA-GlyVal$_2$Gly$_3$-AAGAG and GGA-GlyVal$_3$Gly$_3$-AAGAG. The correction factors were found to be 1.02 and 1.1, respectively. This is a too small deviation from identical desorption/ionization properties that no correction was deemed necessary when reading out kinetic data from MALDI spectra.

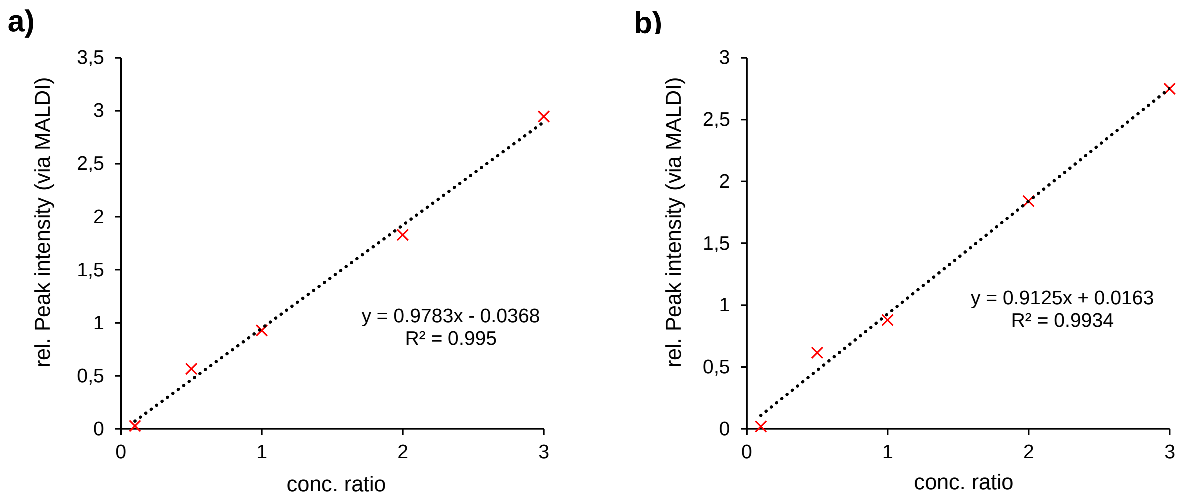

Figure S35. Quantitative MALDI-TOF MS under assay conditions[S8]: Representative calibration plots for the determination of correction factors for the detection of translation products, showing differences in desorption/ionization efficiency in MALDI-TOF MS, or lack thereof, for starting material and product of a translation reaction. The ratio of relative intensities, as determined by MALDI MS, is plotted against the ratio of concentrations, as determined by UV absorbance. a) GA-GlyVal$_2$Gly$_3$-AAGAG, and b) GGA-GlyVal$_3$Gly$_3$-AAGAG. The dotted lines are the fits from linear regression.

The second validation was performed by chromatography. Here, it was demonstrated that near-quantitative conversion, as measured by MALDI-TOF MS, corresponds to no detectable educt in a chromatographic analysis. Because our assays are performed on a too small scale for HPLC analysis with conventional equipment, we performed chromatography on a C18 cartridge (RP SEP-PAK VAC C18 1CC, 100 mg, *Waters*, Milford, MA, USA). The crude was applied in 5 M NaCl solution, followed by elution with a gradient of 1 M NaCl, pure water, 6% ACN, 10% ACN, and a wash with 20 % ACN. Fractions of 3 drops were collected and analyzed by UV absorption (Nanodrop spectrometer) and MALDI-TOF MS, resulting in the chromatogram shown in Figure S36, below. It can be discerned that no remaining peptido RNA educt was detectable under those conditions.

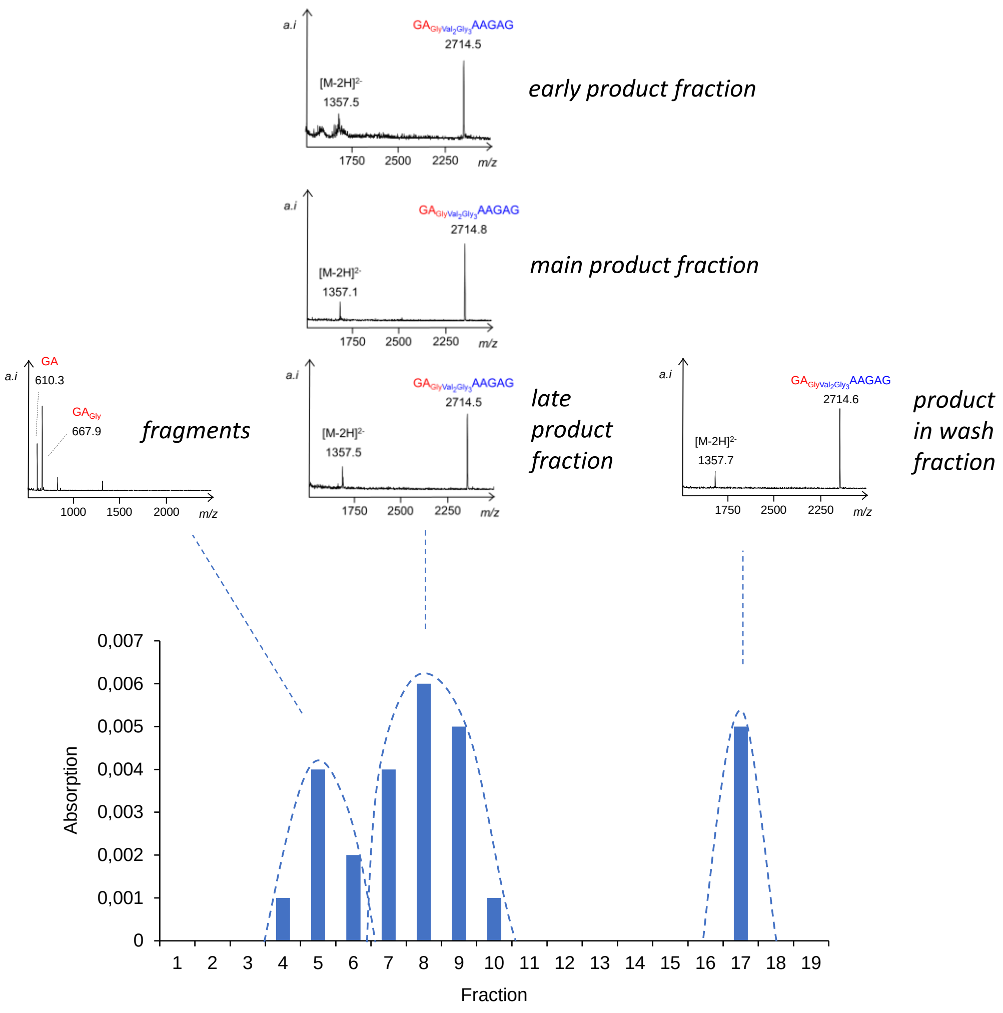

Figure S36. Chromatographic analysis of translation mixture producing hexapeptidoyl RNA GA-GlyVal$_2$Gly$_3$-AAGAG, in an assay under standard conditions (20 µM Val$_2$Gly$_3$-AAGAG, 20 µM template **1a**, 400 µM GA-Gly, 800 µM CMP, 0.1 M $MgCl_2$, 0.2 M EDC, 1 mM phosphate buffer pH 6, 0 °C), as obtained after 24 h reaction time. Fractions eluting from the C18 micro-column were analyzed by UV absorption at 260 nm, producing the bars shown in blue. They were then subjected to MALDI-TOF analysis, with representative spectra shown above the bars. The dashed lines indicate the elution profile underlying the pattern of bars and the correlation between peaks and mass spectra. No other products were detected, confirming that mass spectrometric detection of full conversion corresponds to chromatographic detection of full conversion.

For fits to kinetic data, the following monoexponential equation was used.

$$y = y_{\max}\,(1 - e^{-kt})$$

Here, y corresponds to the yield (conversion to peptidoyl RNA), y_{max} the calculated maximum yield of the reaction at infinite reaction time, k the rate constant, and t the time in hours. Half-conversion times were calculated from rate constants with $t_{1/2} = \ln(2) / k$.

Pentapeptidoyl RNAs

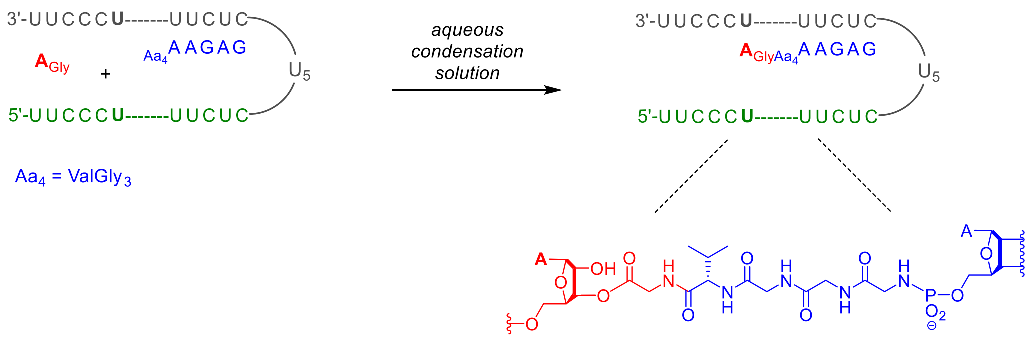

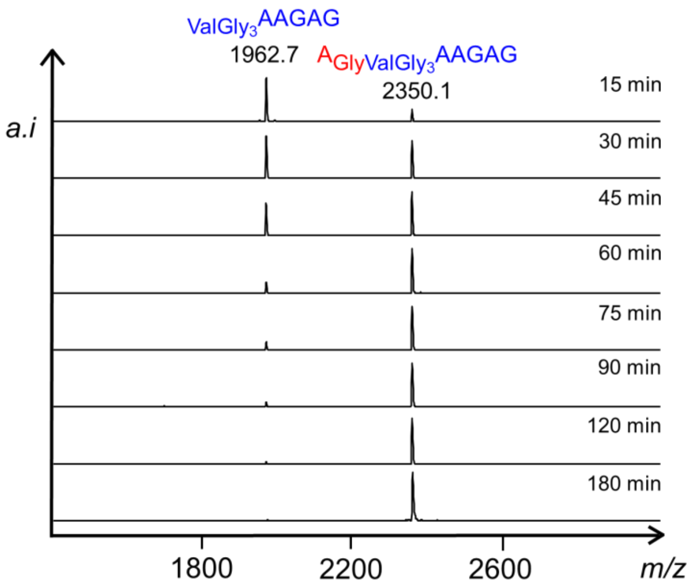

Figure S37. Reaction scheme and MALDI-TOF mass spectra from translation assay with 20 µM ValGly$_3$-AAGAG, 20 µM template **1a**, 800 µM A-Gly, 800 µM CMP, 0.1 M $MgCl_2$, 0.2 M EDC · HCl, 1 mM phosphate buffer pH 6, 0 °C at the time points given.

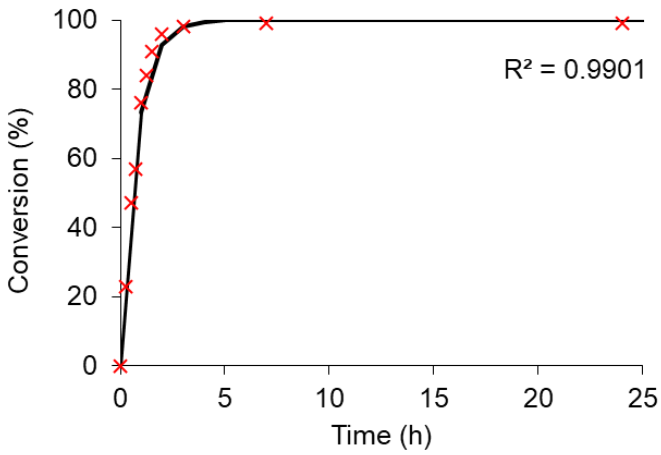

Figure S38. Kinetics of translation with ValGly$_3$-AAGAG, A-Gly and template **1a**; shown are experimental data points and monoexponential fit.

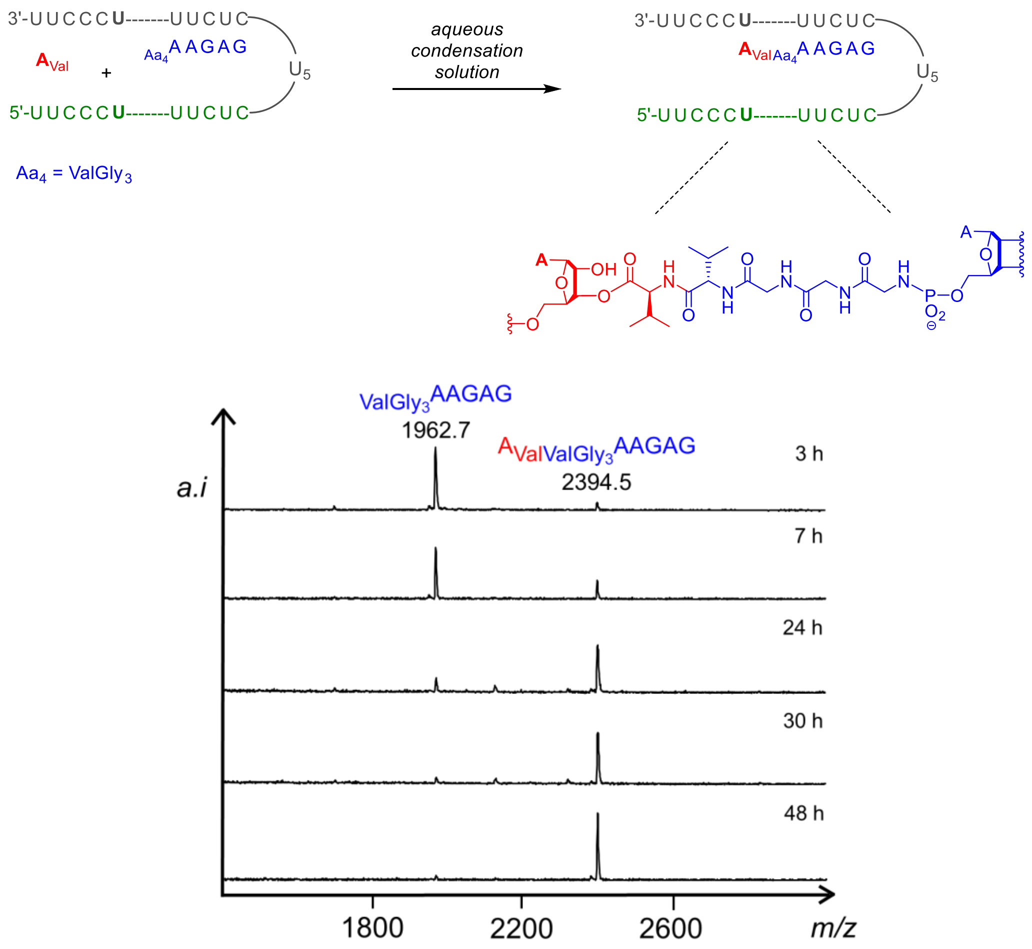

Figure S39. Reaction scheme and MALDI-TOF mass spectra from translation assay with 20 µM ValGly3-AAGAG, 20 µM template **1a**, 800 µM A-Val, 800 µM CMP, 0.1 M $MgCl_2$, 0.2 M EDC · HCl, 1 mM phosphate buffer pH 6, 0 °C at the time points given.

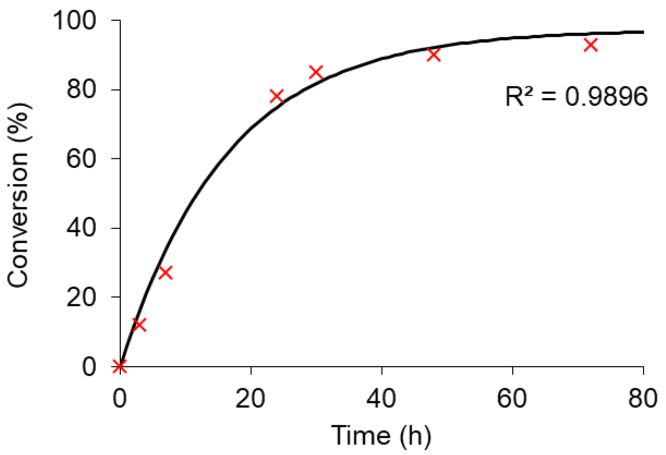

Figure S40. Kinetics of translation with ValGly3-AAGAG, A-Val and template **1a**; shown are experimental data points and monoexponential fit.

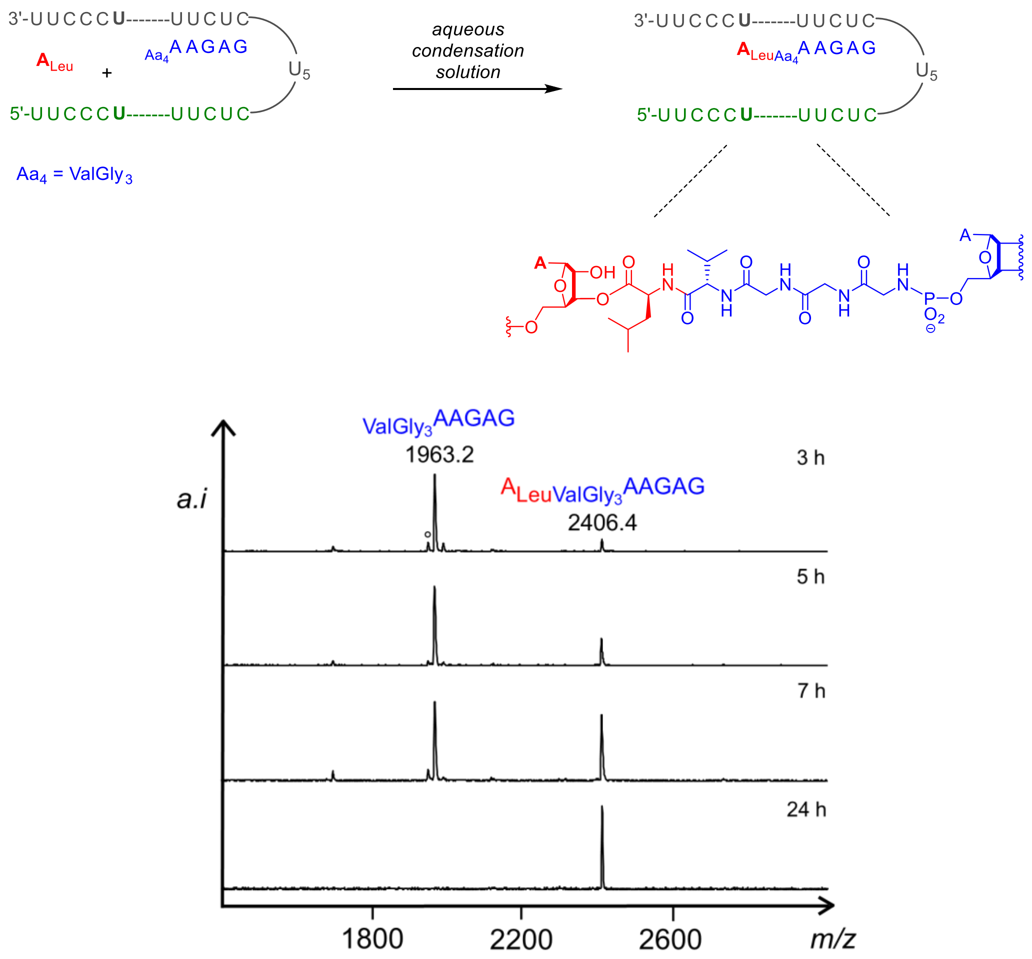

Figure S41. Reaction scheme and MALDI-TOF mass spectra from translation assay with 20 µM ValGly$_3$-AAGAG, 20 µM template **1a**, 800 µM A-Leu, 800 µM CMP, 0.1 M $MgCl_2$, 0.2 M EDC · HCl, 1 mM phosphate buffer pH 6, 0 °C at the time points given. The peak labeled with a circle is for a mass 18 Da lower than that of the peptido RNA educt and most probably caused by dehydration.

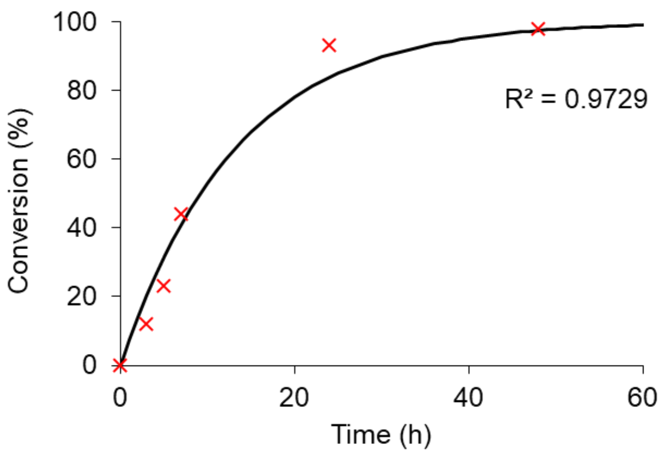

Figure S42. Kinetics of translation with ValGly$_3$-AAGAG, A-Leu and template **1a**; shown are experimental data points and monoexponential fit.

Hexapeptidoyl RNAs

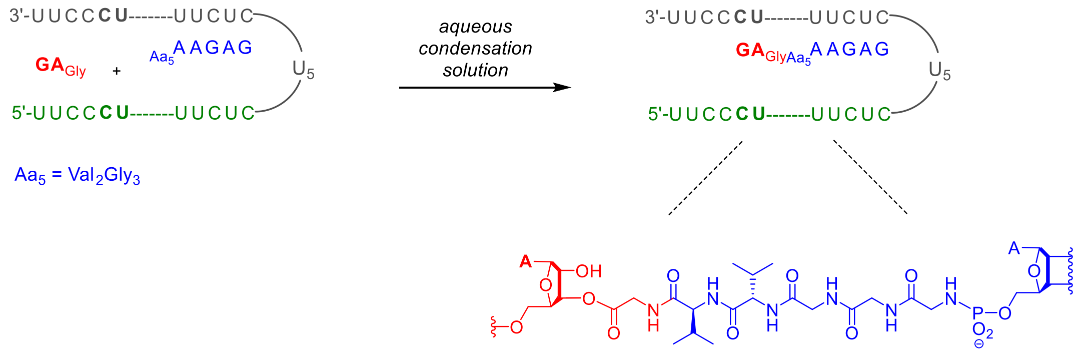

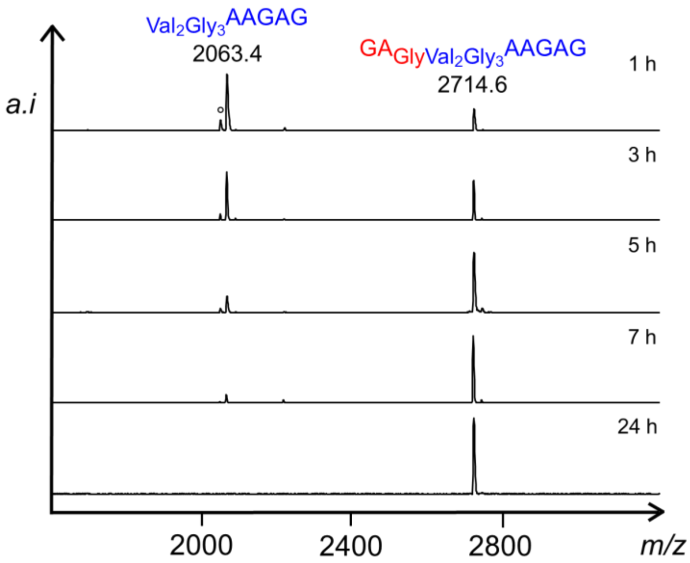

Figure S43. Reaction scheme and MALDI-TOF mass spectra from translation assay with 20 µM Val_2Gly_3-AAGAG, 20 µM template **1a**, 400 µM GA-Gly, 800 µM CMP, 0.1 M $MgCl_2$, 0.2 M EDC · HCl, 1 mM phosphate buffer pH 6, 0 °C at the time points given. The peak labeled with a circle is for a mass 18 Da lower than that of the peptido RNA educt and most probably caused by dehydration.

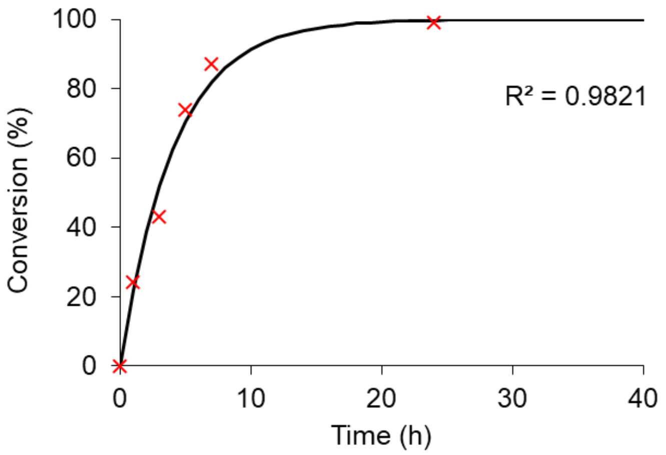

Figure S44. Kinetics of translation with Val_2Gly_3-AAGAG, GA-Gly and template **1a**; shown are experimental data points and monoexponential fit.

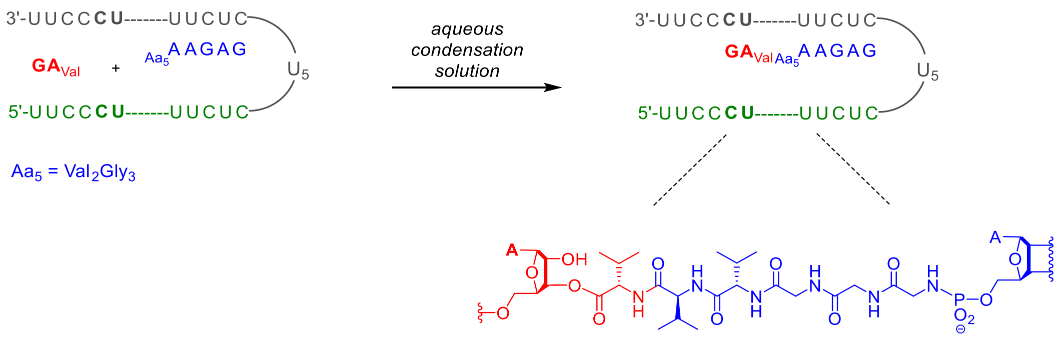

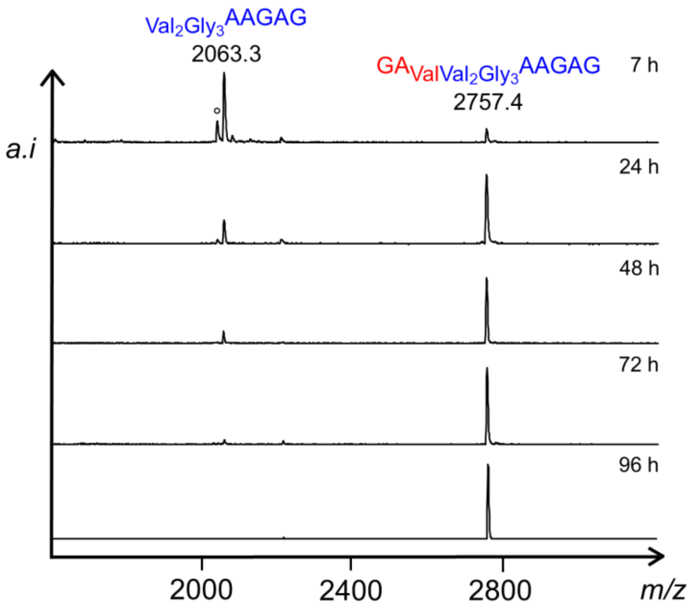

Figure S45. Reaction scheme and MALDI-TOF mass spectra from translation assay with 20 µM Val_2Gly_3-AAGAG, 20 µM template **1a**, 400 µM GA-Val, 800 µM CMP, 0.1 M $MgCl_2$, 0.2 M EDC · HCl, 1 mM phosphate buffer pH 6, 0 °C at the time points given. The peak labeled with a circle is for a mass 18 Da lower than that of the peptido RNA educt and most probably caused by dehydration.

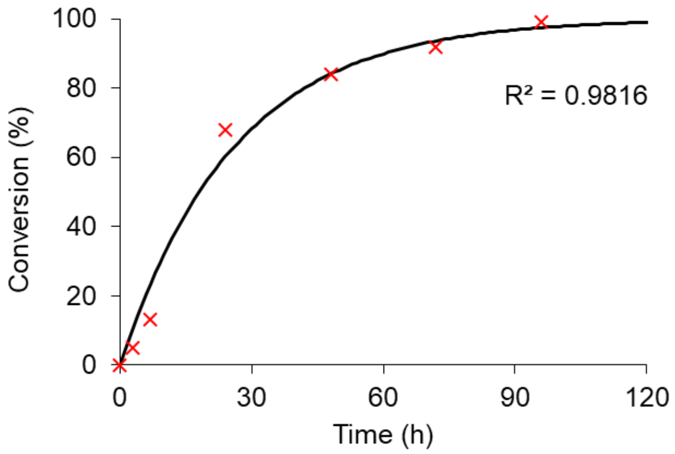

Figure S46. Kinetics of translation with Val_2Gly_3-AAGAG, GA-Val and template **1a**; shown are experimental data points and monoexponential fit.

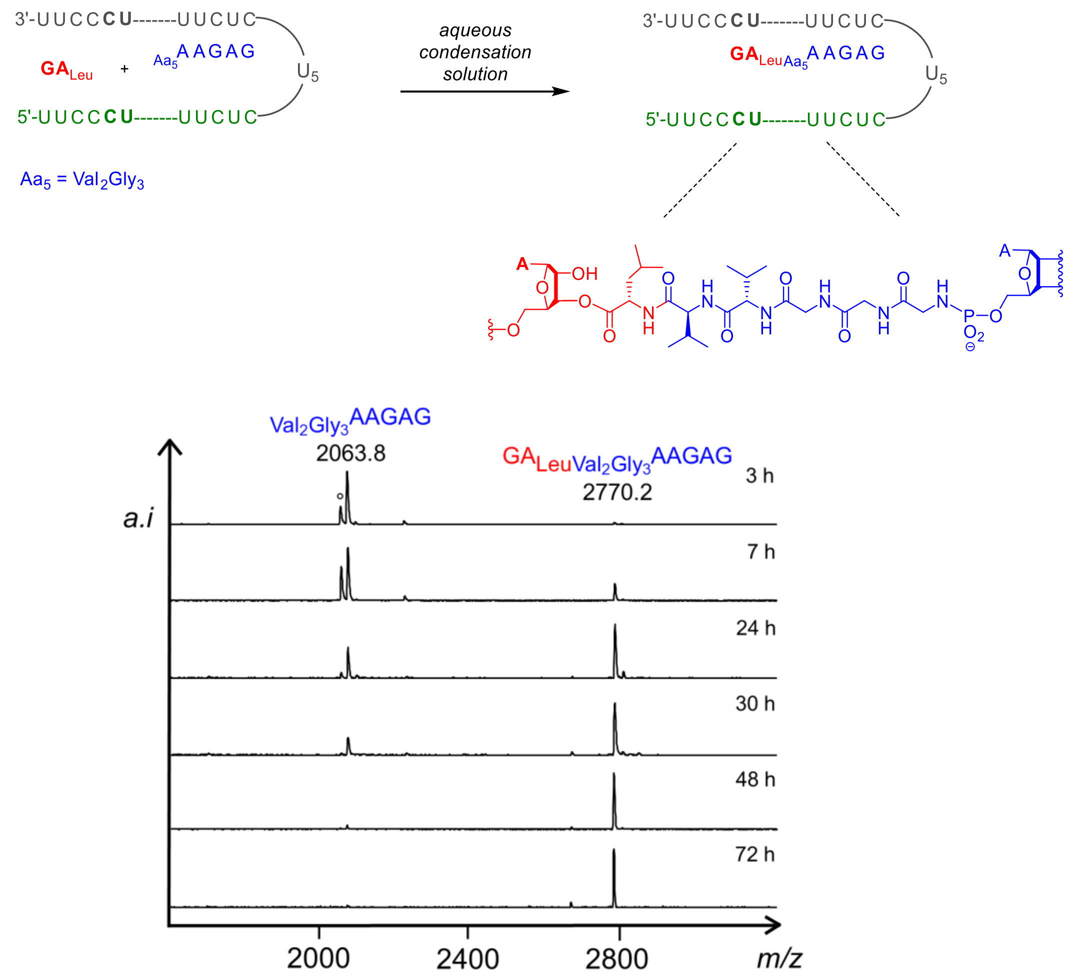

Figure S47. Reaction scheme and MALDI-TOF mass spectra from translation assay with 20 µM Val_2Gly_3-AAGAG, 20 µM template **1a**, 400 µM GA-Leu, 800 µM CMP, 0.1 M $MgCl_2$, 0.2 M EDC · HCl, 1 mM phosphate buffer pH 6, 0 °C at the time points given. The peak labeled with a circle is for a mass 18 Da lower than that of the peptido RNA educt and most probably caused by dehydration.

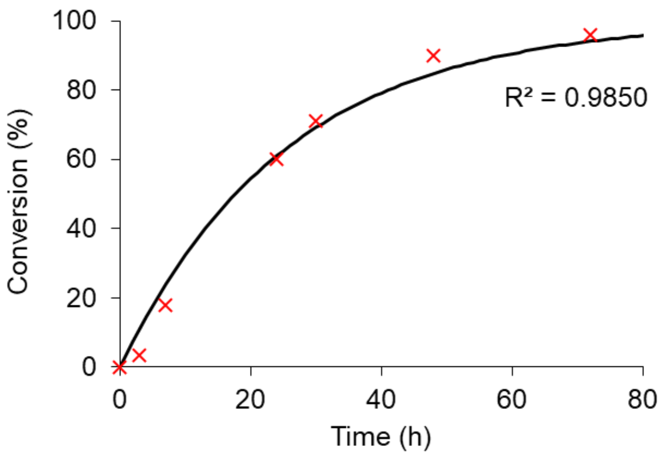

Figure S48. Kinetics of translation with Val_2Gly_3-AAGAG, GA-Leu and template **1a**; shown are experimental data points and monoexponential fit.

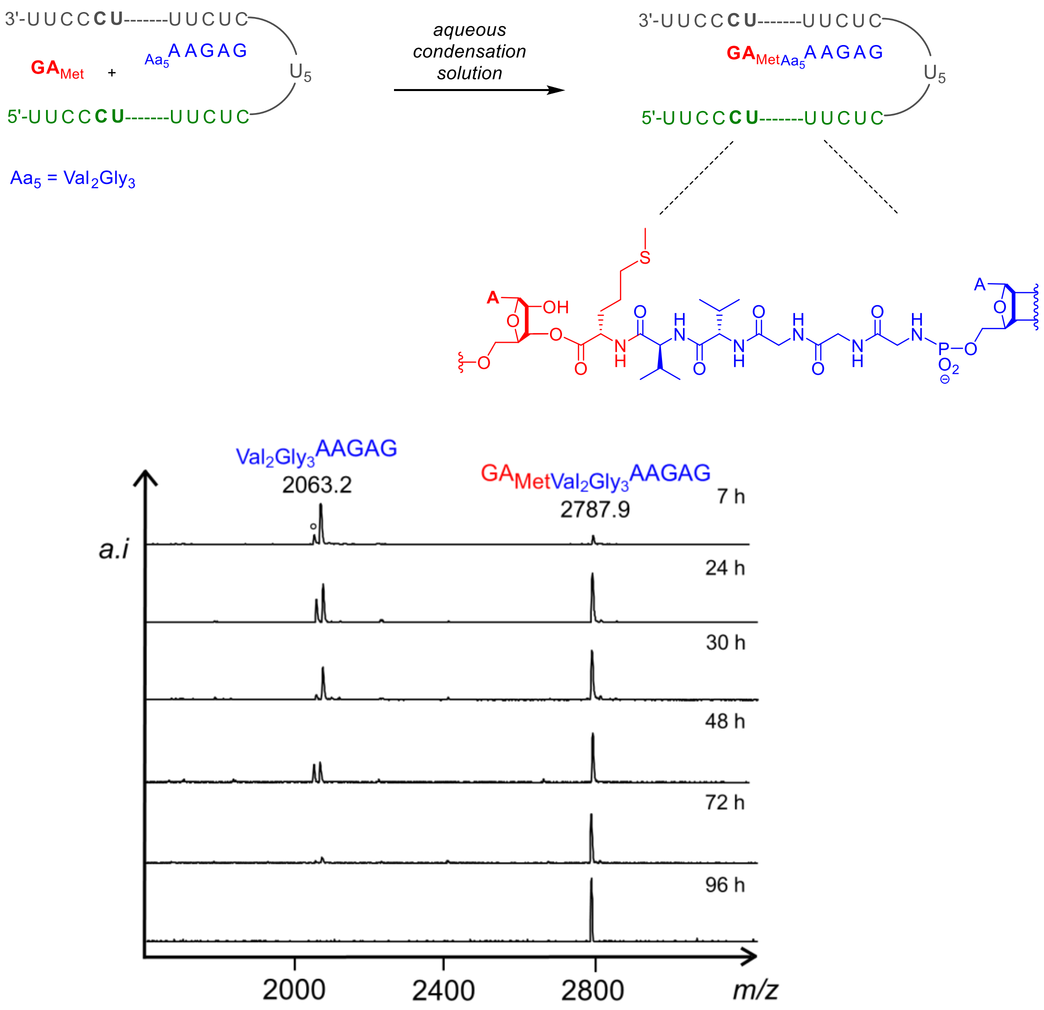

Figure S49. Reaction scheme and MALDI-TOF mass spectra from translation assay with 20 µM Val_2Gly_3-AAGAG, 20 µM template **1a**, 400 µM GA-Met, 800 µM CMP, 0.1 M $MgCl_2$, 0.2 M EDC · HCl, 1 mM phosphate buffer pH 6, 0 °C at the time points given. The peak labeled with a circle is for a mass 18 Da lower than that of the peptido RNA educt and most probably caused by dehydration.

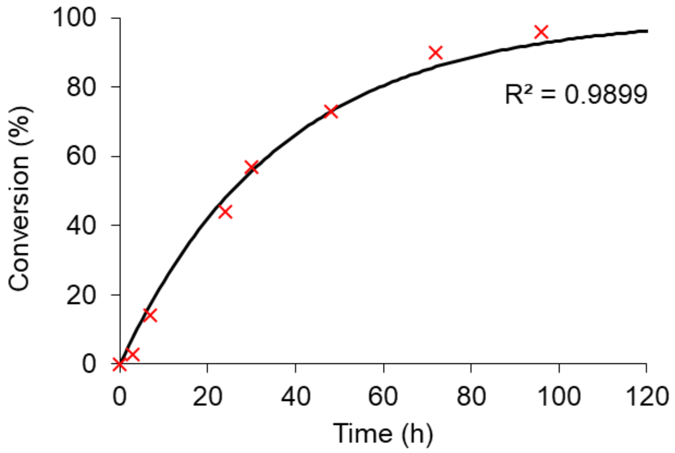

Figure S50. Kinetics of translation with Val_2Gly_3-AAGAG, GA-Met and template **1a**; shown are experimental data points and monoexponential fit.

Heptapeptidoyl RNAs

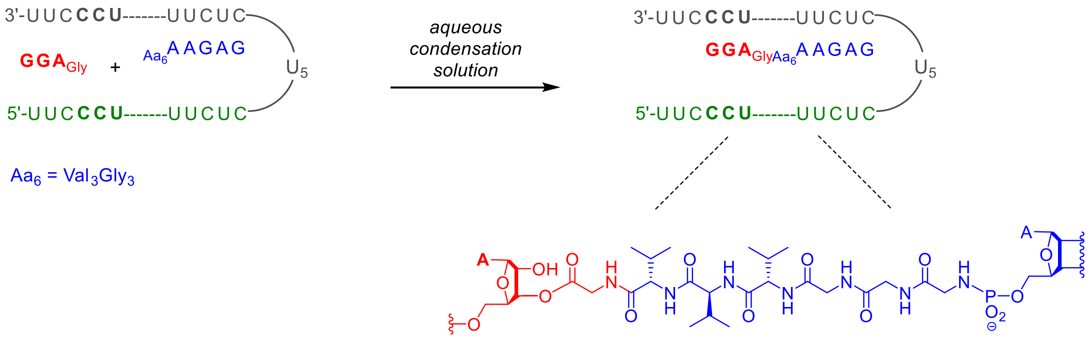

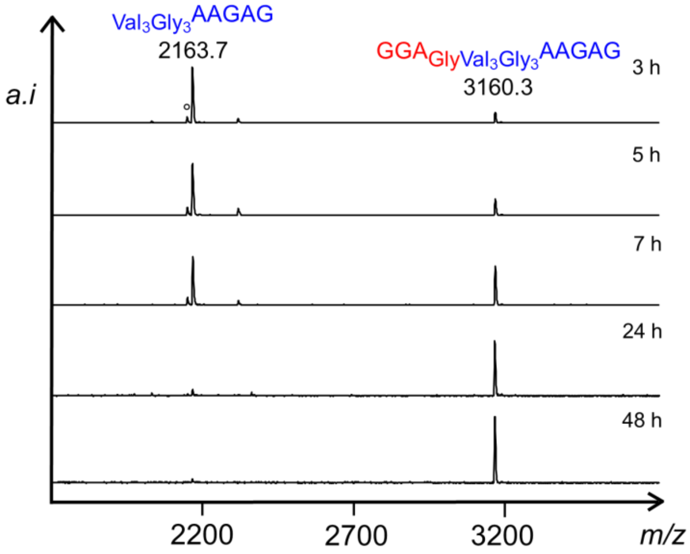

Figure S51. Reaction scheme and MALDI-TOF mass spectra from translation assay with 20 µM Val_3Gly_3-AAGAG, 20 µM template **1a**, 120 µM GGA-Gly, 800 µM CMP, 0.1 M $MgCl_2$, 0.2 M EDC · HCl, 1 mM phosphate buffer pH 6, 0 °C at different time points. The peak labeled with a circle is for a mass 18 Da lower than that of the peptido RNA educt and most probably caused by dehydration.

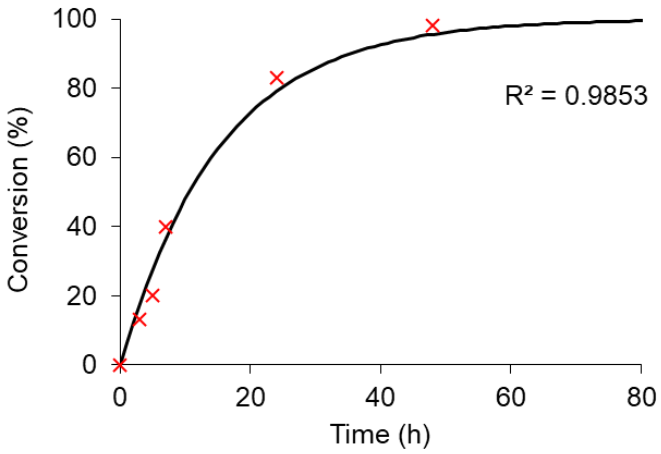

Figure S52. Kinetics of translation with Val_3Gly_3-AAGAG, GGA-Gly and template **1a**; shown are experimental data points and monoexponential fit.

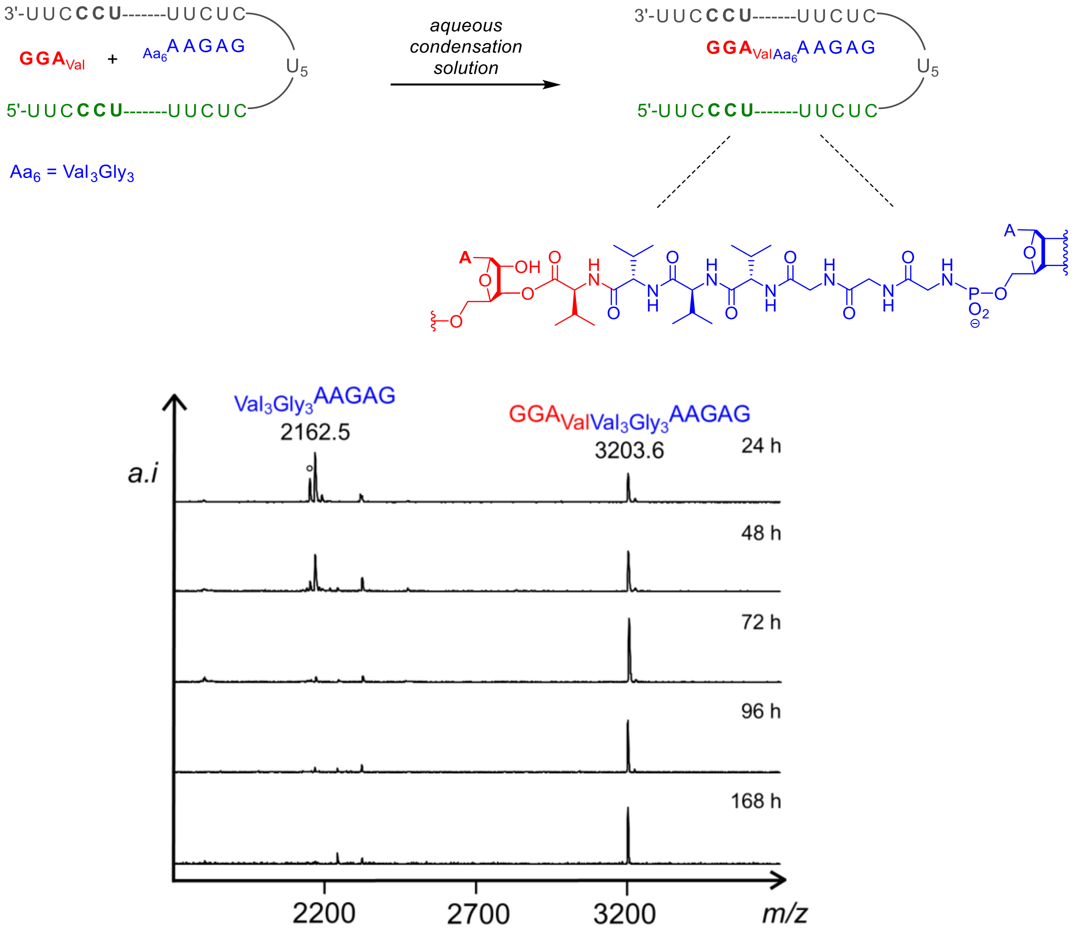

Figure S53. Reaction scheme and MALDI-TOF mass spectra from translation assay with 20 µM Val_3Gly_3-AAGAG, 20 µM template **1a**, 120 µM GGA-Val, 800 µM CMP, 0.1 M $MgCl_2$, 0.2 M EDC · HCl, 1 mM phosphate buffer pH 6, 0 °C at the time points given. The peak labeled with a circle is for a mass 18 Da lower than that of the peptido RNA educt and most probably caused by dehydration.

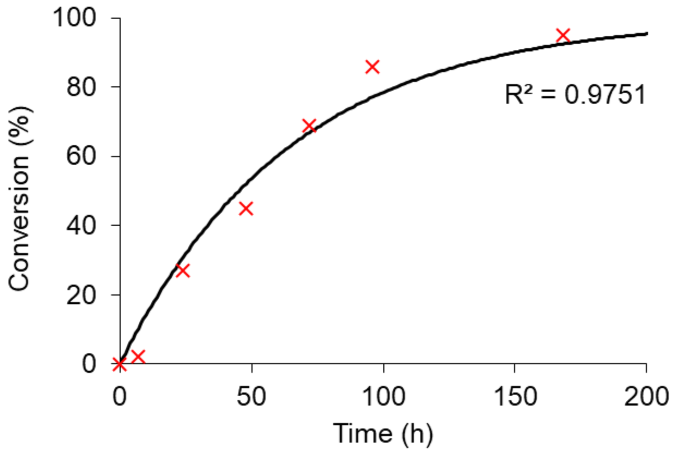

Figure S54. Kinetics of translation with Val_3Gly_3-AAGAG, GGA-Val and template **1a**; shown are experimental data points and monoexponential fit.

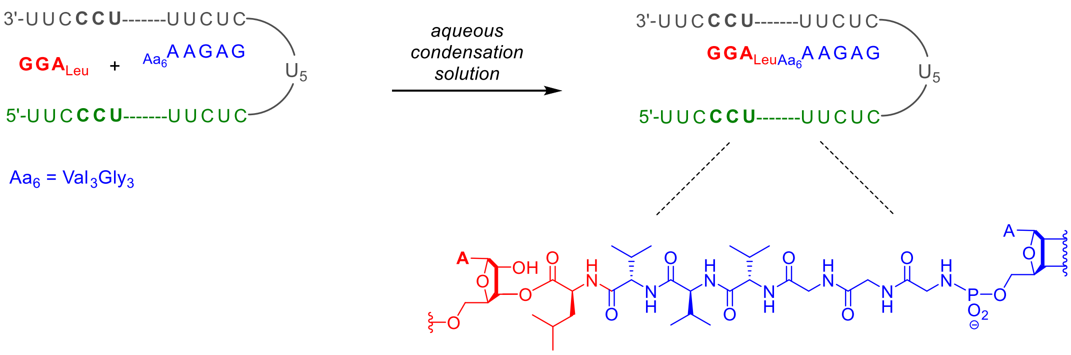

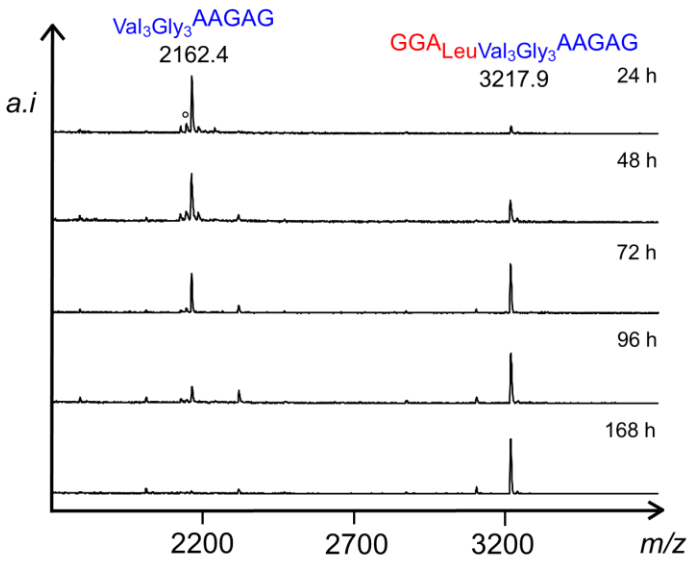

Figure S55. Reaction scheme and MALDI-TOF mass spectra from translation assay with 20 µM Val_3Gly_3-AAGAG, 20 µM template **1a**, 120 µM GGA-Leu, 800 µM CMP, 0.1 M $MgCl_2$, 0.2 M EDC · HCl, 1 mM phosphate buffer pH 6, 0 °C at the time points given. The peak labeled with a circle is for a mass 18 Da lower than that of the peptido RNA educt and most probably caused by dehydration.

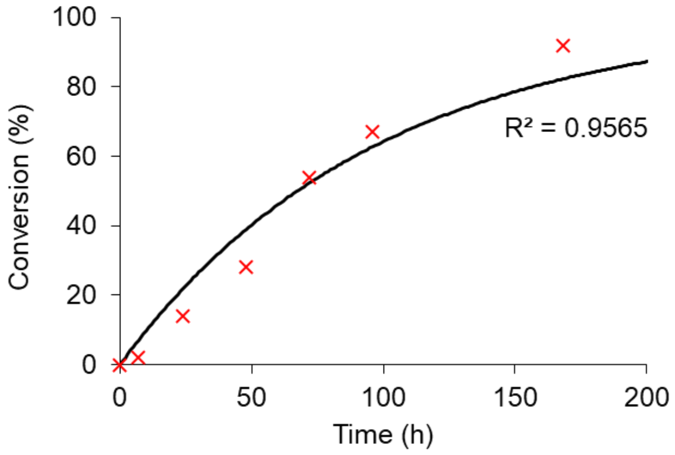

Figure S56. Kinetics of translation with Val_3Gly_3-AAGAG, GGA-Leu and template **1a**; shown are experimental data points and monoexponential fit.

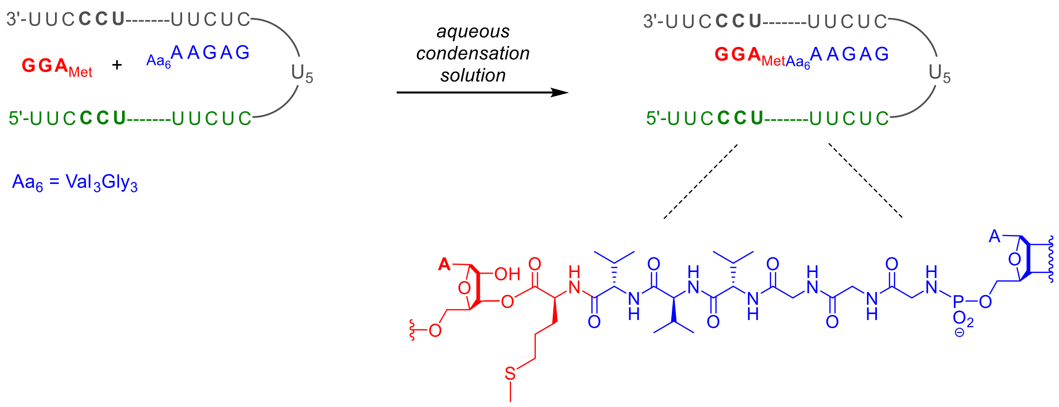

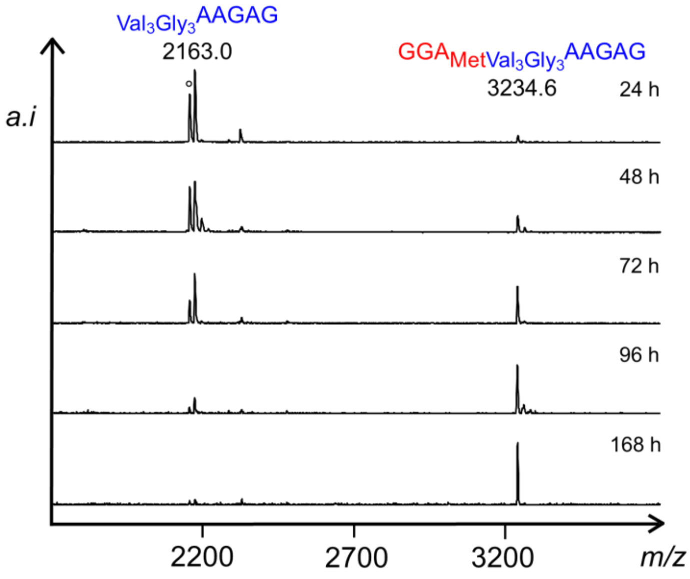

Figure S57. Reaction scheme and MALDI-TOF mass spectra from translation assay with 20 µM Val_3Gly_3-AAGAG, 20 µM template **1a**, 120 µM GGA-Met, 800 µM CMP, 0.1 M $MgCl_2$, 0.2 M EDC · HCl, 1 mM phosphate buffer pH 6, 0 °C at the time points given. The peak labeled with a circle is for a mass 18 Da lower than that of the peptido RNA educt and most probably caused by dehydration.

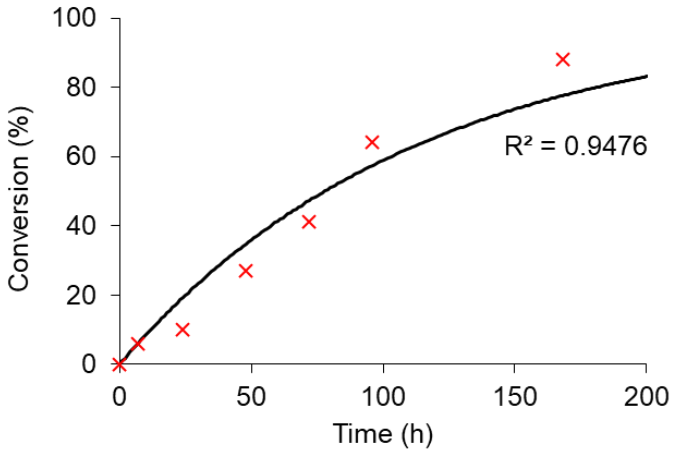

Figure S58. Kinetics of translation with Val_3Gly_3-AAGAG, GGA-Met and template **1a**; shown are experimental data points and monoexponential fit.

Octapeptidoyl RNAs

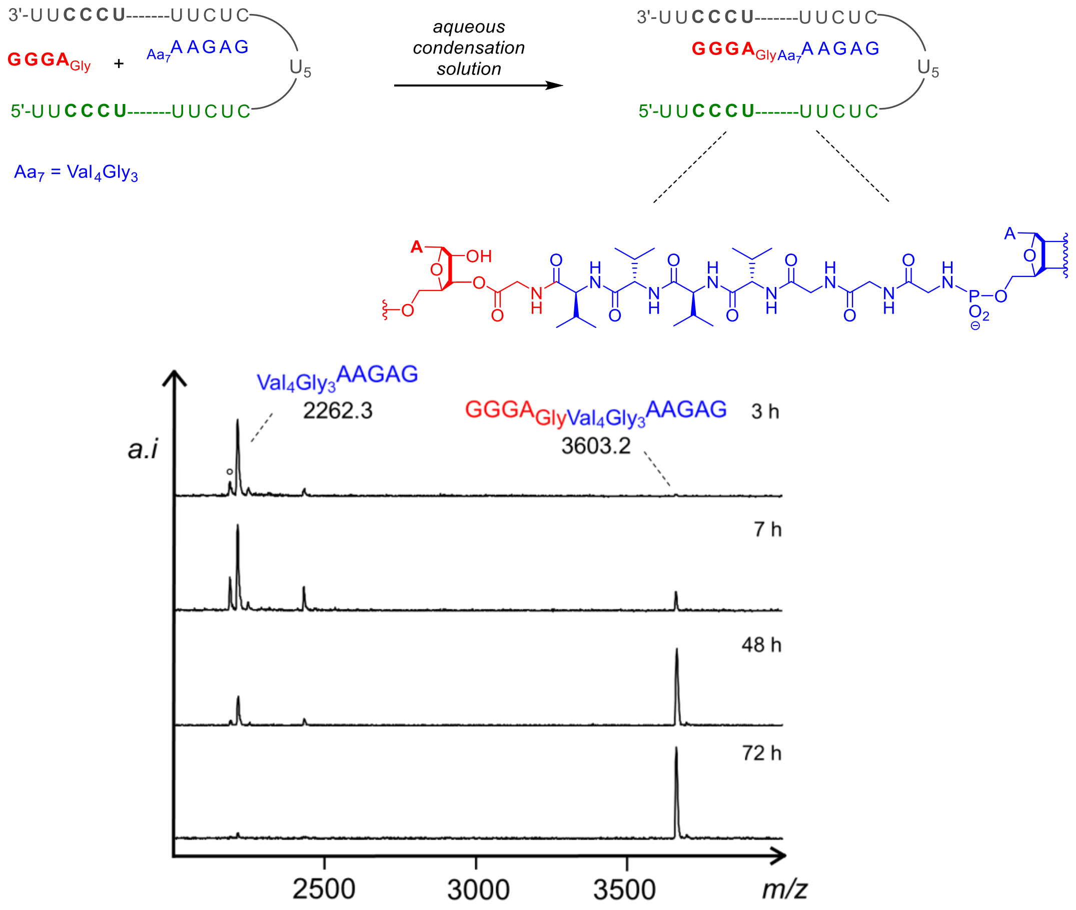

Figure S59. Reaction scheme and MALDI-TOF mass spectra from translation assay with 20 µM Val_4Gly_3-AAGAG, 20 µM template **1a**, 60 µM GGGA-Gly, 800 µM CMP, 0.1 M $MgCl_2$, 0.2 M EDC · HCl, 1 mM phosphate buffer pH 6, 0 °C at the time points given. The peak labeled with a circle is for a mass 18 Da lower than that of the peptido RNA educt and most probably caused by dehydration.

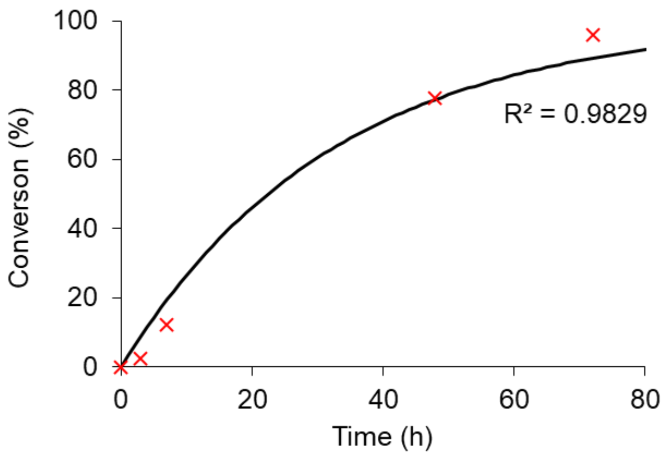

Figure S60. Kinetics of translation with Val_4Gly_3-AAGAG, GGGA-Gly and template **1a**; shown are experimental data points and monoexponential fit.

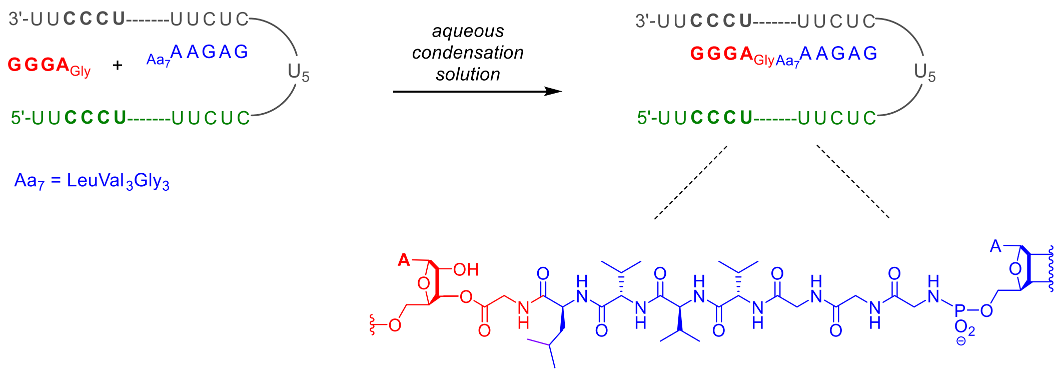

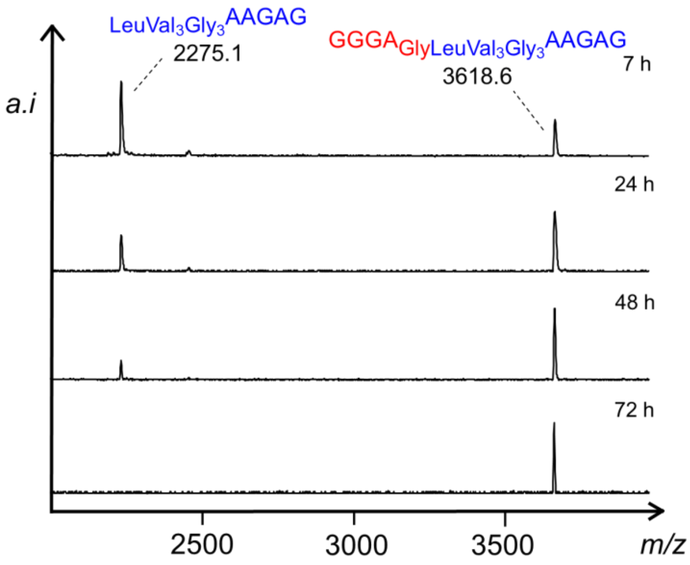

Figure S61. Reaction scheme and MALDI-TOF mass spectra from translation assay with 20 µM LeuVal$_3$Gly$_3$-AAGAG, 20 µM template **1a**, 60 µM GGGA-Gly, 800 µM CMP, 0.1 M $MgCl_2$, 0.2 M EDC · HCl, 1 mM phosphate buffer pH 6, 0 °C at the time points given.

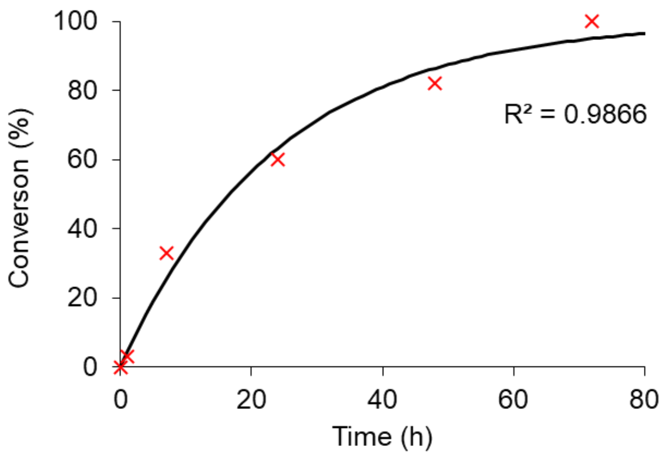

Figure S62. Kinetics of translation with LeuVal$_3$Gly$_3$-AAGAG, GGGA-Gly and template **1a**; shown are experimental data points and monoexponential fit.

Table 10. Kinetic data for translations producing peptidoyl RNAs up to the octapeptide level.[a]

Entry No.	Transfer species	Peptido RNA	Rate constant k $\times 10^2$ (h^{-1}) [b]	$t_{1/2}$ of reaction (h) [c]
1	A-Gly	$ValGly_3$-AAGAG	132	0.5
2	A-Val	$ValGly_3$-AAGAG	6.1	11
3	A-Leu	$ValGly_3$-AAGAG	7.6	9.1
4	GA-Gly	Val_2Gly_3-AAGAG	25	2.7
5	GA-Val	Val_2Gly_3-AAGAG	3.8	18
6	GA-Leu	Val_2Gly_3-AAGAG	4.1	17
7	GA-Met	Val_2Gly_3-AAGAG	2.8	25
8	GGA-Gly	Val_3Gly_3-AAGAG	6.5	11
9	GGA-Val	Val_3Gly_3-AAGAG	1.5	45
10	GGA-Leu	Val_3Gly_3-AAGAG	1.0	67
11	GGA-Met	Val_3Gly_3-AAGAG	0.9	78
12	GGGA-Gly	$LeuVal_3Gly_3$-AAGAG	4.2	17
13	GGGA-Gly	Val_4Gly_3-AAGAG	3.1	22

[a] See legend to Figures S37-S61 for conditions. Full or near-quantitative conversion was observed in each case.

[b] Monoexponential fit to experimental data, assuming a pseudo-first order reaction of the template-bound reaction partners.

[c] Time to reach 50% conversion to peptidoyl RNA product.

6. Assays with more than one transfer species

Pentapeptidoyl RNA

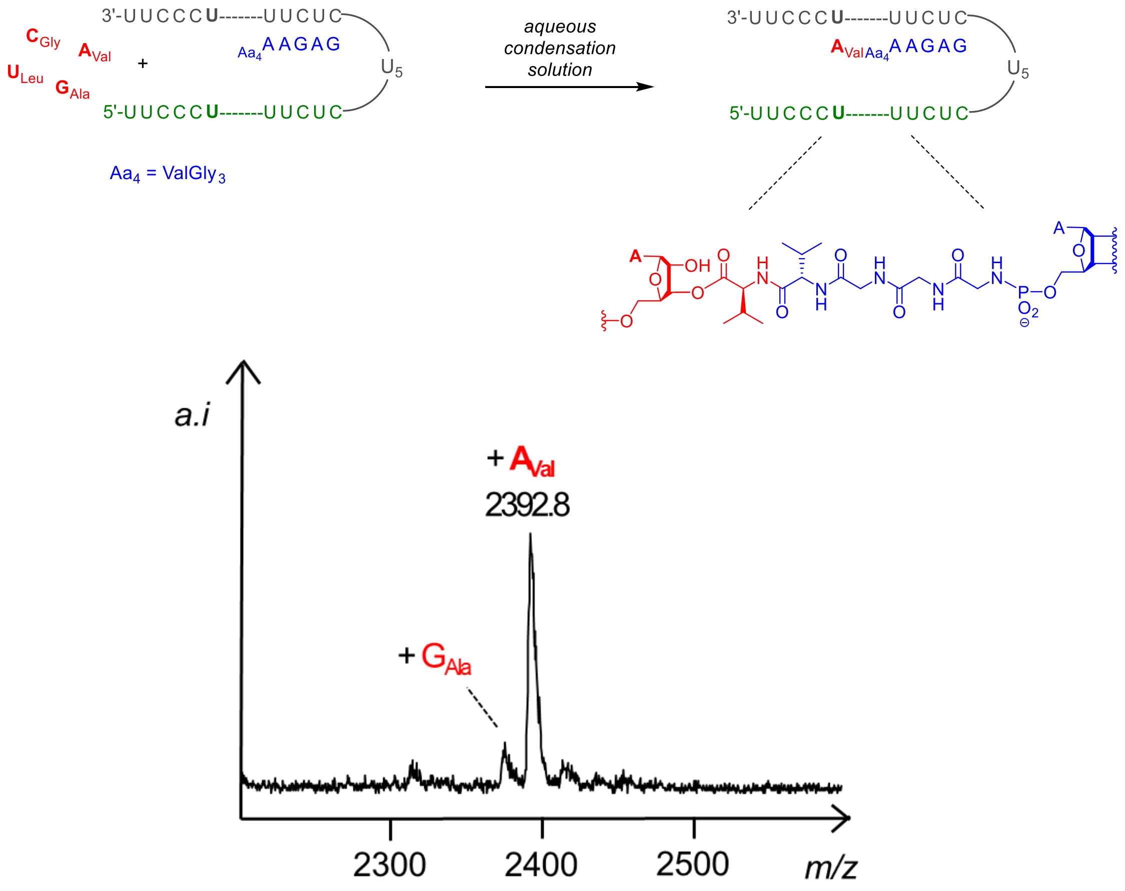

Figure S63. Reaction scheme and MALDI-TOF mass spectrum from translation assay with 20 µM ValGly3-AAGAG, 20 µM template **1a**, 800 µM A-Val, 800 µM G-Ala, 800 µM C-Gly, 800 µM U-Leu, 800 µM CMP, 0.1 M $MgCl_2$, 0.2 M EDC · HCl, 1 mM phosphate buffer pH 6, 0 °C.

Table S11. Fidelity of translation with four aminoacylated transfer monomers, peptido RNA ValGly3-AAGAG and template **1a** as detected after 48 h.[a]

Product	Conversion (%)	$[M-H]^-$ m/z calc.	$[M-H]^-$ m/z found
A-ValValGly3-AAGAG	77	2390.7	2392.8
G-AlaValGly3-AAGAG	5	2376.6	2377.2
C-GlyValGly3-AAGAG	-	2324.7	n.d.
U-LeuValGly3-AAGAG	-	2381.7	n.d.

[a] Conditions for the assay are provided in the legend to Figure S63.

Hexapeptidoyl RNA

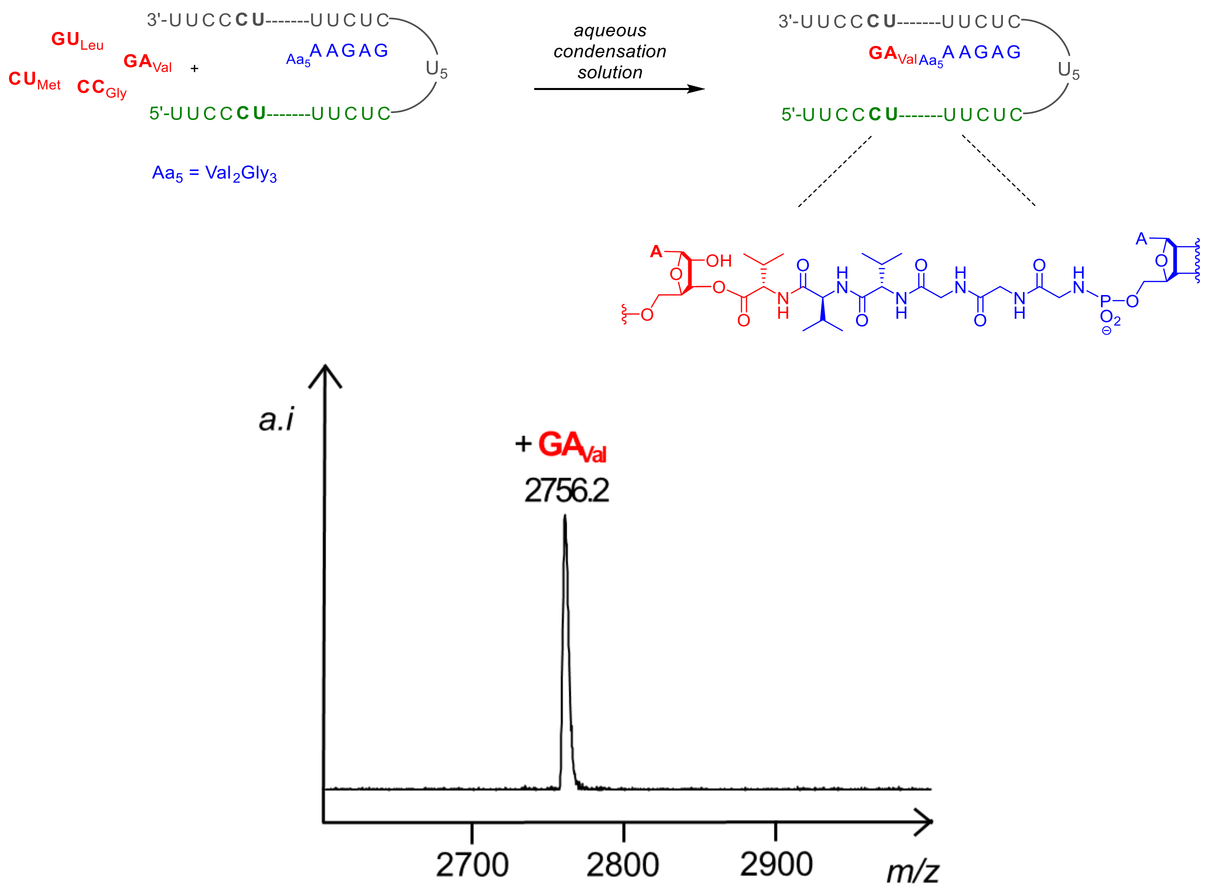

Figure S64. Reaction scheme and MALDI-TOF mass spectrum from translation assay with 20 µM Val_2Gly_3-AAGAG, 20 µM template **1a**, 400 µM GA-Val, 400 µM CC-Gly, 400 µM CU-Met, 400 µM GU-Leu, 800 µM CMP, 0.1 M $MgCl_2$, 0.2 M EDC · HCl, 1 mM phosphate buffer pH 6, 0 °C.

Table S12. Fidelity of translation with four aminoacylated transfer dimers, peptido RNA Val_2Gly_3-AAGAG and template **1a** as detected after 48 h.[a]

Product	Conversion (%)	$[M-H]^-$ m/z calc.	$[M-H]^-$ m/z found
GA-ValVal_2Gly_3-AAGAG	87	2753.0	2756.2
CC-GlyVal_2Gly_3-AAGAG	<1	2646.9	2648.8
CU-MetVal_2Gly_3-AAGAG	-	2722.0	n.d.
GU-LeuVal_2Gly_3-AAGAG	-	2744.0	n.d.

[a] Conditions for the assay are provided in the legend to Figure S64.

Heptapeptidoyl RNA

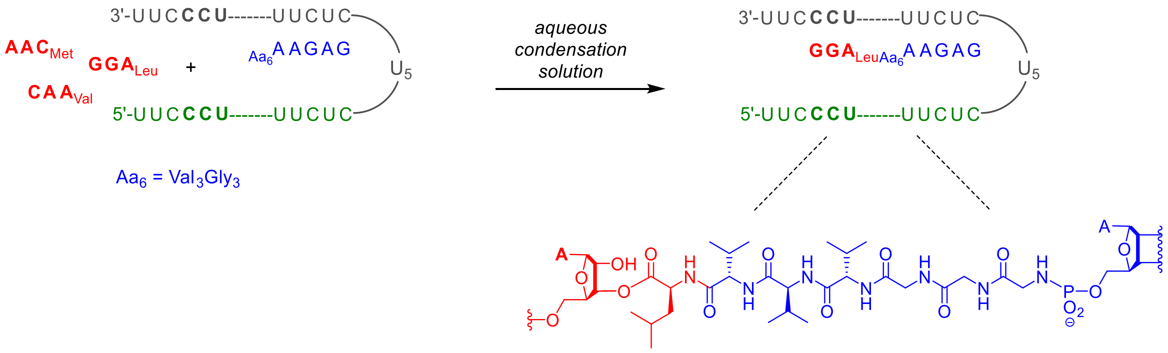

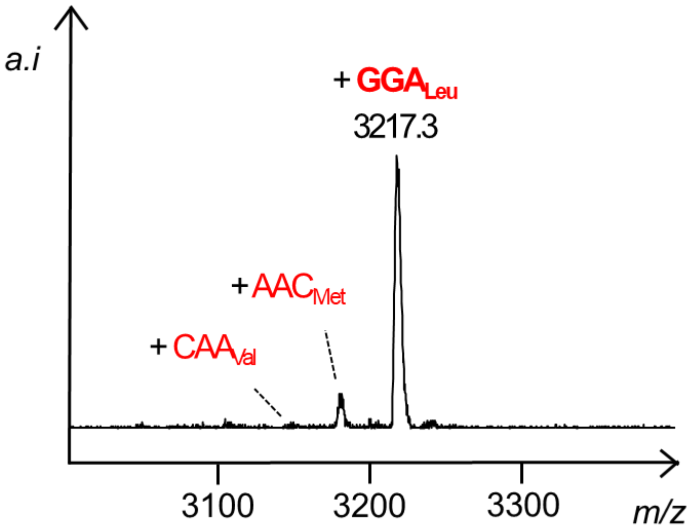

Figure S65. Reaction scheme and MALDI-TOF mass spectrum from translation assay with 20 µM Val_3Gly_3-AAGAG, 20 µM template **1a**, 120 µM GGA-Leu, 120 µM CAA-Val, 120 µM AAC-Met, 800 µM CMP, 0.1 M $MgCl_2$, 0.2 M EDC · HCl, 1 mM phosphate buffer pH 6, 0 °C.

Table S13. Fidelity of translation with three aminoacylated transfer trimers, peptido RNA Val_3Gly_3-AAGAG and template **1a** as detected after 120 h.[a]

Product	Conversion (%)	$[M-H]^-$ m/z calc.	$[M-H]^-$ m/z found
GGA-LeuVal_3Gly_3-AAGAG	48	3215.4	3217.3
AAC-MetVal_3Gly_3-AAGAG	6	3173.4	3175.0
CAA-ValVal_3Gly_3-AAGAG	1	3145.3	3148.2

[a] Conditions for the assay are provided in the legend to Figure S65.

Octapeptidoyl RNA

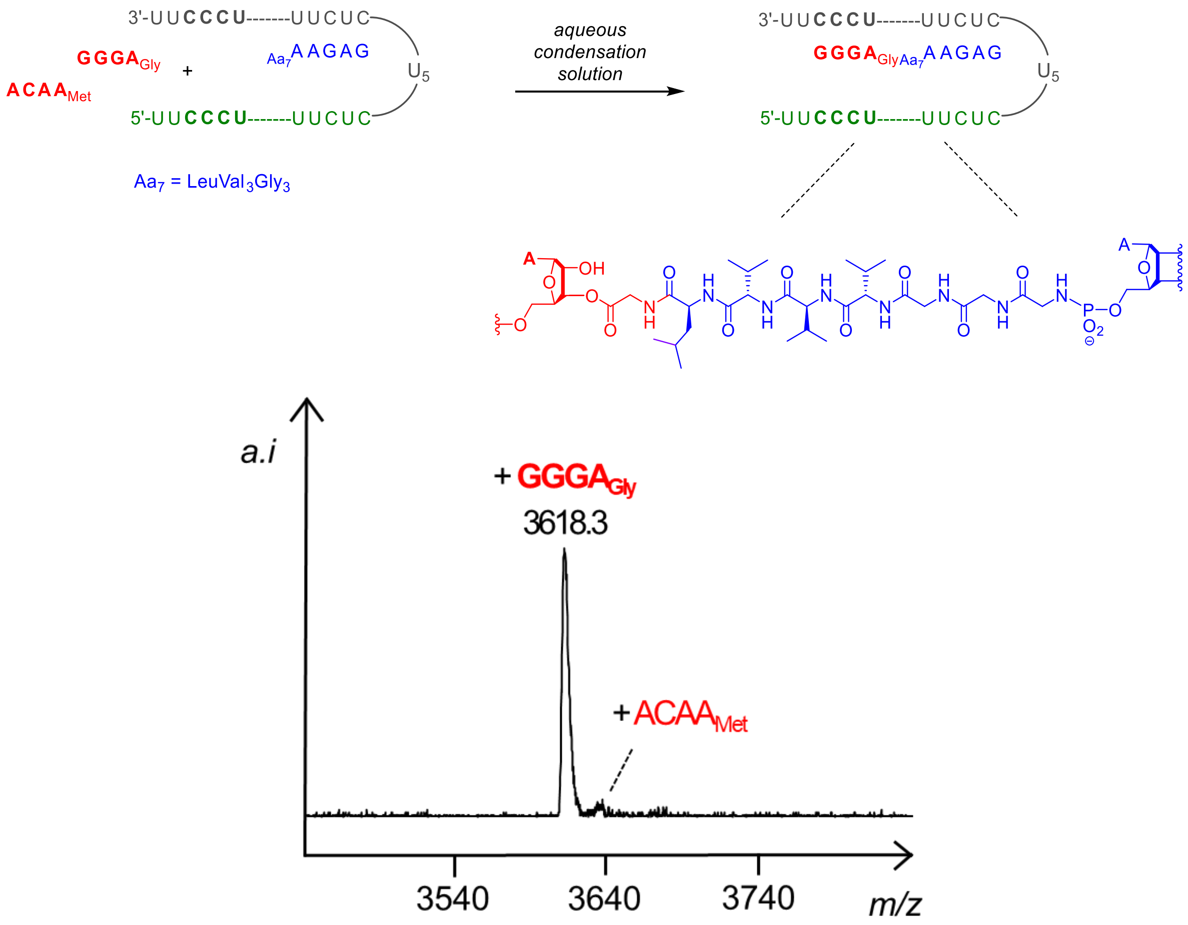

Figure S66. Reaction scheme and MALDI-TOF mass spectrum from translation assay with 20 µM LeuVal$_3$Gly$_3$-AAGAG, 20 µM template **1a**, 60 µM GGGA-Gly, 60 µM ACAA-Met, 800 µM CMP, 0.1 M $MgCl_2$, 0.2 M EDC · HCl, 1 mM phosphate buffer pH 6, 0 °C.

Table S14. Fidelity of translation with two aminoacylated transfer tetramers, peptido RNA LeuVal$_3$Gly$_3$-AAGAG and template **1a** as detected after 120 h.[a]

Product	Conversion (%)	$[M-H]^-$ m/z calc.	$[M-H]^-$ m/z found
GGGA-GlyLeuVal$_3$Gly$_3$-AAGAG	78	3614.1	3618.3
ACAA-MetLeuVal$_3$Gly$_3$-AAGAG	3	3632.7	3634.1

[a] Conditions for the assay are provided in the legend to Figure S66.

7. Additional Data from Translation Study

Effect of template overhangs and additives on hexapeptidoyl RNA formation

Spectra for data underlying Table 1.

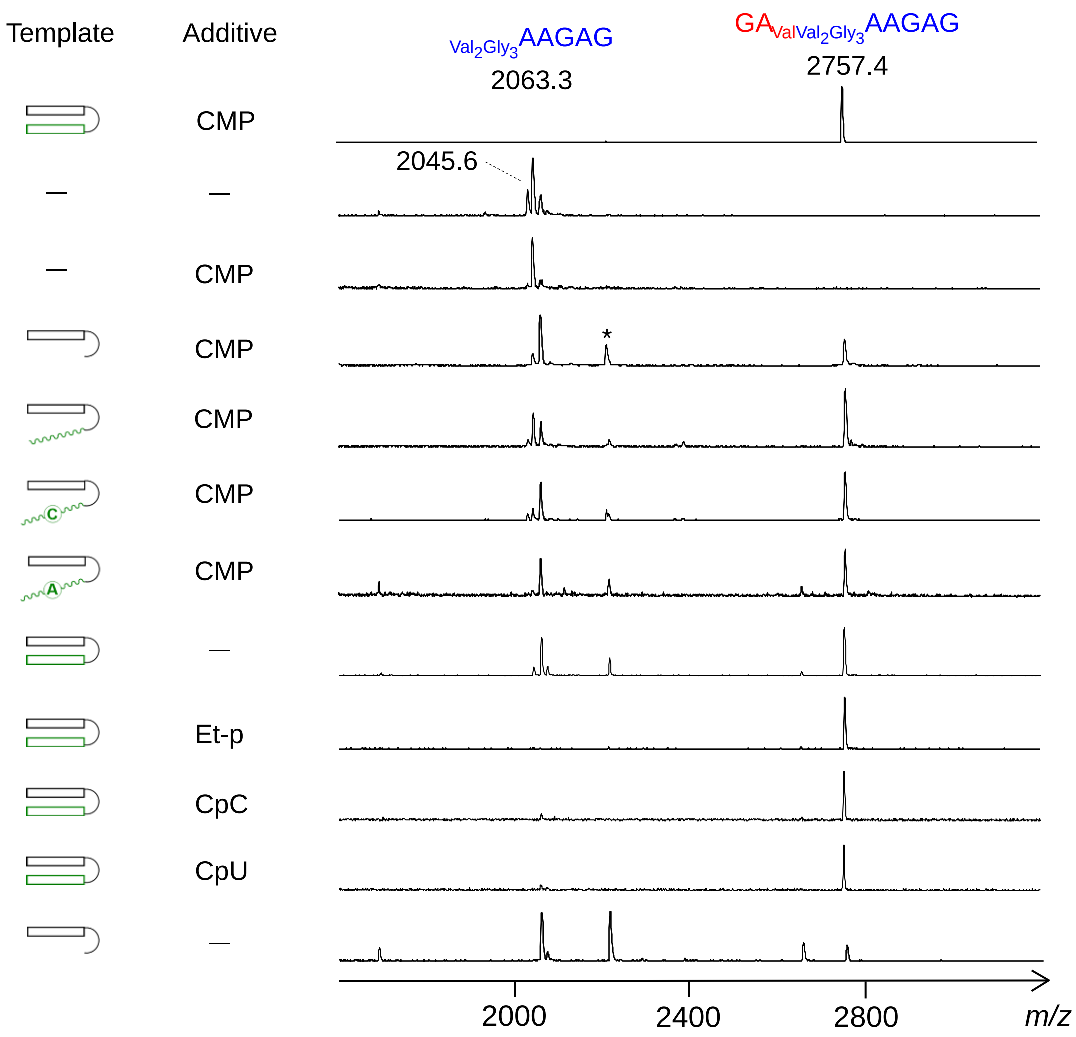

Figure S67. Comparison of representative MALDI-TOF mass spectra from translation assays with different templates in the presence or absence of organophosphate additives, measured after 96 h. These are spectra corresponding to the entries of Table 1 in the main manuscript. The signal at m/z = 2045.6 is caused by a dehydration product of Val_2Gly_3-AAGAG, which manifests itself when the desired translation is slow. The asterisk is for an EDC adduct. Conditions: 20 µM Val_2Gly_3-AAGAG, 20 µM template **1a-1e**, 400 µM GA-Val, 800 µM additive (CMP, Et-p. CpC, or CpU), 0.1 M $MgCl_2$, 0.2 M EDC · HCl, 1 mM phosphate buffer pH 6 at 0 °C.

Effect of triplex overhang on hexapeptidoyl RNA formation at 10-fold dilution

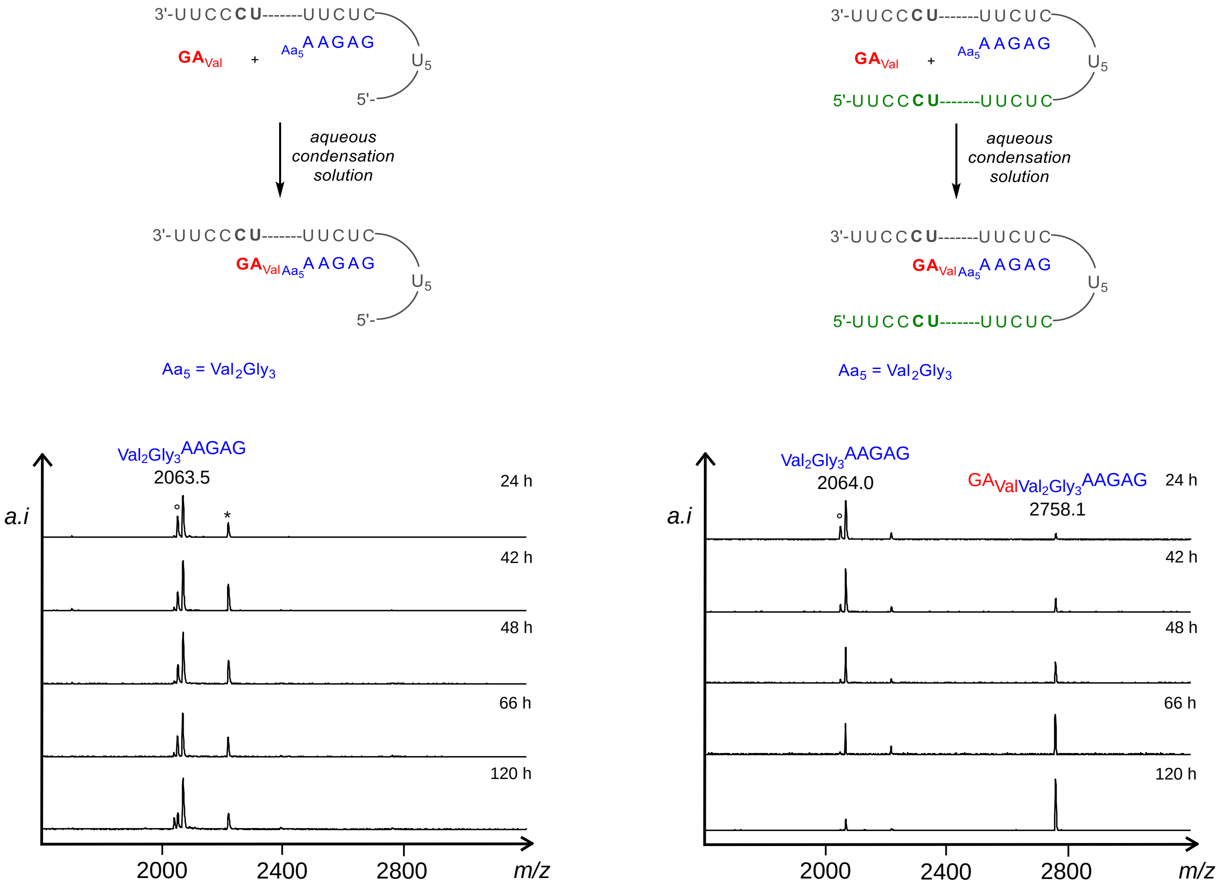

Figure S68. Comparison of representative MALDI-TOF mass spectra from translation assays at 10-fold dilution concentration with template **1a** and **1e**, in the presence of CMP as organophosphate additive. The peak labeled with a circle is for a mass 18 Da lower than that of the peptido RNA educt and most probably caused by dehydration. The asterisk is for an EDC adduct. Conditions: 20 µM Val_2Gly_3-AAGAG, 20 µM template **1a** or **1e**, 400 µM GA-Val, 800 µM CMP, 0.1 M $MgCl_2$, 0.2 M EDC · HCl, 1 mM phosphate buffer pH 6 at 0 °C.

Additional control experiment demonstrating successful translation in the 'Template Walk' regime of Single-Nucleotide Translation: Formation of octapeptidoyl RNA

To compare the 'template walk' to the 'tRNA growth' regime of read-out, an assay was conducted to examine the formation of octapeptidoyl RNA on template **1g**. The corresponding result for the 'tRNA growth' regime is shown in Figure S61, above.

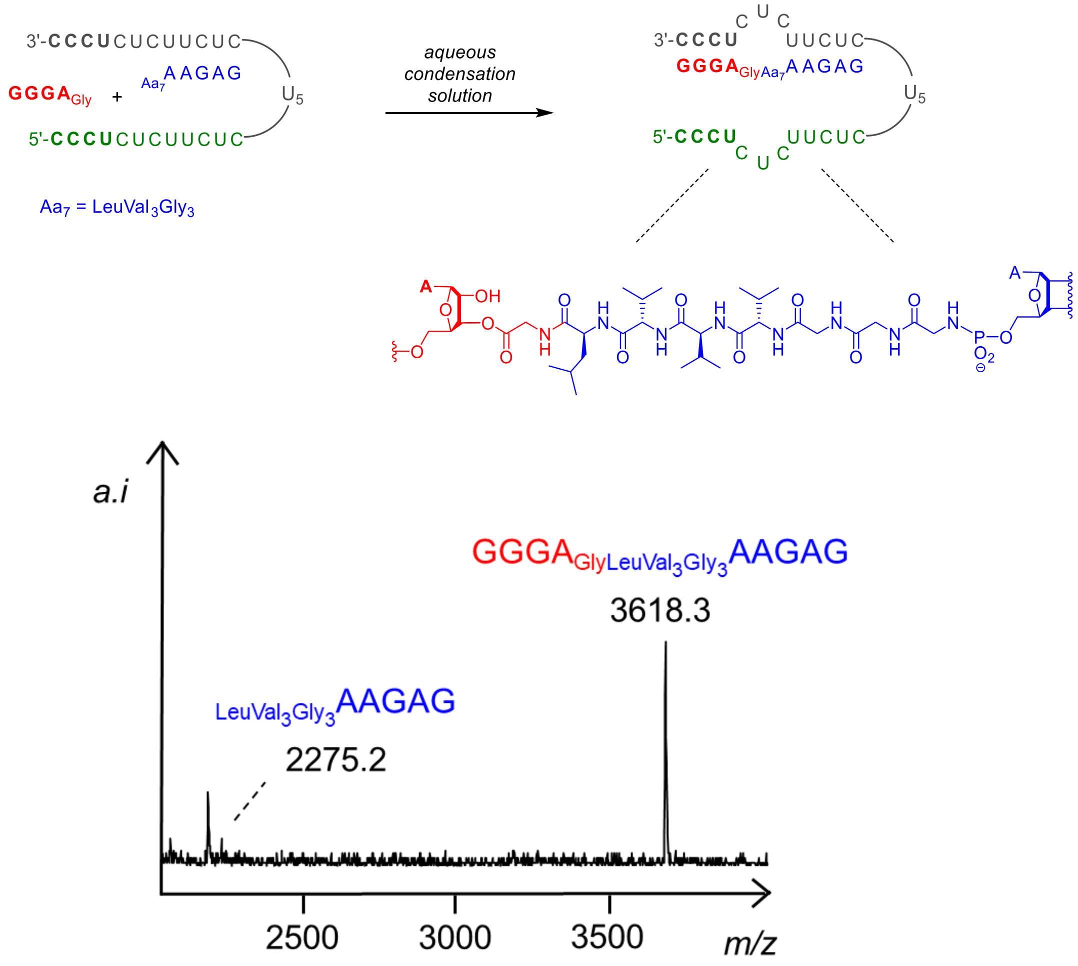

Figure S69. Reaction scheme and MALDI-TOF mass spectrum from translation assay with 20 µM LeuVal$_3$Gly$_3$-AAGAG, 20 µM template **1g**, 60 µM GGGA-Gly, 800 µM CMP, 0.1 M $MgCl_2$, 0.2 M EDC · HCl, 1 mM phosphate buffer pH 6, 0 °C after 72 h.

Again, the control experiment confirmed that either regime reads out the template sequence.

Release of octapeptide

Single nucleotide translation produced peptidoyl RNA. The free peptide can be released from this hydrolytically. This is described below.

A coupling assay was performed to form octapeptidoyl RNA (GGGA-GlyLeuVal$_3$Gly$_3$-AAGAG). The assay ran for 72 h at 0 °C (compare Figure S61). Then, the assay solution (1 µL) was diluted with water (1.7 µL), and NaOH solution (1 M, 0.3 µL) was added to give a final pH of 9. The basic solution was incubated at 0 °C for 20 h yielding the peptido RNA product of Figure S70a. Then, an aliquot of the assay solution (1 µL) was mixed with glacial acetic acid (AcOH, 17.5 M, 1 µL) and incubated at 0 °C for 4 d, leading to the spectrum of Figure S70b. Hydrolysis was confirmed by MALDI-TOF MS.

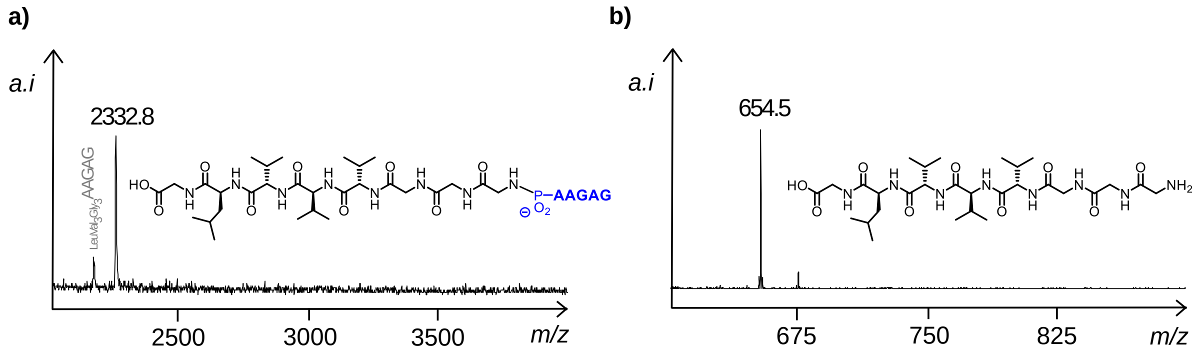

Figure S70. Release of GlyLeuVal$_3$Gly$_3$. a) MALDI-TOF mass spectrum from basic hydrolysis (pH 9) after 20 h, showing the formation of octapeptido RNA (GlyLeuVal$_3$Gly$_3$-AAGAG). b) MALDI-TOF mass spectrum of the free octapeptide after 4 d in water/acetic acid.

Release of peptidyl RNA from translation products

Besides the release of peptido RNA by ester hydrolysis, we also studied the cleavage of the phosphoramidate of heptapeptidoyl RNA GGA-GlyVal$_3$Gly$_3$-AAGAG to demonstrate that peptidyl RNA can be released from the peptidoyl RNA products of our translation reactions. This is shown in Figure S70, below.

The translation reaction was allowed to proceed for 2 d, as shown in Figure S71a, with MS measurement 5 min after start of the assay, and Figure S71b, at the end of the reaction time. The product was then isolated via C18 cartridge, and the product solution (1.5 µL) was mixed with formic acid solution (1 M, 0.3 µL, pH 3), and water (1.2 µL). The resulting solution was incubated for 24 h at 20 °C. The clean hydrolysis of the phosphoramidate is documented in Figure S71c, resulting in the formation of peptidyl RNA GGA-GlyVal$_3$Gly$_3$.

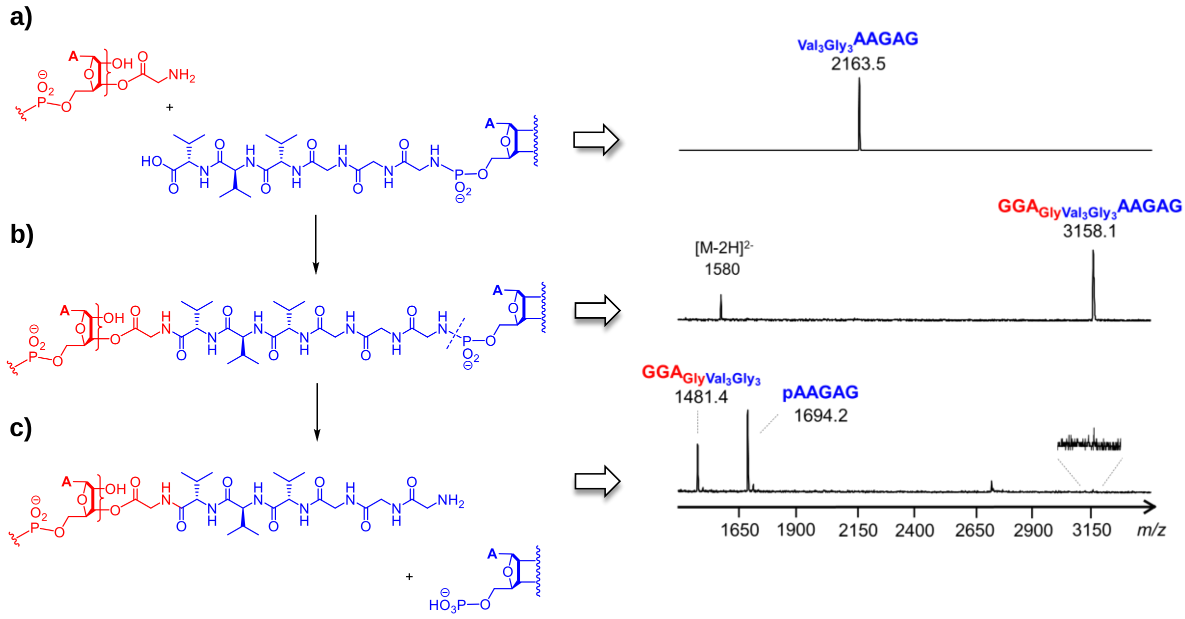

Figure S71. Set of MALDI-TOF mass spectra documenting translation with subsequent release of peptidyl RNA. a) Translation mixture after 5 min reaction time, for a mixture containing 20 µM Val$_3$Gly$_3$-AAGAG, 20 µM template **1a**, 120 µM GGA-Gly, 800 µM CMP, 0.1 M $MgCl_2$, 0.2 M EDC, 1 mM phosphate buffer pH 6, 0 °C. b) Purified peptidoyl RNA product, as obtained after 2 d reaction time. c) Spectrum of peptidyl RNA and primer RNA, as obtained after exposure to 0.1 M formic acid for 1 d at 20 °C. The sequences of the species detected are given above each peak. The expansion at m/z 3158 in part c) demonstrates how little peptidoyl RNA is left after 24 h acidic hydrolysis.

8. Systems Chemistry Study on Mixed Anhydride Formation

The model system of Figure S72 was studied to shed light on the activation and coupling reactions occurring in the presence of organophosphates. The amino acidyl nucleotide Gly-A was allowed to react with glycine methyl ester (H-Gly-OMe) as a model of aminoacylated tRNAs in the EDC-containing reaction buffer of our translation assays. Ethyl phosphate (Et-p) was added to mimic the free nucleotide CMP or template overhang. The rate constants (k) for the individual steps were determined in a series of NMR-monitored reactions of increasing complexity, as previously described for other reaction systems.[S10, S11] The flow of the substrates through the competing reaction channels was then elucidated by integrating over suitable reaction times, based on coupled differential equations. Further details of the study are presented below.

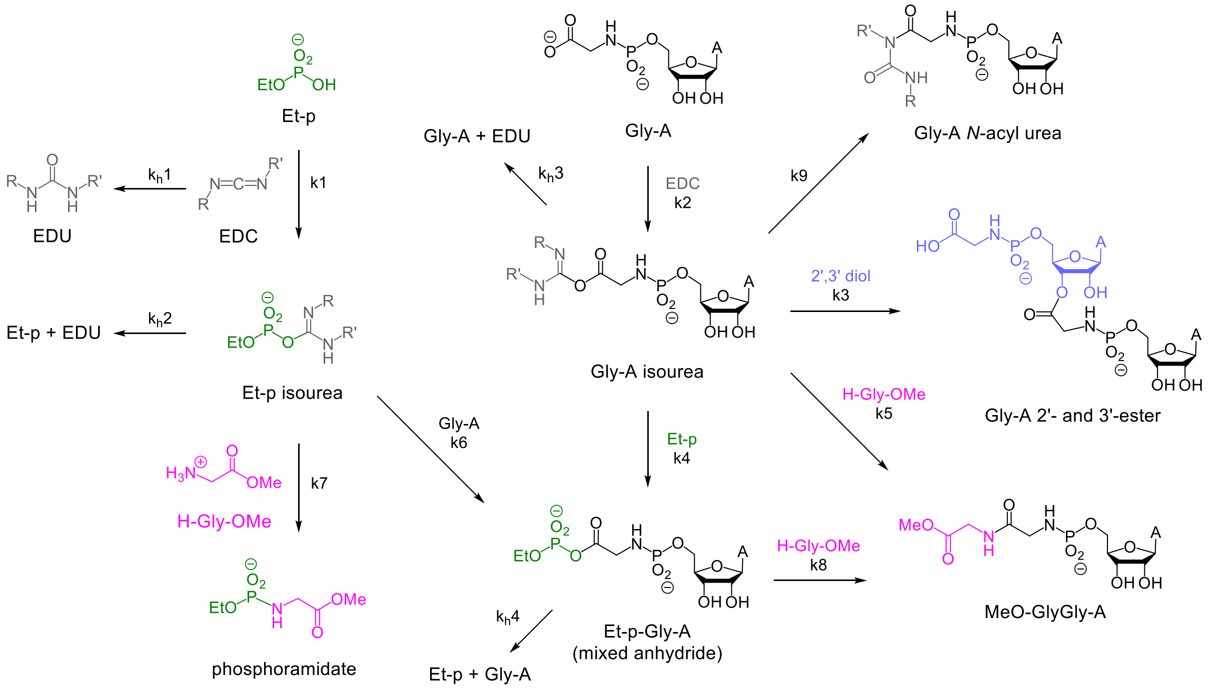

Figure S72. Reaction scheme showing the chemical transformations studied in the NMR-monitored assays.

NMR Methods

For kinetic measurements, the following experimental parameters were applied. All measurements were performed on a Bruker Avance 500 MHz spectrometer that was precooled to 4 °C. After insertion of the sample, it was allowed to stabilize the probe temperature for at least 10 minutes before the first measurement was started. The ^{1}H NMR spectra were recorded with 24 scans and a recycle delay of 2 s. The ^{13}C NMR spectra were recorded with proton decoupling with up to 1024 scans, depending on the experiment, and an recycle delay of 4 s. The ^{31}P NMR spectra were recorded with proton decoupling with up to 512 scans and an recycle delay of 3 s. Recycle delays for heteronuclei were chosen so that they would be at least 5 T_1 of the respective nucleus. The T_1 relaxation times were determined using the inversion recovery method. Under assay conditions (T = 4 °C, pH = 6.0, with 100 mM $MgCl_2$ and 0.4 mM Gd[DTPA], the relaxation time T_1 of ^{13}C-labeled Gly-A was determined to be 0.6 s in the ^{13}C channel and 0.4 s in the ^{31}P channel.

Assay with just EDC in MES buffer (NMR Assay 1). Aliquots of aqueous $MgCl_2$ solution (1 M, 50 µL) and MES solution (1 M, 50 µL) were added to D_2O (50 µL) and water (315 µL). The pH of the resulting solution was adjusted to a value of 6.0, using NaOH (2 M). Water was added to give an assay volume of 475 µL. The mixture was cooled to 0 °C. Then, a solution of EDC (1 M, 25 µL) was added, resulting in final concentrations of 100 mM $MgCl_2$, 100 mM MES and 50 mM EDC. The mixture was vortexed, transferred to an NMR tube and kept at 4 °C. The concentrations of EDC and EDU were monitored via ^{1}H NMR spectroscopy over a time span of 13 d.

Assay with EDC and Gly-A (NMR Assay 2). Aliquots of aqueous $MgCl_2$ solution (1 M, 50 µL), MES solution (1 M, 50 µL), Gd[DTPA] solution (20 mM, 10 µL) and ^{13}C-labeled Gly-A solution (50 mM, 150 µL) were added to D_2O (50 µL) and water (140 µL). The pH of the resulting solution was adjusted to a value of 6.0 using NaOH (2 M). Water was added to give an assay volume of 455 µL. The mixture was cooled to 0 °C. Then, a solution of EDC (1 M, 45 µL) was added, resulting in final concentrations of 100 mM $MgCl_2$, 100 mM MES, 0.4 mM Gd[DTPA], 15 mM ^{13}C-labeled Gly-A and 90 mM EDC. The mixture was vortexed, transferred to an NMR tube and kept at 4 °C. The concentrations of EDC and EDU were monitored via ^{1}H

NMR spectroscopy. The concentrations of Gly-A, Gly-A isourea and Gly-A ester and Gly-A *N*-acyl urea were measured via ^{13}C NMR spectroscopy.

Assay with EDC and ethyl phosphate (NMR Assay 3). Aliquots of aqueous $MgCl_2$ solution (1 M, 50 µL), MES solution (1 M, 50 µL), Gd[DTPA] solution (20 mM, 10 µL) and ethylphosphate solution (1 M, 10 µL) were added to D_2O (50 µL) and water (300 µL). The pH of the resulting solution was adjusted to a value of to 6.0 using NaOH (2 M). Water was added to give an assay volume of 475 µL. The mixture was cooled to 0 °C. Then, a solution of EDC (1 M, 25 µL) was added, resulting in final concentrations of 100 mM $MgCl_2$, 100 mM MES, 0.4 mM Gd[DTPA], 20 mM ethyl phosphate and 50 mM EDC. The mixture was vortexed, transferred to an NMR and kept at 4 °C. The concentrations of EDC and EDU were obtained from ^{1}H NMR signal intensities. The concentrations of ethyl phosphate and its isourea were obtained via ^{31}P NMR.

Assay with EDC, Gly-A and glycine methyl ester (NMR Assay 4). Aliquots of aqueous $MgCl_2$ solution (1 M, 50 µL), MES solution (1 M, 50 µL), Gd[DTPA] solution (20 mM, 10 µL), Gly-A solution (40 mM, 125 µL) and ^{13}C-labeled glycine methyl ester solution (0.2 M, 50 µL) were added to D_2O (50 µL) and water (130 µL). The pH of the resulting solution was adjusted to a value of to 6.0 using NaOH (2 M). Water was added to give an assay volume of 475 µL. The mixture was cooled to 0 °C. Then a solution of EDC (1 M, 25 µL) was added, resulting in final concentrations of 100 mM $MgCl_2$, 100 mM MES, 0.4 mM Gd[DTPA], 10 mM Gly-A, 20 mM glycine methyl ester and 50 mM EDC. The mixture was vortexed, transferred to an NMR tube and kept at 4 °C. The conversion of EDC to EDU and the EDC-derived intermediates was detected via ^{1}H NMR spectroscopy. The concentrations of glycine methyl ester and MeO-GlyGly-A were obtained from ^{13}C NMR spectra.

Assay with EDC, Gly-A and ethyl phosphate (NMR Assay 5). Aliquots of aqueous $MgCl_2$ solution (1 M, 50 µL), MES solution (1 M, 50 µL), Gd[DTPA] solution (20 mM, 10 µL), ethyl phosphate solution (1 M, 10 µL) and ^{13}C-labeled Gly-A solution (50 mM, 144 µL) were added to D_2O (50 µL) and water (140 µL). The pH of the resulting solution was adjusted to a value of 6.0 using NaOH (2 M). Water was added to give an assay volume of 450 µL. The mixture was cooled to 0 °C. Then a solution of EDC (1 M, 50 µL) was added, resulting in final concentrations of 100 mM $MgCl_2$, 100 mM MES, 0.4 mM Gd[DTPA], 20 mM ethyl phosphate, 14.4 mM ^{13}C-labeled Gly-A and 100 mM EDC. The mixture was vortexed, transferred to an

NMR tube and kept at 4 °C. The concentrations of EDC and EDU were monitored via ^{1}H NMR spectroscopy. The concentrations of Gly-A, Gly-A isourea, Gly-A ester, Et-p-Gly-A and Gly-A *N*-acyl urea were all obtained from ^{13}C NMR data. The concentrations of ethyl phosphate and ethyl phosphate isourea were monitored via ^{31}P NMR spectroscopy.

Assay with EDC, ethyl phosphate and glycine methyl ester (NMR assay 6). Aliquots of aqueous $MgCl_2$ solution (1 M, 50 µL), MES solution (1 M, 50 µL), Gd[DTPA] solution (20 mM, 10 µL), ethyl phosphate solution (1 M, 8 µL) and ^{13}C-labeled glycine methyl ester solution (0.2 M, 50 µL) were added to D_2O (50 µL) and water (240 µL). The pH of the resulting solution was adjusted to a value of 6.0 using NaOH (2 M). Water was added to give an assay volume of 475 µL. The mixture was cooled to 0 °C. Then a solution of EDC (1 M, 25 µL) was added, resulting in final concentrations of 100 mM $MgCl_2$, 100 mM MES, 0.4 mM Gd[DTPA], 16 mM ethyl phosphate, 20 mM glycine methyl ester and 50 mM EDC. The mixture was vortexed, transferred to an NMR tube and kept at 4 °C. The concentrations of EDC and EDU were from ^{1}H NMR spectra. The concentrations of ethyl phosphate, ethyl phosphate isourea and the glycine methyl ester phosphoramidate were from via ^{31}P NMR spectra.

Assay with just mixed anhydride in MES buffer (NMR assay 7). Aliquots of aqueous $MgCl_2$ solution (1 M, 50 µL), MES solution (1 M, 50 µL), Gd[DTPA] solution (20 mM, 10 µL) were added to D_2O (50 µL) and water (330 µL). The pH of the resulting solution was adjusted to a value of 6.0 using NaOH (2 M). Water was added to give an assay volume of 500 µL and the mixture was cooled to 0 °C. A small amount of mixed anhydride was added as solid. The resulting mixture was vortexed, transferred to an NMR tube and kept at 4 °C. The final concentration of mixed anhydride in the assay solution was determined via UV-vis spectroscopy. It was found to be 8.5 mM, so that the final concentrations were 100 mM $MgCl_2$, 100 mM MES, 0.4 mM Gd[DTPA] and 8.5 mM Et-p-Gly-A. The concentrations of ethyl phosphate, Gly-A and Et-p-Gly-A were monitored via ^{31}P NMR spectroscopy.

Assay with mixed anhydride and glycine methyl ester (NMR assay 8). Aliquots of aqueous $MgCl_2$ solution (1 M, 50 µL), MES solution (1 M, 50 µL), Gd[DTPA] solution (20 mM, 10 µL) ^{13}C-labeled glycine methyl ester solution (0.2 M, 50 µL) were added to D_2O (50 µL) and water (280 µL). The pH of the resulting solution was adjusted to a value of to 6.0 using NaOH (2 M). Water was added to give an assay volume of 500 µL and the mixture was cooled to 0 °C. A small amount of mixed anhydride was added as solid. The resulting mixture was vortexed, transferred to an NMR tube and kept at 4 °C. The final concentration of mixed

anhydride in the assay solution was determined via UV-vis spectroscopy. It was found to be 6.5 mM, resulting in final concentrations of 100 mM $MgCl_2$, 100 mM MES, 0.4 mM Gd[DTPA], 20 mM glycine methyl ester and 6.5 mM Et-p-Gly-A (mixed anhydride). The concentrations of ethyl phosphate, Gly-A, Et-p-Gly-A and MeO-GlyGly-A were obtained by ^{31}P NMR spectroscopy.

Assay with EDC, Gly-A, ethyl phosphate and glycine methyl ester (NMR assay 9). Aliquots of aqueous $MgCl_2$ solution (1 M, 50 μL), MES solution (1 M, 50 μL), Gd[DTPA] solution (20 mM, 10 μL), ethyl phosphate solution (1 M, 10 μL), Gly-A solution (40 mM, 125 μL) and ^{13}C-labeled glycine methyl ester solution (0.2 M, 50 μL) were added to D_2O (50 μL) and water (130 μL). The pH of the resulting solution was adjusted to a value of 6.0 using NaOH (2 M). Water was added to give an assay volume of 475 μL. The mixture was cooled to 0 °C. Then, a solution of EDC (1 M, 25 μL) was added, resulting in final concentrations of 100 mM $MgCl_2$, 100 mM MES, 0.4 mM Gd[DTPA], 20 mM ethyl phosphate, 10 mM Gly-A, 20 mM glycine methyl ester and 50 mM EDC. The mixture was vortexed, transferred to an NMR tube and kept at 4 °C. The concentrations of EDC and EDU were monitored by ^{1}H NMR spectroscopy. The concentrations of glycine methyl ester, MeO-GlyGly-A and the glycinyl phosphoramidate were monitored via ^{13}C NMR spectroscopy.

NMR Signals Monitored

Table S15. Chemical shifts of signals used for quantification in NMR-monitored assays.

Observed nucleus	Chemical shift (ppm)	Multi-plicity	Compound	Structure (signal highlighted)
^{1}H	0.82	triplet	EDC	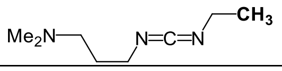
	0.75	triplet	EDU	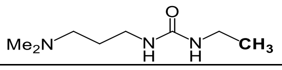
^{13}C (^{13}C-labeled Gly-A)	178.9	doublet	Gly-A	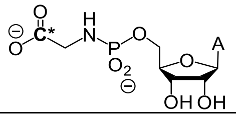
	176.7<br>175.5	triplet<br>triplet	Gly-A isourea<br>Gly-A isourea	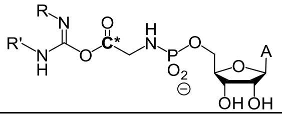
	174.5<br>174.2	doublet<br>doublet	Gly-A ester<br>Gly-A ester	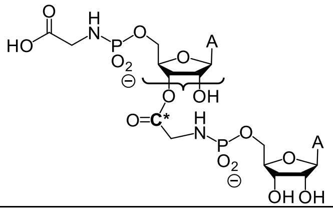
	170.7<br>170.5	doublet<br>doublet	Gly-A *N*-acyl urea<br>Gly-A *N*-acyl urea	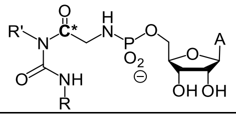
	169.9	doublet of doublets	mixed anhydride (Et-p-Gly-A)	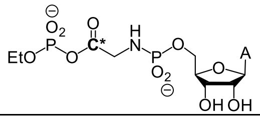
^{13}C (^{13}C-labeled H-Gly-OMe)	174.6	doublet	phosphoramidate	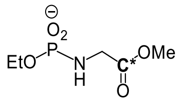
	171.6	singlet	MeO-GlyGly-A	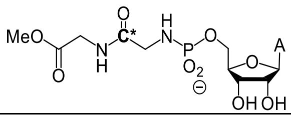
	168.6	singlet	H-Gly-OMe	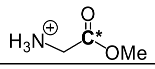
^{31}P	7.78	singlet	Gly-A	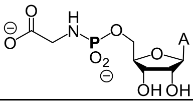
	7.45	singlet	MeO-GlyGly-A	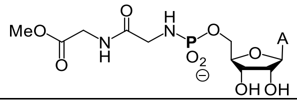
	7.42	singlet	phosphoramidate	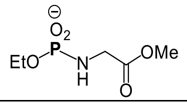

	6.92	singlet	mixed anhydride	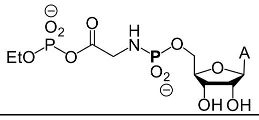
	2.00	singlet	ethyl phosphate	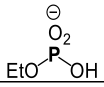
	-7.24	singlet	Et-p isourea	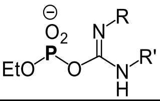
	-7.33	singlet	Et-p isourea	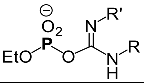
	-7.60	doublet	mixed anhydride	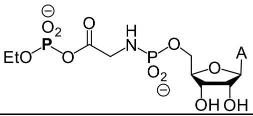

Mathematical model

Starting materials of the reaction system of Figure S72 can react through several reaction channels, often in more than one step. Because the transformations are connected through the network of molecular species, simulating it requires coupled differential equations. Shown below are the reactions considered, with the naming of the rate constant for each reaction, followed by the list of rate equations. The simulation was run in Mathematica, version 11.3, employing the NDSolve module.

ethyl phosphate + EDC $\xrightarrow{k1}$ ethyl phosphate isourea

GlyA + EDC $\xrightarrow{k2}$ GlyA isourea

GlyA isourea + diol $\xrightarrow{k3}$ GlyA ester + EDU

GlyA isourea + ethyl phosphate $\xrightarrow{k4}$ mixed anhydride + EDU

GlyA isourea + HGlyOMe $\xrightarrow{k5}$ dipeptide + EDU

ethyl phosphate isourea + GlyA $\xrightarrow{k6}$ mixed anhydride + EDU

ethyl phosphate isourea + HGlyOMe $\xrightarrow{k7}$ phosphoramidate + EDU

mixed anhydride + HGlyOMe $\xrightarrow{k8}$ dipeptide + ethyl phosphate

GlyA isourea $\xrightarrow{k9}$ GlyA *N*-acylurea

EDC $\xrightarrow{kh1}$ EDU

GlyA isourea $\xrightarrow{kh2}$ GlyA + EDU

ethyl phosphate isourea $\xrightarrow{kh3}$ ethyl phosphate + EDU

mixed anhydride $\xrightarrow{kh4}$ GlyA + ethyl phosphate

Rate equations

d[EDC]/dt = - kh1 EDC[t] - k1 EDC[t] ethylphosphate[t] - k2 EDC[t] GlyA[t]

d[EDU]/dt = - k3 GlyAisourea[t] diol[t] + k4 GlyAisourea[t] ethylphosphate[t] + k5 GlyAisourea[t] HGlyOMe[t] + k6 ethylphosphateisourea[t] GlyA[t] + k7 ethylphosphateisourea[t] HGlyOMe[t] + kh1 EDC[t] + kh2 ethylphosphateisourea[t] + kh3 GlyAisourea[t]

d[GlyA]/dt = - k2 EDC[t] GlyA[t] - k6 ethylphosphateisourea[t] GlyA[t] + kh3 GlyAisourea[t] + kh4 mixedanhydride[t]

d[ethylphosphate]/dt = - k1 EDC[t] ethylphosphate[t] - k4 GlyAisourea[t] ethylphosphate[t] + k8 mixedanhydride[t] HGlyOMe[t] + kh2 ethylphosphateisourea[t] + kh4 mixedanhydride[t]

d[diol]/dt = - k3 GlyAisourea[t] diol[t]

d[HGlyOMe]/dt = - k5 GlyAisourea[t] HGlyOMe[t] - k7 ethylphosphateisourea[t] HGlyOMe[t] - k8 mixedanhydride[t] HGlyOMe[t]

d[GlyAisourea]/dt = k2 EDC[t] GlyA[t] - k3 GlyAisourea[t] - k3 GlyAisourea[t] diol[t] - k4 GlyAisourea[t] ethylphosphate[t] - k5 GlyAisourea[t] HGlyOMe[t] - k9 GlyAisourea[t] - kh3 GlyAisourea[t]

d[ethylphosphateisourea]/dt = k1 EDC[t] ethylphosphate[t] -
k6 ethylphosphateisourea[t] GlyA[t] - k7 ethylphosphateisourea[t] HGlyOMe[t] -kh1 ethylphosphateisourea[t] - kh2 ethylphosphateisourea[t]

d[phosphoramidate]/dt = k7 ethylphosphateisourea[t] HGlyOMe[t]

d[EDUGlyA]/dt = k9 GlyAisourea[t]

d[GlyAEster]/dt = k3 GlyAisourea[t] diol[t]

d[dipeptide]/dt = k5 GlyAisourea[t] HGlyOMe[t] +k8 mixedanhydride[t] HGlyOMe[t]

d[mixedanhydride]/dt = k4 GlyAisourea[t] ethylphosphate[t] + k6 ethylphosphateisourea[t] GlyA[t] - k8 mixedanhydride[t] HGlyOMe[t] - kh4 mixedanhydride[t]

Spectra and Fits

NMR Assay 1

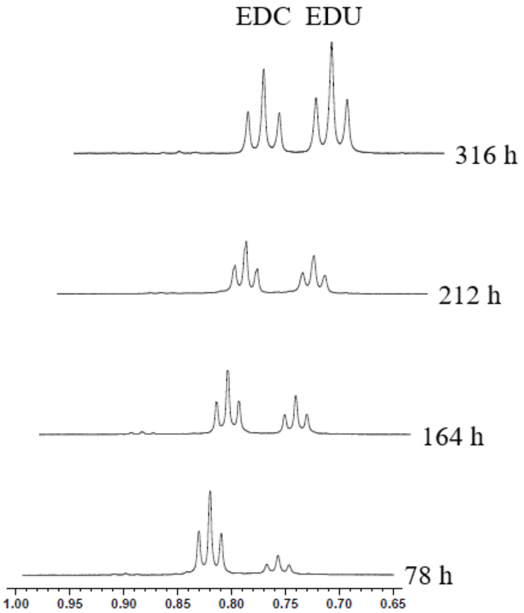

Figure S73. Stacked plot, showing ^{1}H signals for EDC/EDU observed in NMR Assay 1. Spectra were measured on a 700 MHz spectrometer with exception of the uppermost spectrum, which was measured on a 500 MHz spectrometer. Conditions: 100 mM $MgCl_2$, 100 mM MES and 50 mM EDC at 4 °C.

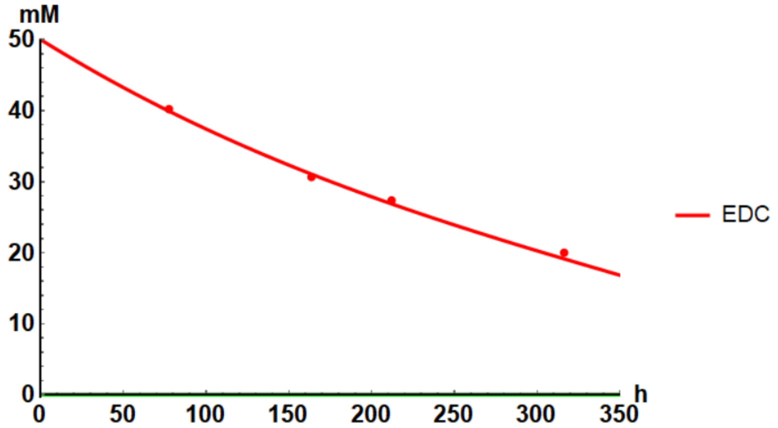

Figure S74. Kinetics of hydrolysis of EDC, as determined in NMR Assay 1; experimental data points and monoexponential fit.

NMR Assay 2

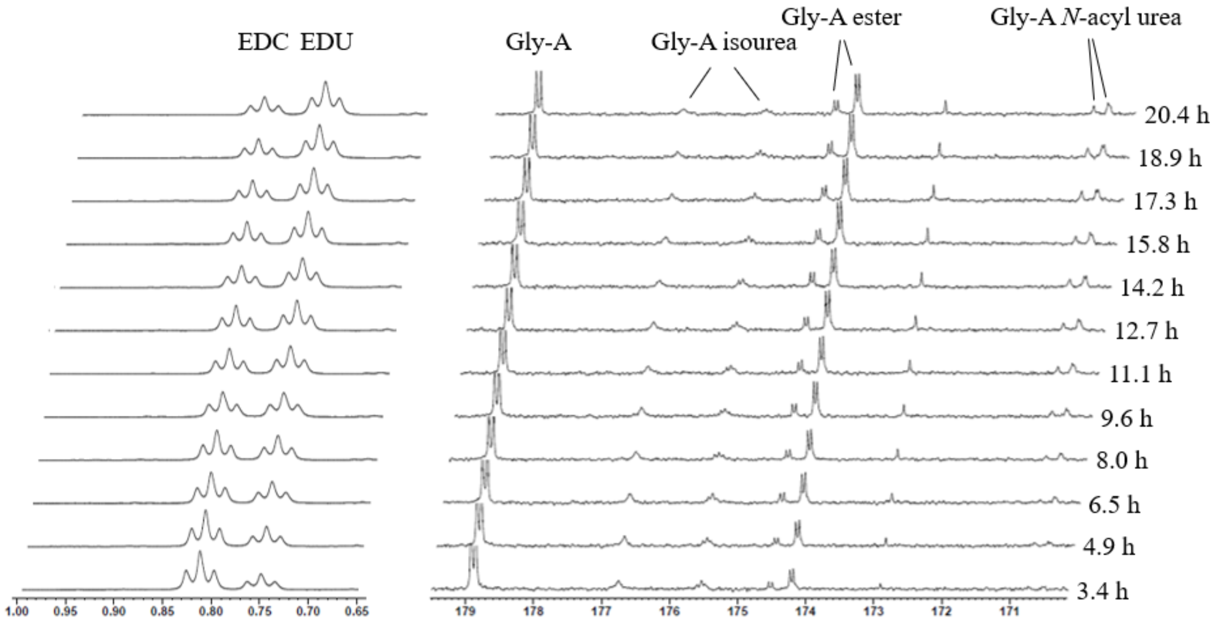

Figure S75. Stacked plots, showing the ^{1}H (left) and ^{13}C (right) signals observed in NMR Assay 2. Spectra were measured on a 500 MHz spectrometer. Conditions: 100 mM $MgCl_2$, 100 mM MES, 0.4 mM Gd[DTPA], 15 mM ^{13}C-labeled Gly-A and 90 mM EDC at 4 °C.

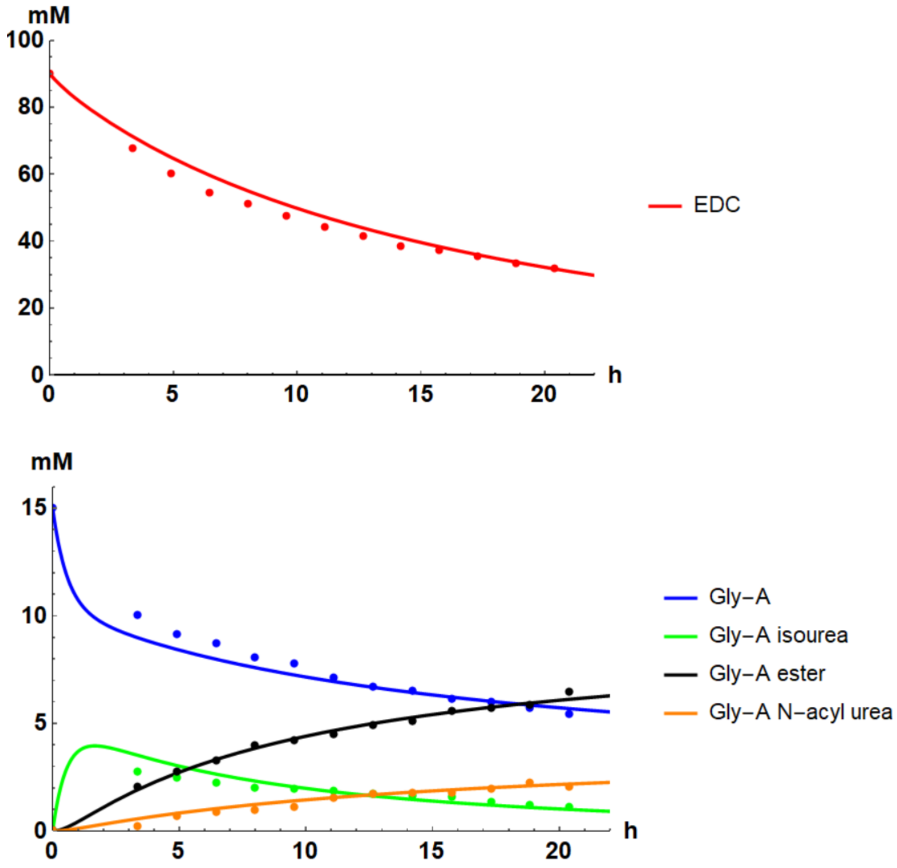

Figure S76. Kinetic plots for the reaction of EDC with Gly-A, as determined in NMR Assay 2; experimental data points and fits determined by numerically solving the coupled differential equations.

NMR Assay 3

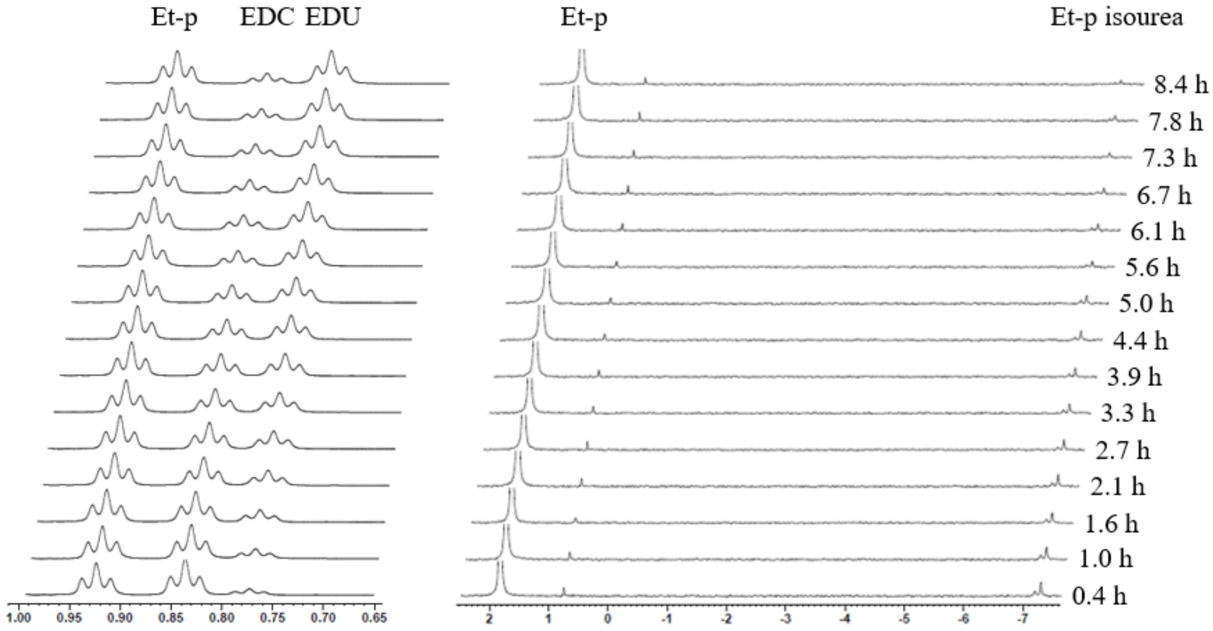

Figure S77. Stacked plots, showing the ^{1}H (left) and ^{31}P (right) signals observed in NMR Assay 3. Spectra were measured on a 500 MHz spectrometer. Conditions: 100 mM $MgCl_2$, 100 mM MES, 0.4 mM Gd[DTPA], 20 mM ethyl phosphate and 50 mM EDC at 4 °C.

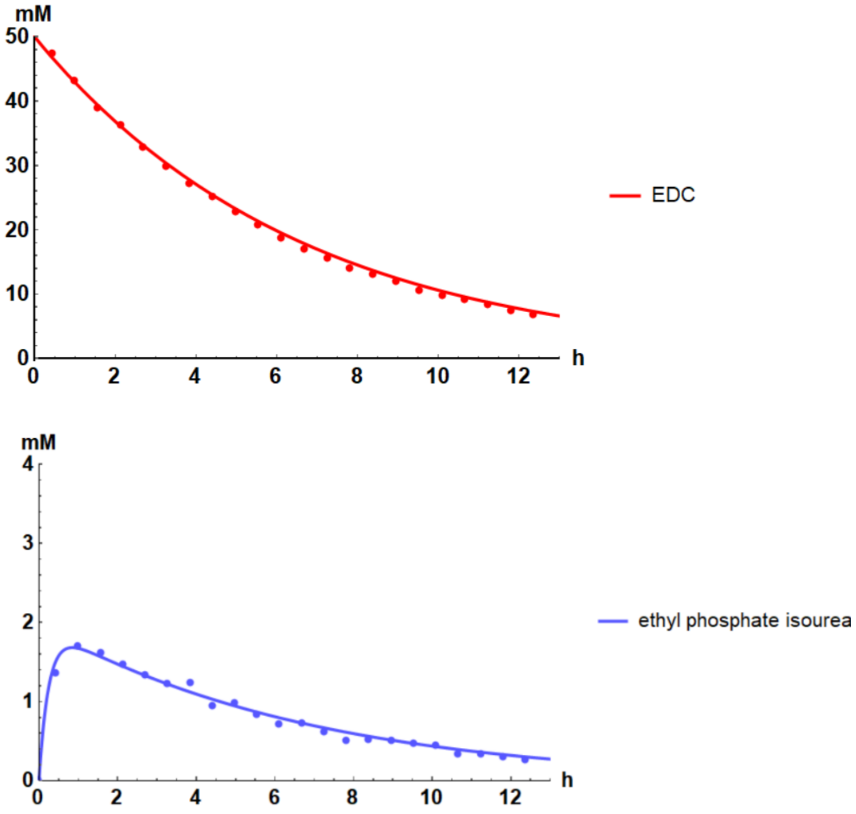

Figure S78. Kinetic plots for the reaction of EDC with Et-p, as determined in NMR Assay 3; experimental data points and fits determined by numerically solving the coupled differential equations.

NMR Assay 4

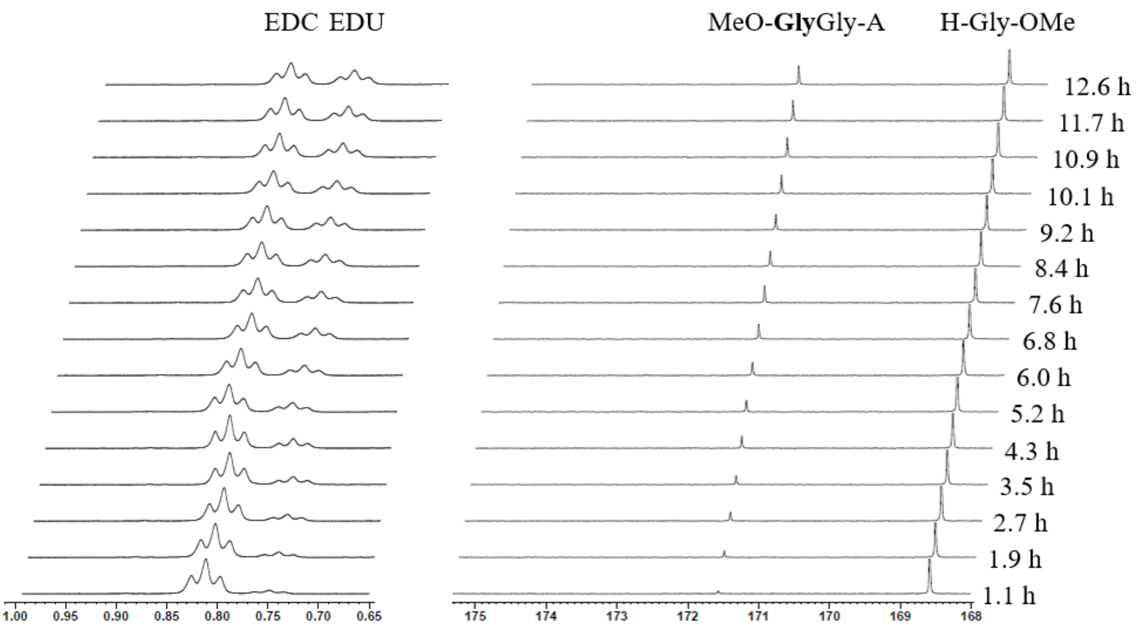

Figure S79. Stacked plots, showing the ^{1}H (left) and ^{13}C (right) signals observed in NMR Assay 4. Spectra were measured on a 500 MHz spectrometer. Conditions: 100 mM $MgCl_2$, 100 mM MES, 0.4 mM Gd[DTPA], 10 mM Gly-A, 20 mM glycine methyl ester and 50 mM EDC at 4 °C.

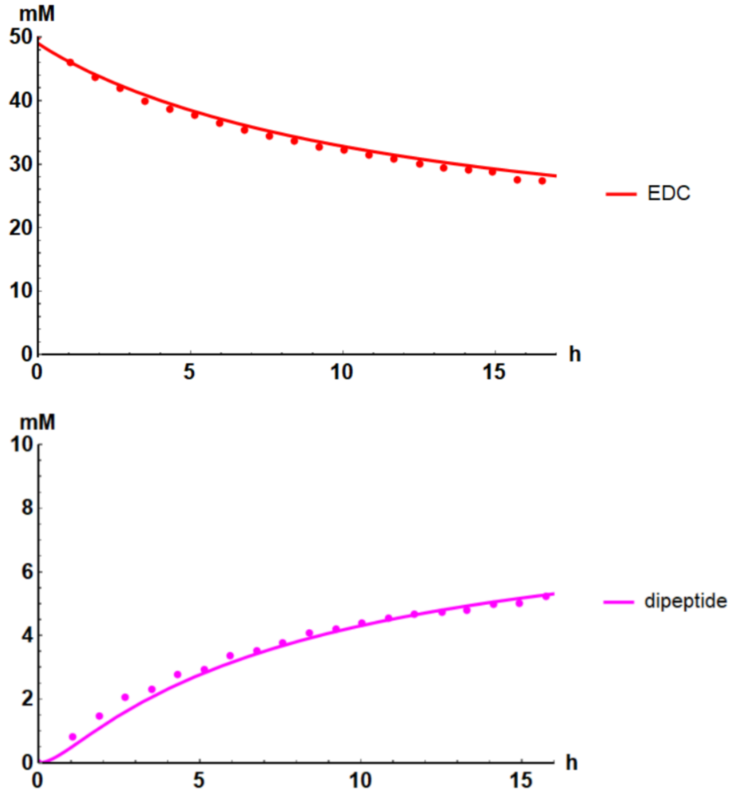

Figure S80. Kinetic plots for the reaction of Gly-A with glycine methyl ester and EDC, as determined in NMR Assay 4; experimental data points and fits determined by numerically solving the coupled differential equations.

NMR Assay 5

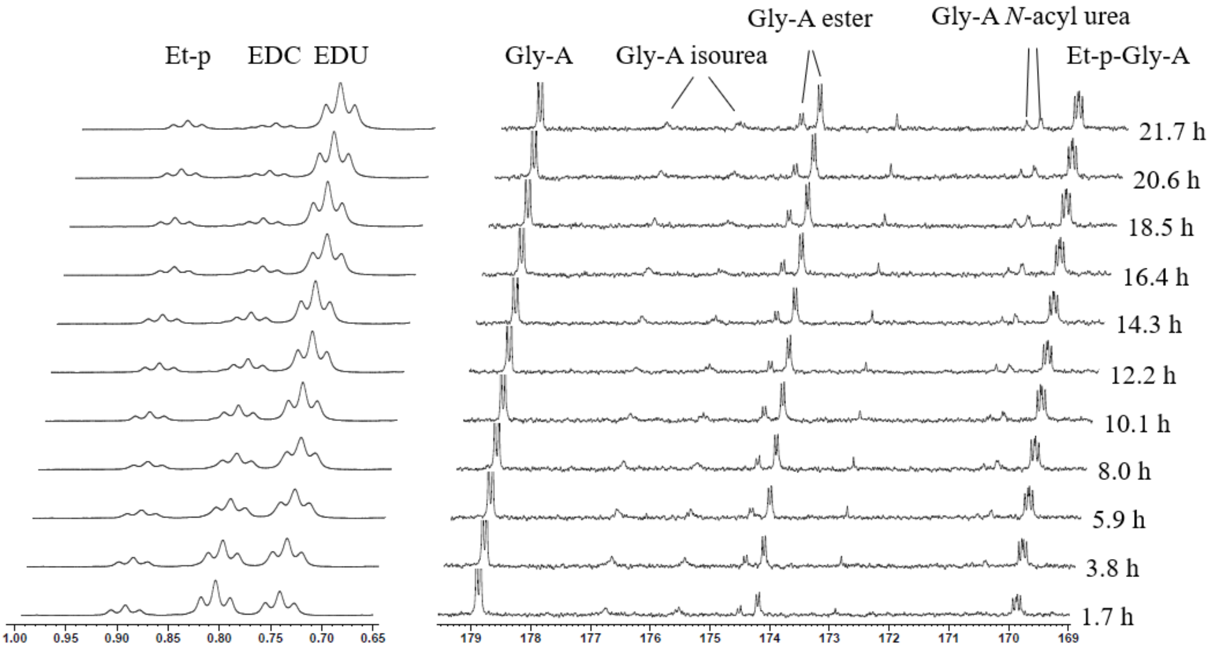

Figure S81. Stacked plots, showing the ^{1}H (left) and ^{13}C (right) signals observed in NMR Assay 5. Spectra were measured on a 500 MHz spectrometer. Conditions: 100 mM $MgCl_2$, 100 mM MES, 0.4 mM Gd[DTPA], 20 mM ethyl phosphate, 14.4 mM ^{13}C-labeled Gly-A and 100 mM EDC at 4 °C.

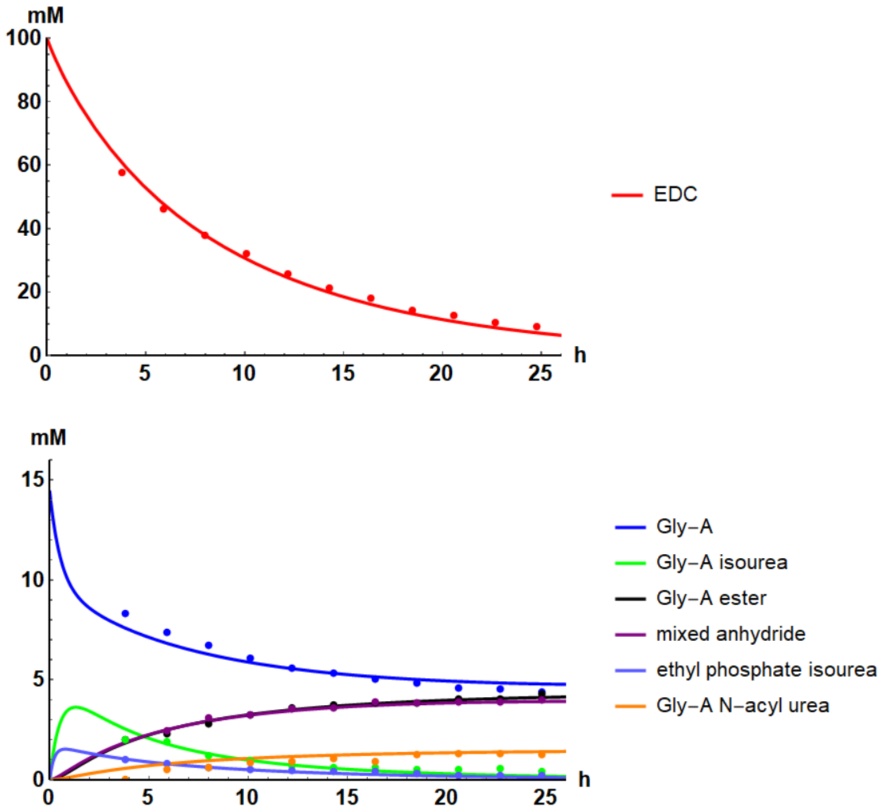

Figure S82. Kinetic plots for the reaction of Gly-A with Et-p and EDC, as determined in NMR Assay 5; experimental data points and fits determined by numerically solving the coupled differential equations.

NMR Assay 6

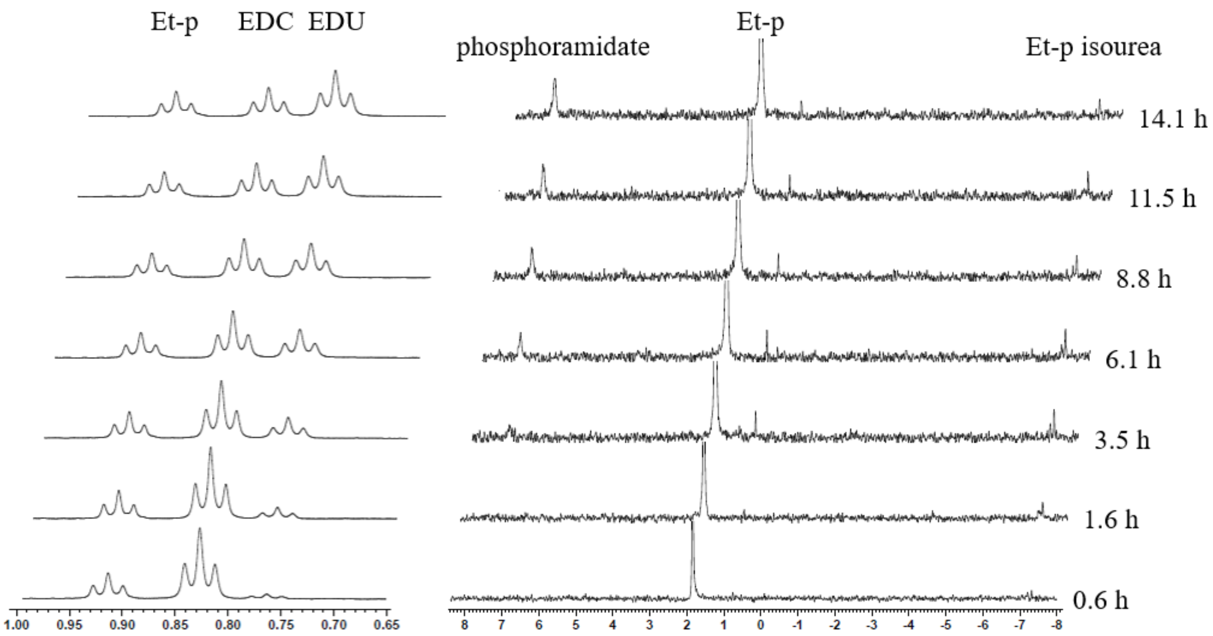

Figure S83. Stacked plots, showing the ^{1}H (left) and ^{31}P (right) signals in NMR Assay 6. Spectra were measured on a 500 MHz spectrometer. Conditions: 100 mM $MgCl_2$, 100 mM MES, 0.4 mM Gd[DTPA], 16 mM ethyl phosphate, 20 mM glycine methyl ester and 50 mM EDC at 4 °C.

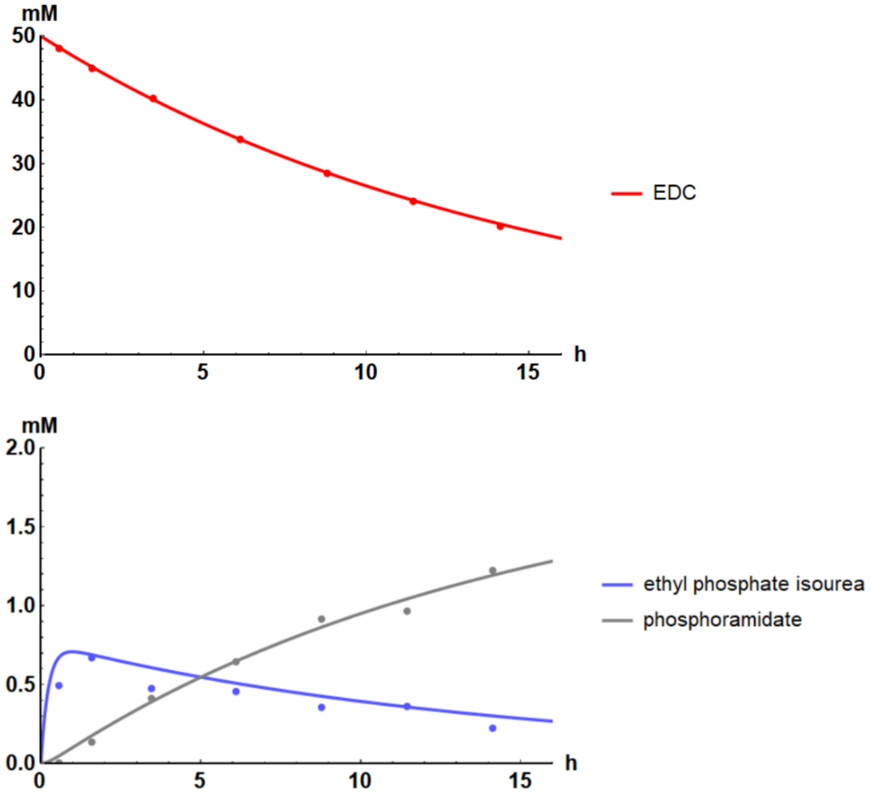

Figure S84. Kinetic plots for the reaction of glycine methyl ester with Et-p and EDC, as determined in NMR Assay 6; experimental data points and fits determined by numerically solving the coupled differential equations.

NMR Assay 7

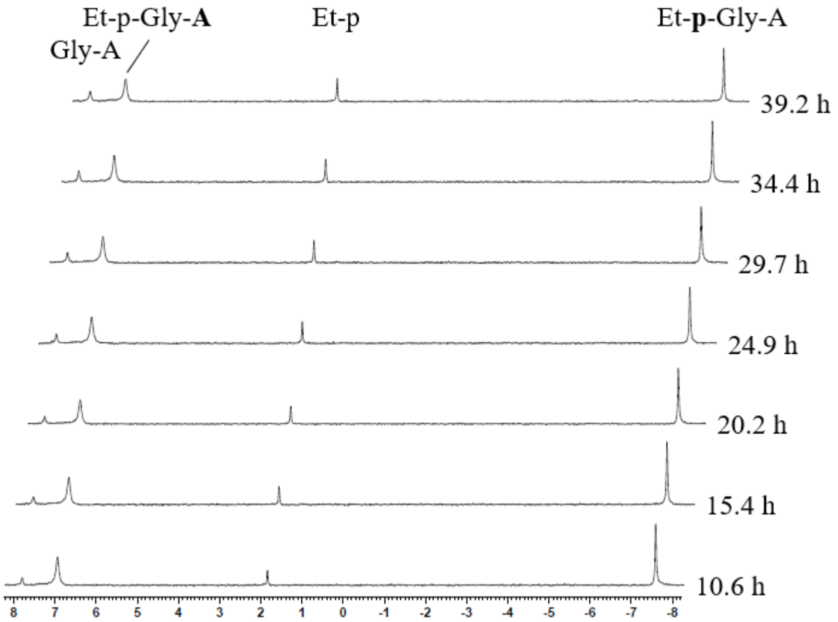

Figure S85. Stacked plot, showing the ^{31}P signals of the hydrolysis of Et-p-Gly-A observed in NMR Assay 7. Spectra were measured on a 500 MHz spectrometer. Conditions: 100 mM $MgCl_2$, 100 mM MES, 0.4 mM Gd[DTPA] and 8.5 mM Et-p-Gly-A at 4 °C.

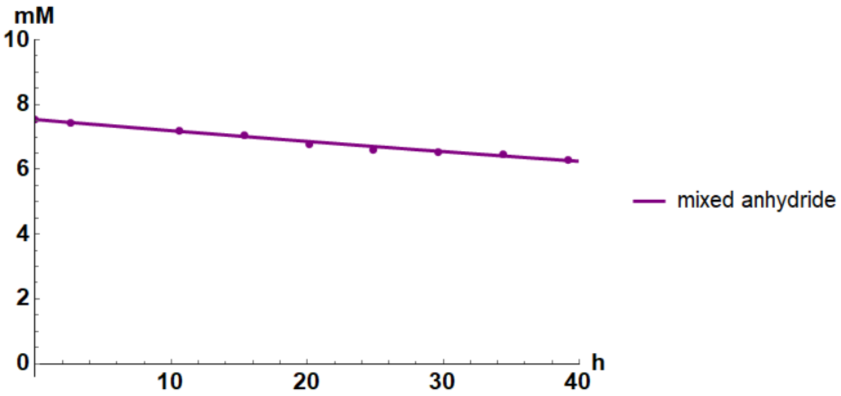

Figure S86. Kinetics of hydrolysis of Et-p-Gly-A, as determined in NMR Assay 7; experimental data points and monoexponential fit.

NMR Assay 8

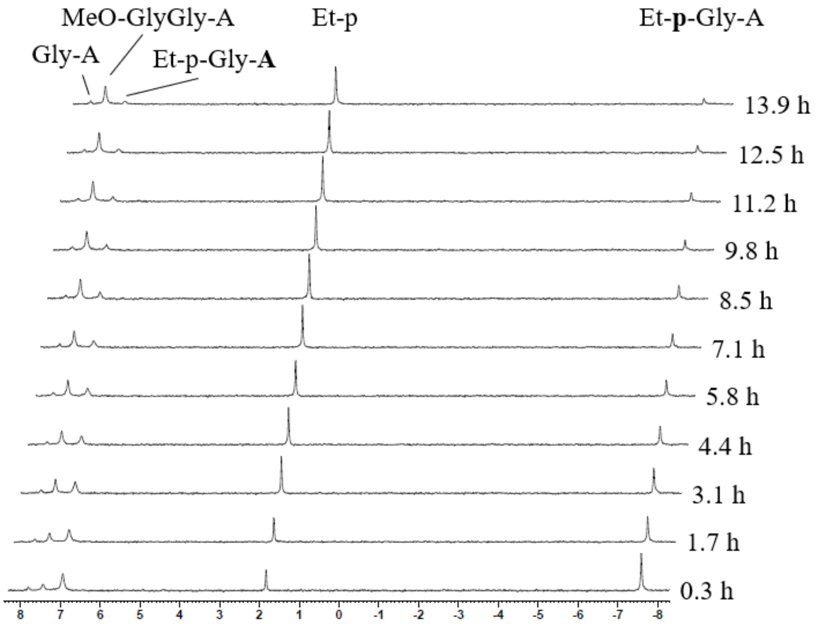

Figure S87. Stacked plot, showing the ^{31}P signals observed in NMR Assay 8. Spectra were measured on a 500 MHz spectrometer. Conditions: 100 mM $MgCl_2$, 100 mM MES, 0.4 mM Gd[DTPA], 20 mM glycine methyl ester and 6.5 mM Et-p-Gly-A at 4 °C.

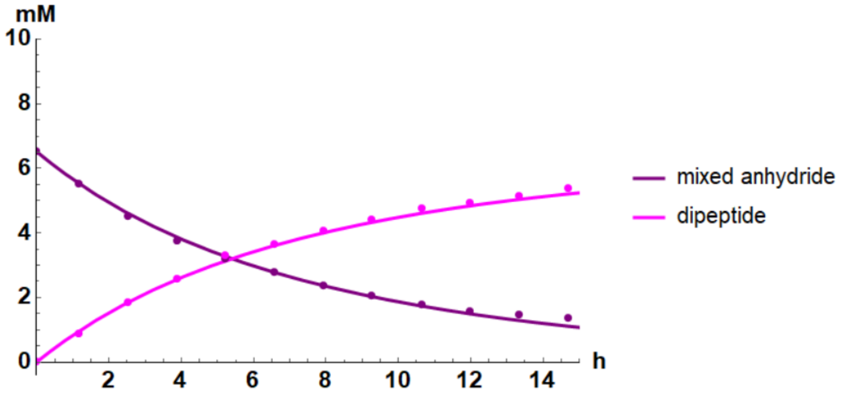

Figure S88. Kinetic plot for the reaction of glycine methyl ester with Et-p-Gly-A yielding dipeptide (MeO-GlyGly-A), as determined in NMR Assay 8; experimental data points and fits determined by numerically solving the coupled differential equations.

NMR Assay 9

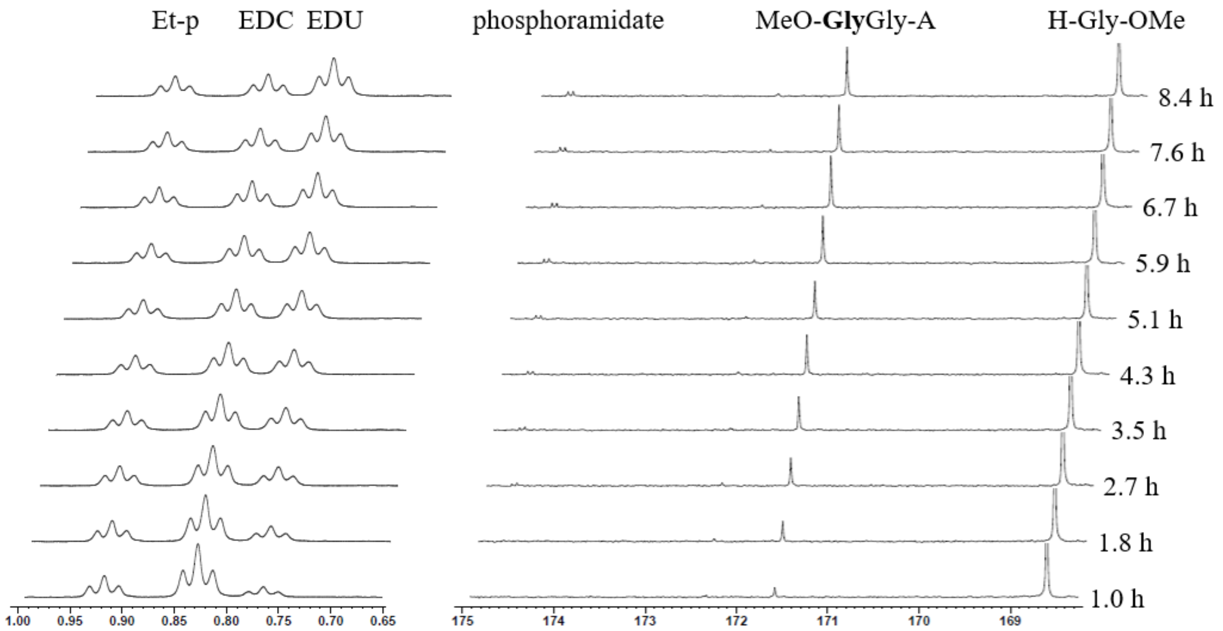

Figure S89. Stacked plots, showing the ^{1}H (left) and ^{13}C (right) signals in NMR Assay 9. Spectra were measured on a 500 MHz spectrometer. Conditions: 100 mM $MgCl_2$, 100 mM MES, 0.4 mM Gd[DTPA], 20 mM ethyl phosphate, 10 mM Gly-A, 20 mM glycine methyl ester and 50 mM EDC at 4 °C.

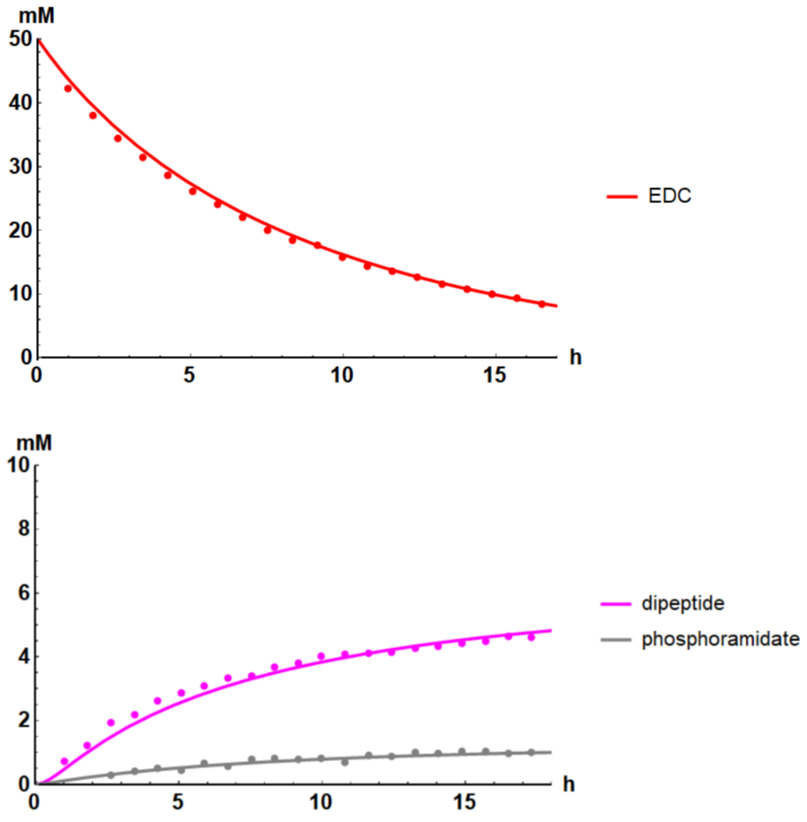

Figure S90. Kinetic plots for the reaction of Gly-A with glycine methyl ester in the presence of Et-p and EDC, as determined in NMR Assay 9; experimental data points and monoexponential fits.

Rate Constants

Table S16. Rate constants obtained for the reactions of the network producing model compound MeO-GlyGly-A from Gly-A and H-Gly-OMe in the presence or absence of ethyl phosphate in assay buffer, as determined by NMR from a series of reactions of increasing complexity.[a]

Assay No.	Rate constant[b]	value[c]
1	k_h1	2.9×10^{-3} h^{-1}
2	$k2$	6.4×10^{-3} mM^{-1} h^{-1}
	$k3$	1.2×10^{-2} mM^{-1} h^{-1}
	$k9$	5.0×10^{-2} h^{-1}
	k_h3	1.1 h^{-1}
3	$k1$	3.9×10^{-3} mM^{-1} h^{-1}
	k_h2	3.9 h^{-1}
4	$k5$	2.9×10^{-2} mM^{-1} h^{-1}
5	$k4$	5.0×10^{-3} mM^{-1} h^{-1}
	$k6$	2.0×10^{-2} mM^{-1} h^{-1}
6	$k7$	9.0×10^{-3} mM^{-1} h^{-1}
7	k_h4	4.7×10^{-3} h^{-1}
8	$k8$	7.0×10^{-3} mM^{-1} h^{-1}

[a] Conditions: 100 mM $MgCl_2$, 100 mM MES buffer, 20 mM ethyl phosphate, 10 mM Gly-A, 20 mM glycine methyl ester, 50 mM EDC, pH 6, 4 °C, and 0.4 mM Gd[DTPA] as relaxation enhancer for ^{13}C nuclei.

[b] For the assignment of rate constants to reactions, see Figure 7A (main manuscript) for a simplified representation of the reaction network or Figure S71 for an unabridged version of it.

[c] Note that both second order and pseudo first order rate constants are shown.

Full flux maps

To illustrate the effect of the additive ethyl phosphate on peptide bond formation, the flux maps shown below were created, one for the reaction network in the absence of ethyl phosphate and on in the presence of this organophosphate. The flux through any given reaction channel was obtained by integration of the reaction equations over the course of 100 h. A reduced level of side reactions (ester and *N*-acyl urea), hydrolysis, and an increased selectivity towards the desired peptide are discernible in the presence of the additive.

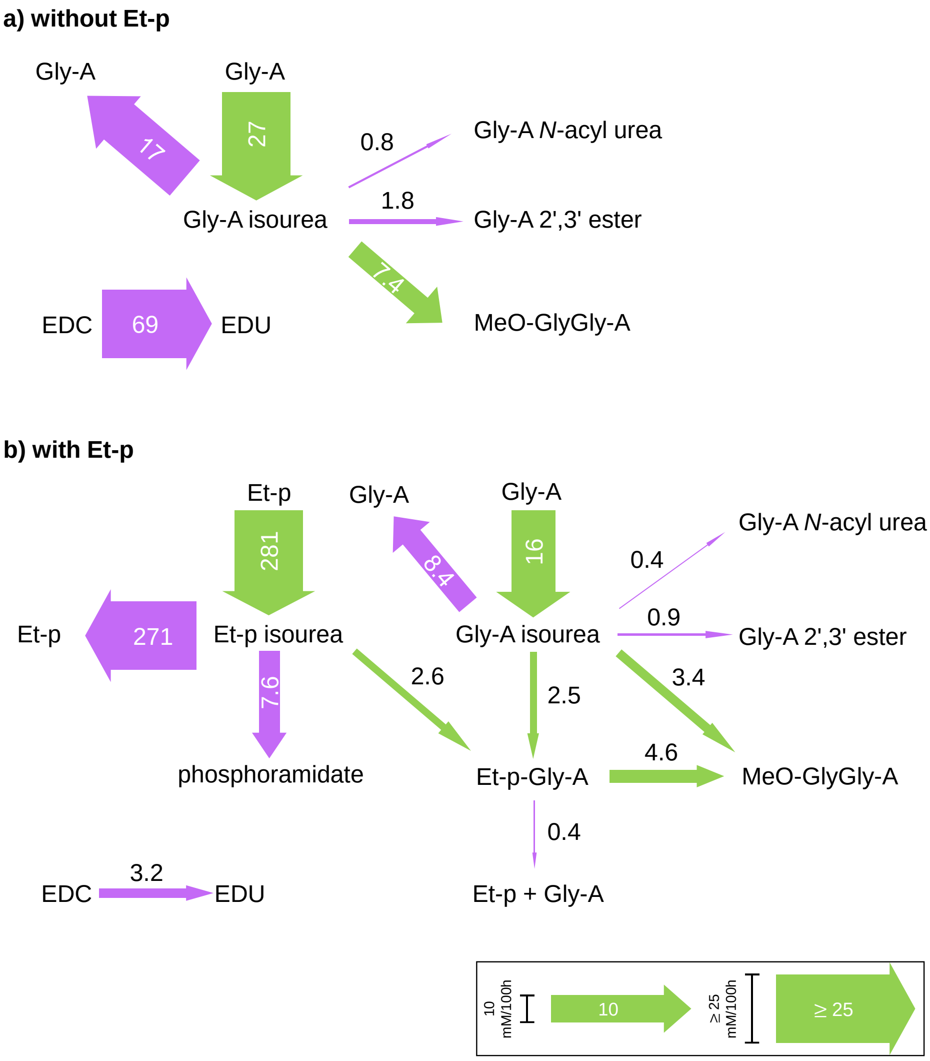

Figure S91. Full version of flux maps for reactions leading to MeO-GlyGly-A in the absence and presence of ethyl phosphate. a) Flux map, as obtained by integrating for 100 h. Green arrows represent steps toward the desired product, purple arrows are for hydrolytic loss and side products. b) Same as a), but in the presence of ethyl phosphate (Et-p). Arrow width, as well as numerically on or above each arrow indicate the respective flux. The inset in the lower right-hand corner shows the width of an arrow representing a flux of 10 mM per 100 h for visual calibration. Fluxes that are greater than 25 mM per 100 h are not resolved graphically.

9. References for Supporting Information

[S1] T. Horn, M. S. Urdea, A Chemical 5′-Phosphorylation of Oligodeoxyribonucleotides that Can Be Monitored by Trityl Cation Release. *Tetrahedron Lett.* **1986**, *27*, 4705−4708.

[S2] M. J. Cavaluzzi, P. N. Borer, Revised UV extinction coefficients for nucleoside-5′-monophosphates and unpaired DNA and RNA, *Nucleic Acids Res.* **2004**, *32*, e13.

[S3] M. Räuchle, G. Leveau, C. Richert, Synthesis of Peptido RNAs from Unprotected Peptides and Oligoribonucleotides via Coupling in Aqueous Solution. *Eur. J. Org. Chem.* **2020**, *45*, 6966–6975.

[S4] B. Jash, P. Tremmel, D. Jovanovic, C. Richert, Single Nucleotide Translation Without Ribosomes. *Nat. Chem.* **2021**, *13*, 751−757.

[S5] S. G. Reußwig, C. Richert, Ribosome-Free Translation up to Pentapeptides via Template Walk on RNA Sequences. *Angew. Chem. Int. Ed.* **2024**, *63*, e202410317.

[S6] D. Jovanovic, P. Tremmel, P. S. Pallan, M. Egli, C. Richert, The Enzyme-Free Release of Nucleotides from Phosphoramidates Depends Strongly on the Amino Acid. *Angew. Chem. Int. Ed.* **2020**, *59*, 20154−20160.

[S7] M. Wang, P. Opare, C. Boddy, Polyketide Synthase Thioesterases Catalyze Rapid Hydrolysis of Peptidyl Thioesters. *Bioorg. Med. Chem. Lett.* **2009**, *19*, 1413−1415.

[S8] D. Sarracino, C. Richert, Quantitative MALDI-TOF Spectrometry of Oligonucleotides and a Nuclease Assay. *Bioorg. Med. Chem. Lett.* **1996**, *6*, 2543−2548.

[S9] B. Jash, C. Richert Templates direct the sequence-specific anchoring of the C-terminus of peptido RNAs. *Chem. Sci.* **2020**, *11*, 3487−3494.

[S10] P. Tremmel, H. Griesser, U. E. Steiner, C. Richert, How Small Heterocycles Make a Reaction Network of Amino Acids and Nucleotides Efficient in Water. *Angew. Chem. Int. Ed.* **2019**, *58*, 13087−13092.

[S11] F. Welsch, E. Kervio, P. Tremmel, C. Richert, Prolinyl Nucleotides Drive Enzyme-Free Genetic Copying of RNA. *Angew. Chem. Int. Ed.* **2023**, *62*, e202307591.
